# Emergence of a Stage-Dependent Human Liver Disease Signature with Directed Differentiation of Alpha-1 Antitrypsin-Deficient iPS Cells

**DOI:** 10.1016/j.stemcr.2015.02.021

**Published:** 2015-04-02

**Authors:** Andrew A. Wilson, Lei Ying, Marc Liesa, Charis-Patricia Segeritz, Jason A. Mills, Steven S. Shen, Jyhchang Jean, Geordie C. Lonza, Derek C. Liberti, Alex H. Lang, Jean Nazaire, Adam C. Gower, Franz-Josef Müeller, Pankaj Mehta, Adriana Ordóñez, David A. Lomas, Ludovic Vallier, George J. Murphy, Gustavo Mostoslavsky, Avrum Spira, Orian S. Shirihai, Maria I. Ramirez, Paul Gadue, Darrell N. Kotton

**Affiliations:** 1 Center for Regenerative Medicine (CReM) of Boston University and Boston Medical Center, Boston, MA 02118, USA; 2 Department of Pathology and Laboratory Medicine, The Children’s Hospital of Philadelphia, Philadelphia, PA 19104, USA; 3 Evans Center for Interdisciplinary Research, Department of Medicine, Mitochondria ARC, Boston University School of Medicine, Boston, MA 02118, USA; 4 Wellcome Trust-Medical Research Council Cambridge Stem Cell Institute, Anne McLaren Laboratory for Regenerative Medicine and Department of Surgery, University of Cambridge, Cambridge CB2 0SZ, UK; 5 Division of Computational Biomedicine and Department of Pathology and Laboratory Medicine, Boston University School of Medicine, Boston, MA 02118, USA; 6 Physics Department, Boston University, Boston, MA 02215, USA; 7 The Pulmonary Center and Department of Medicine, Boston University School of Medicine, Boston, MA 02118, USA; 8 Zentrum für Integrative Psychiatrie, Universitätsklinikums Schleswig-Holstein, Kiel 24105, Germany; 9 Cambridge Institute for Medical Research, Cambridge CB0 2XY, UK

## Abstract

Induced pluripotent stem cells (iPSCs) provide an inexhaustible source of cells for modeling disease and testing drugs. Here we develop a bioinformatic approach to detect differences between the genomic programs of iPSCs derived from diseased versus normal human cohorts as they emerge during in vitro directed differentiation. Using iPSCs generated from a cohort carrying mutations (PiZZ) in the gene responsible for alpha-1 antitrypsin (AAT) deficiency, we find that the global transcriptomes of PiZZ iPSCs diverge from normal controls upon differentiation to hepatic cells. Expression of 135 genes distinguishes PiZZ iPSC-hepatic cells, providing potential clues to liver disease pathogenesis. The disease-specific cells display intracellular accumulation of mutant AAT protein, resulting in increased autophagic flux. Furthermore, we detect beneficial responses to the drug carbamazepine, which further augments autophagic flux, but adverse responses to known hepatotoxic drugs. Our findings support the utility of iPSCs as tools for drug development or prediction of toxicity.

## Highlights

• Patient iPSC-hepatic cells model disease features that normalize with gene editing
• Disease-specific transcriptomic signature emerges only upon hepatic differentiation
• Patient iPSC-hepatic cells are used to test response to drugs or toxicity in vitro

## Graphical Abstract

**Figure:**
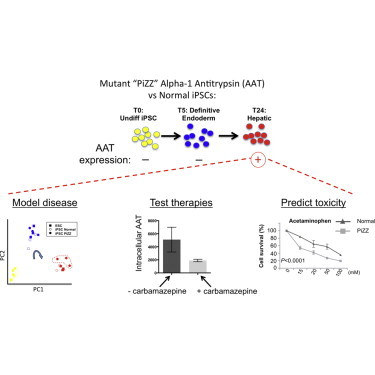

## Teaser

In this report, Kotton and colleagues examine the global transcriptomic and epigenomic programs of iPSCs derived from diseased (AAT-deficient PiZZ) or normal human cohorts during directed differentiation. The PiZZ transcriptome diverges from controls upon reaching hepatic stage, when AAT is first expressed, providing potential clues to liver disease pathogenesis. PiZZ iPSC-hepatic cells model key liver disease features and demonstrate both therapeutic drug responsiveness and sensitivity to toxic agents.

## Introduction

Alpha-1 antitrypsin deficiency (AATD) is a common genetic cause of both liver and lung disease affecting an estimated 3.4 million patients worldwide (de Serres, 2002). The most common disease variant is caused by an inherited single base pair mutation of the *SERPINA1* gene that results in a glutamate to lysine substitution (Glu342Lys) and production of a mutant version of the protease inhibitor AAT, known as Z AAT (Brantly et al., 1988). Z AAT protein is prone to misfolding and polymerization and has reduced capacity to inactivate neutrophil elastase, its primary substrate, resulting in both toxic gain-of-function and loss-of-function phenotypes (Brantly et al., 1988; Crystal, 1990; Lomas et al., 1992; Perlmutter and Pierce, 1989). AATD has proven difficult to model experimentally in mice and in human primary or immortalized cells, a factor that has limited the progress of research aimed either at elucidating mechanisms of disease or developing new treatment approaches. Studies based on transgenic PiZ mice or immortalized cell lines engineered to express the human mutant Z AAT allele or on primary human hepatocytes have provided significant insights into the pathogenesis of AATD-associated liver disease. These studies have demonstrated that polymerization of Z AAT protein in the ER results in activation of an ER overload response (Hidvegi et al., 2005; Lawless et al., 2004), characterized by chronic activation of the proinflammatory transcription factor NF-κB (Pahl and Baeuerle, 1995), together with activation of ER stress-specific caspases (Hidvegi et al., 2005). Each of these models, however, has shortcomings that potentially limit its ability to delineate the mechanisms of a disease that develops over time in human liver tissue.

Recently, the discovery of induced pluripotent stem cells (iPSCs) (Takahashi and Yamanaka, 2006) has made it possible to model a variety of genetic diseases in vitro using patient-derived stem cells (Ebert et al., 2009; Park et al., 2008; Rashid et al., 2010). The differentiated progeny of patient-derived iPSCs provide disease-relevant cells in an individual patient’s genetic background, potentially allowing personalized, in vitro assessments of disease pathogenesis and treatment responsiveness. As with human clinical trials, however, studies utilizing multiple patient-derived iPSC lines introduce the complexity of genetic variability. This experimental approach increases the likelihood that findings will be generalizable to a population rather than specific to an individual, but also potentially decreases the signal-to-noise ratio.

Here we sought to apply an iPSC-based approach to study generalizable effects of the Z mutation, rather than the effects of any single individual’s genetic background. To do so, we incorporated iPSC lines derived from multiple individuals homozygous for the Z allele (termed PiZZ), ensuring the inclusion of genetic heterogeneity. We found that the transcriptional profile of iPSCs derived from individuals homozygous for the Z allele diverges from normal controls only upon differentiation to the hepatic stage, when the AAT gene is expressed. Expression of 135 genes distinguishes PiZZ iPSC-hepatic cells from controls at this stage, providing potential clues to liver disease pathogenesis. PiZZ iPSC-hepatic cells model key features of AATD-associated liver disease, including intracellular accumulation and reduced secretion of AAT protein as well as increased autophagic flux. Augmented autophagic flux can be further enhanced in iPSC-hepatic cells upon treatment with the drug carbamazepine (CBZ), an observation first made in transgenic PiZ mice (Hidvegi et al., 2010) that has important implications for treating patients with AATD-related liver disease. Finally PiZZ iPSC-hepatic cells exhibit increased sensitivity to a panel of hepatotoxic drugs, including the common analgesic acetaminophen, confirming their potential application as tools for drug discovery or prediction of toxicity.

## Results

To develop iPSC-based model systems of disease, we first prepared a bank of >60 iPSC clones (ten clones per donor; partial set and reprogramming methodology described previously [Mills et al., 2013; Somers et al., 2010]) derived from the dermal fibroblasts of three control individuals without any known disease and three recruited volunteers previously diagnosed with AATD due to homozygous inheritance of mutant Z alleles encoding the AAT protease inhibitor (PiZZ genotype, a common monogenic cause of hepatic cirrhosis [Eriksson et al., 1986]). Pluripotency of the resulting cells was confirmed in teratoma assays (Somers et al., 2010), as well as by Pluritest global transcriptomic analysis (Müller et al., 2011; Figure S1).

We previously published microarray profiling of the global transcriptomes of nine iPSC lines made from three normal donors (three iPSC lines/donor) and three embryonic stem cell (ESC) lines. We determined that donor-to-donor genetic variability in stem cell phenotype (defined as the global transcriptome expressed in the pluripotent/undifferentiated state) rather than clone-to-clone variability per donor was most responsible for variance in gene expression among cell lines (Mills et al., 2013). In addition to gene expression, we found the propensity to differentiate into a particular fate was also due mainly to genetic background as opposed to clonal variability, consistent with others’ work focused on the relative contribution of donor, cell type of origin, and reprogramming approach to differentiation efficiency (Kajiwara et al., 2012). These data prompted us to select only a single clone per donor for all disease-modeling experiments, as individual clones of a given genetic background tend to behave similarly.

### PiZZ iPSC-Hepatic Cells Demonstrate Accumulation of Intracellular AAT Protein and Expression of a Transcriptomic Disease-Specific Signature

**Figure 1:**
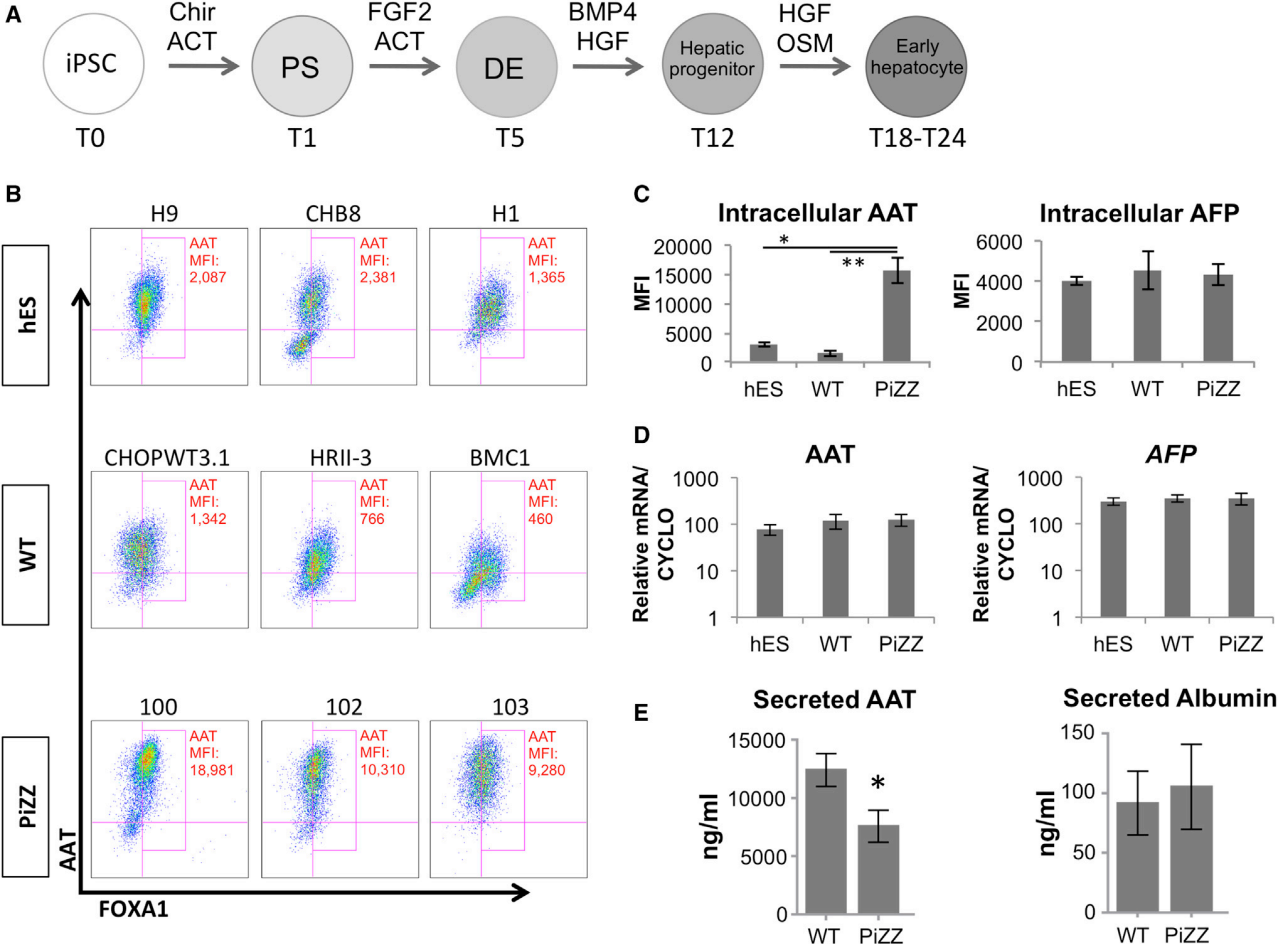
Patient-Derived PiZZ iPSC-Hepatic Cells Exhibit Intracellular AAT Retention (A) Schematic overview of iPSC-hepatic directed differentiation protocol. ^∗^p < 0.05, ^∗∗^p < 0.01 by one-way ANOVA. (B) Flow cytometry of fixed, permeabilized iPSC-hepatic cells using anti-AAT and anti-FOXA1 antibodies in nine genetically distinct ESC or iPSC lines at T24. (C) MFI of AAT and AFP in PiZZ iPSC-hepatic cells compared to WT iPSC- or ESC-hepatic cells. (D) Quantification of AAT (*SERPINA1*) and *AFP* mRNA levels in iPSC-hepatic cells. (E) ELISA of secreted AAT and albumin in cell supernatants at T24. ^∗^p < 0.05 by two-tailed t test. PS, primitive streak; DE, definitive endoderm; ACT, Activin A; Chir, Chir99021; OSM, oncostatin M. n = 3 independent experiments. Data are represented as mean ± SEM.

We sought to test the hypothesis that a single transgene-free iPSC clone from each donor could be used to detect disease-specific differences between the normal cohort and the PiZZ cohort, anticipating that differences would emerge only at a developmental stage in which the mutant AAT gene is expressed. Employing single iPSC clones from each normal or PiZZ individual as well as normal ESC control clones (n = 3 per group), we utilized sequential growth factor stimulation with serum-free conditions that we established previously (Cheng et al., 2012; Somers et al., 2010) for the directed differentiation of pluripotent cells into definitive endoderm followed by early hepatocyte-like lineages (Figure 1A; Figure S2).

We monitored all nine cell lines across three time points of hepatic directed differentiation, representing three developmental stages: (1) undifferentiated (T0), (2) definitive endoderm (T5), and (3) early hepatocyte (T24). No significant differences in differentiation efficiency were observed between PiZZ and normal lines as measured by flow cytometric quantitation of markers of pluripotency (SSEA3, TRA-1-81), definitive endoderm (CKIT/CXCR4), and hepatic (AAT/FOXA1) lineages at each developmental stage (Figure 1B; Figure S3). As previously reported by others (Choi et al., 2013; Rashid et al., 2010), we found intracellular accumulation of AAT protein in all three of our PiZZ iPSC lines at the hepatic stage (T24). The mean fluorescence intensity (MFI) of immunostained intracellular AAT protein measured by flow cytometry was logarithmically elevated in iPSC-hepatic cells from three of three PiZZ individuals compared to the six other lines (Figures 1B and 1C). This accumulation was neither due to differences in AAT (*SERPINA1*) expression at the RNA level nor to generalized accumulation of liver-related proteins, such as AFP (Figures 1C and 1D). AAT secretion was significantly decreased in the PiZZ iPSC-hepatic cells while albumin secretion was similar to controls (Figure 1E), suggesting that the increase in intracellular AAT was due to a failure to properly process and secrete this protein.

**Figure 2:**
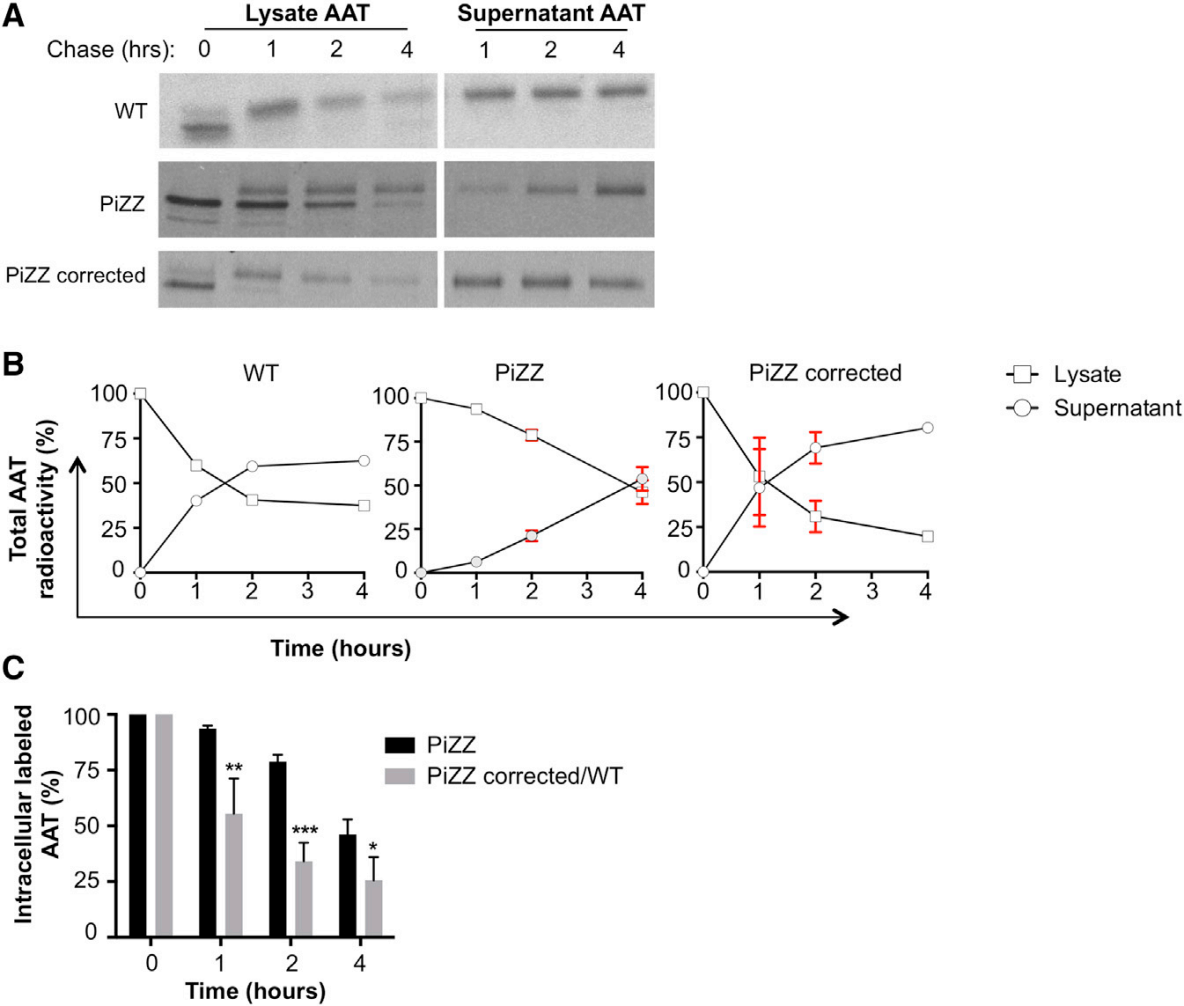
Pulse-Chase Radiolabeling Demonstrates Altered Post-translational AAT Processing and Secretion Kinetic in PiZZ iPSC-Hepatic Cells (A) Quantification of radiolabeled AAT protein from cell lysates or supernatants at indicated time points after ^35^S-Met/Cys radiolabeling. Both the 52-kDa native AAT protein and its 55-kDa mature are detected in WT, PiZZ, and gene-corrected PiZZ (to PiMM, following ZFN-mediated correction of the Z mutation) iPSC-hepatic cells. (B) Kinetic of aggregate cell lysate and supernatant radiolabeled AAT secretion data represented in (A). (C) Quantification of aggregate intracellular radiolabeled AAT protein during the chase period. n = 4 (PiZZ) or n = 3 (PiZZ corrected/WT) independent experiments. Data are represented as mean ± SD. ^∗^p < 0.05, ^∗∗^p < 0.01, ^∗∗∗^p < 0.001 by two-tailed t test.

To address definitively whether accumulation of intracellular protein observed in PiZZ iPSC-hepatic cells was due to reduced AAT flux and specifically resulted from the Z mutation, we performed pulse-chase labeling experiments. Employing this classical assay for defining the kinetics of AAT protein processing and secretion, we utilized an AATD iPSC line in which both mutant Z alleles had undergone zinc-finger-mediated gene correction, comparing the corrected clone to its parental, syngeneic PiZZ line (Yusa et al., 2011). Published studies in PiZ mice (Graham et al., 1990), HeLa cells (Hidvegi et al., 2005), and primary human fibroblasts (Wu et al., 1994) engineered to express the mutant Z protein previously have demonstrated altered posttranslational modification of AAT protein and delayed secretion in Z AAT-expressing cells. In keeping with these heterologous models, we found delayed processing of the native 52-kDa AAT protein to its mature 55-kDa form in PiZZ iPSC-hepatic cells together with delayed AAT secretion compared to its gene-corrected parental line and a wild-type (WT) control line (Figures 2A–2C).

**Figure 3:**
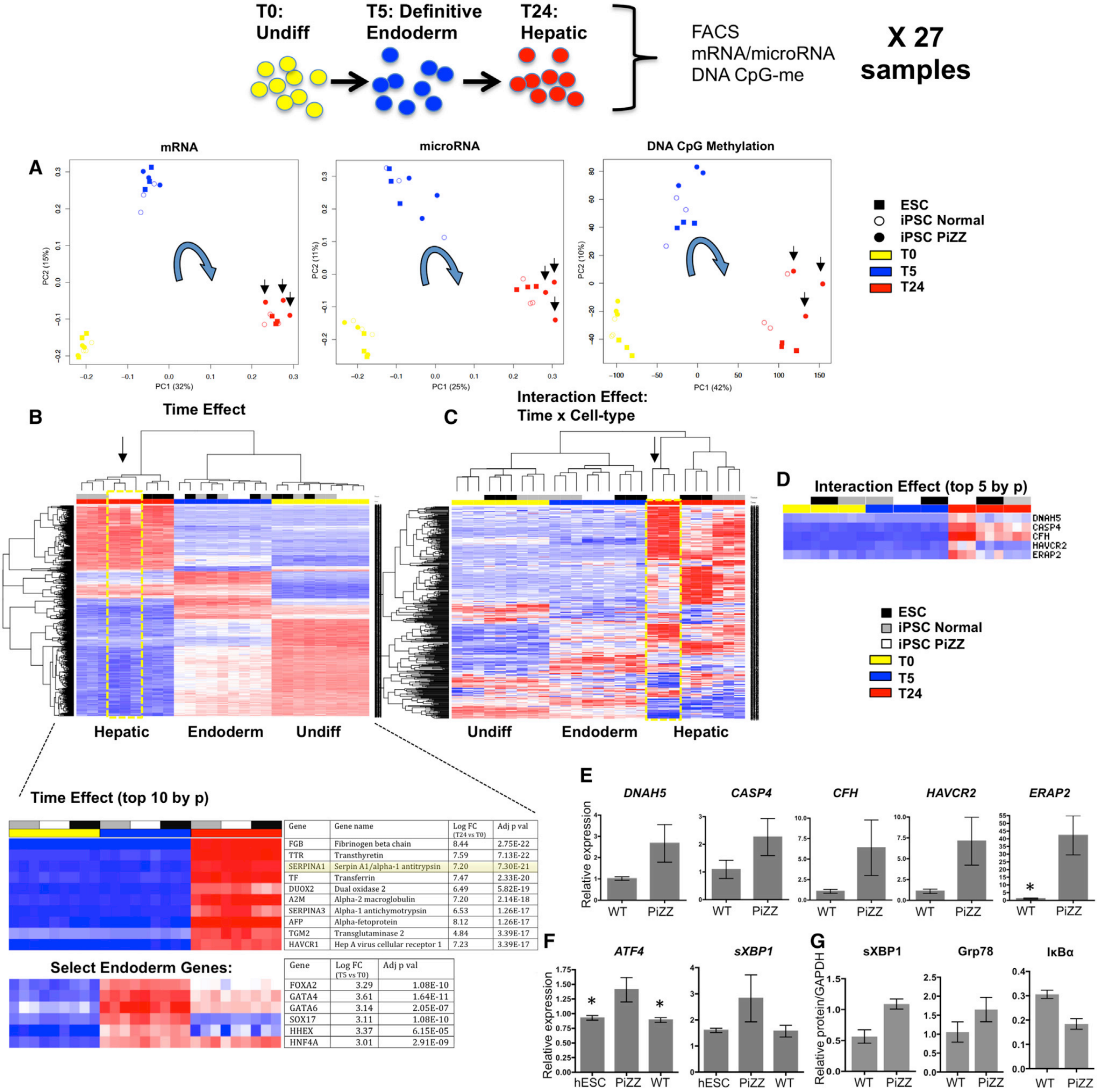
Genome-wide Transcriptomic and Epigenomic Analysis of PSCs at Key Developmental Stages ESCs (n = 3) and patient-derived normal/WT (n = 3) or PiZZ (n = 3) iPSCs were analyzed at T0, T5, and T24 for a total of 27 samples. (A) PCA demonstrates clustering predominantly by developmental stage (arrows indicate hepatic-stage PiZZ cells). (B) Unsupervised hierarchical clustering of 27 samples analyzed by two-way ANOVA for time and cell type effects demonstrates differential gene expression with directed differentiation. The top ten genes upregulated with differentiation (middle) as well as expression levels of select endoderm genes at T5 (bottom) are listed. (C) Analysis of gene expression differences among cell types that are modulated by differentiation (interaction effect of time and cell type) reveals 419 differentially expressed genes (FDR < 0.25; 85 genes at FDR < 0.1). (D) Expression levels of the top five genes differentially expressed between PiZZ and normal iPSC-hepatic cells as identified by a post hoc moderated t test of the interaction effect gene set. (E) qPCR validation of relative mRNA expression levels of genes from (D). n = 3 independent experiments. ^∗^p < 0.05 by two-tailed t test. (F) qPCR quantification of *ATF4* and spliced *XBP-1* mRNA expression at early hepatic stage (T18) in ESCs and PiZZ or normal iPSCs. n = 3 independent experiments. ^∗^p < 0.05 by one-way ANOVA. (G) Densitometric quantification of sXBP-1, Grp78, and IκBα protein expression levels by western blot of whole-cell lysates prepared from PiZZ and normal iPSCs (n = 3 biological replicates per group) at T16–T18 and normalized to GAPDH. Data are represented as mean ± SEM.

Having demonstrated decreased AAT protein flux and intracellular AAT accumulation in disease-specific iPSC-hepatic cells, we next performed microarray analyses of the global transcriptomes (mRNA and microRNA [miRNA]) and DNA methylomes (methylated CpG dinucleotides) of these nine cell lines at each differentiation stage (Figure 3). Principal component analysis (PCA) of mRNA, miRNA, and DNA microarrays from these 27 samples revealed tight clustering of samples by developmental stage rather than by donor (Figure 3A). To statistically interrogate the kinetics of global gene expression of each cell line during directed differentiation to liver, we employed two-way ANOVA of all 27 samples to identify: (1) genes that are differentially expressed among days 0, 5, and 24 of differentiation (time effect); (2) genes that are differentially expressed among ESC, normal iPSC, and PiZZ iPSC lines (cell type effect); and (3) gene expression differences among cell types that are modulated by differentiation (interaction effect of cell type and time). We have described previously this approach of comparing the entire gene expression kinetic with differentiation as a robust method for detecting subtle differences between iPSCs and ESCs when using small sample sizes (Christodoulou et al., 2011). Directed differentiation was associated with a large number of gene expression changes (11,232 of ∼30,000 probe sets were significantly associated with the time effect at false discovery rate (FDR) < 0.01; Figure 3B). Importantly, all master endodermal transcriptional regulators assessed (*FOXA2*, *GATA4* and *6*, *SOX17*, *HEX*, and *HNF4A*) were upregulated as expected at T5, and known markers of hepatocytes (AAT, *TTR*, *FBG*, *TF*, *ALB*, CYP450 enzymes, and hepatitis virus receptors) were upregulated at T24 (Figure 3B; Figure S2). As expected, AAT was not detectably expressed at T0 or T5; however, AAT was the third most significantly upregulated gene of 30,000 tested transcriptomic probe sets in the genome at the hepatic stage (ranking by FDR-adjusted p value; Figure 3B).

To develop a putative PiZZ disease-specific transcriptomic signature based on this iPSC model of differentiation, we next analyzed the interaction effect. We identified differentially expressed genes based on the interaction of time and cell type (419 genes at FDR < 0.25; Figure 3C; 85 genes at FDR < 0.1). To determine which cell type and differentiation stage were responsible for differences in gene expression during directed differentiation, we first employed unsupervised clustering of the top 1,000 genes differentially expressed with time, and we found the 27 samples clustered predominantly by developmental stage. However, clustering by cell type emerged at the hepatic stage of differentiation based on the height of the clustered dendogram at T24 (Figure 3B). Second, we performed a post hoc analysis of the cell type effect using moderated t tests to identify which cell type was responsible for the cell type effect. These tests revealed that the cell type effect was due entirely to the PiZZ iPSC clones, with no cell type differences between ESCs versus normal iPSCs. Third, we performed post hoc moderated t tests of the interaction effect to identify 135 differentially expressed transcripts at T24 that distinguished normal versus PiZZ iPSC-hepatic cells, hereafter referred to as a disease-specific transcriptomic signature (Figure 3D; Table S1). The five genes most differentially expressed in this list were then validated by qPCR (*DNAH5*, *CASP4*, *CFH*, *HAVCR2*, and *ERAP2*; Figures 3D and 3E). Together these statistical analyses indicated that the subtle divergence in gene expression kinetics among cell types undergoing hepatic directed differentiation is due mainly to the divergence of PiZZ disease-specific clones upon reaching the hepatic stage (Figures 3C and 3D).

We next looked at differences in miRNA (by miRNA arrays) and methylation status (by genome-wide Illumina 450K methylation array) of ESCs and iPSCs during directed differentiation. The large variance in miRNA expression profiles precluded finding any statistically significant miRNA expression differences between diseased and normal cells at any stage; however, clear differences were evident in all cell types based on developmental stage (Table S2; Figure 3A). In keeping with the mRNA expression differences that emerged between PiZZ iPSCs versus normal iPSCs and ESCs during directed differentiation, the number of DNA methylation differences between diseased and normal pluripotent stem cells also increased from 23 at T0 to 195 at T24 (Table S2). To determine whether differences in DNA methylation could potentially account for altered gene expression in PiZZ iPSC-hepatic cells, we next analyzed CpG sites in the promoter regions of differentially expressed genes (Figure 3A). At the hepatic stage (T24), one or more CpG sites were differentially methylated in 13 of the 135 interaction effect genes (Table S3) comprising the PiZZ disease-specific transcriptomic signature. CpG methylation and expression levels of these genes were significantly anti-correlated. While not in themselves proof of causality, together these findings suggest an association between differences in gene expression in hepatic-stage PiZZ and normal iPSCs and alterations in the methylation state of the promoter regions of a subset of these genes.

### Hepatic Pathways Responding to Intracellular Accumulation of Mutant Z AAT

Next we sought to employ our disease-specific transcriptomic signature and the iPSC model system to better understand the pathogenesis of AATD-related liver disease. Based on established mouse models, human liver biopsy specimens, and human heterologous cell line studies, mutated Z AAT protein has been demonstrated to induce liver disease through a toxic gain-of-function resulting predominantly from the accumulation of misfolded, insoluble aggregates of mutant protein in the rough ER (Lomas et al., 1992). Indeed, our interaction effect gene signature revealed differential expression of several individual gene markers associated with oxidant stress or known to localize to the ER. For example, 27 genes on the interaction list, including *ERAP2*, were grouped under the gene ontology heading of ER (Figure 3; Table S1). Notably, the second most significantly differentially expressed transcript within the interaction effect disease-specific signature was the ER stress-specific caspase family member, caspase-4, validated by qPCR to be upregulated in PiZZ iPSC-hepatic cells compared to controls (Figure 3E). Activation of mouse caspase-12 and human caspase-4, its putative homolog (Hitomi et al., 2004), have been demonstrated in Z AAT-expressing mouse and human cell lines and in PiZ mouse livers (Hidvegi et al., 2005).

Markers of the unfolded protein response (UPR) were not present in the PiZZ disease-specific signature in concordance with previous studies that have detected no evidence of hepatic UPR resulting from misfolded Z AAT in mouse models or cell lines or human liver biopsy specimens (Hidvegi et al., 2005). Since T24 represents a time in culture >1 week after the onset of initial AAT protein expression (Figure S3), we sought to determine whether a UPR was detectable in the disease-specific cells closer to the time of initial expression of the mutant Z AAT protein. Indeed, by qPCR at T18 of differentiation, we found significant upregulation of *ATF4* and spliced *XBP-1* mRNA only in the PiZZ cells (Figure 3F). To evaluate whether these findings extended to differences in protein expression, we next performed western blots on whole-cell lysates collected from T16–T18 cells, and we found increased levels of spliced XBP-1 and the ER resident chaperone molecule Grp-78 in PiZZ cells compared to controls. Because the UPR is likewise known to activate NF-κB via degradation of its inhibitor, IκBα, mediated by IRE-1 oligomerization and binding to TRAF2 (Kaneko et al., 2003), we also assayed IκBα and found it to be decreased in PiZZ cells at this stage of differentiation (Figure 3G; Figure S4). Together, these findings are consistent with activation of the UPR in hepatocytes by misfolded Z AAT protein, potentially in a manner that is context specific.

**Figure 4:**
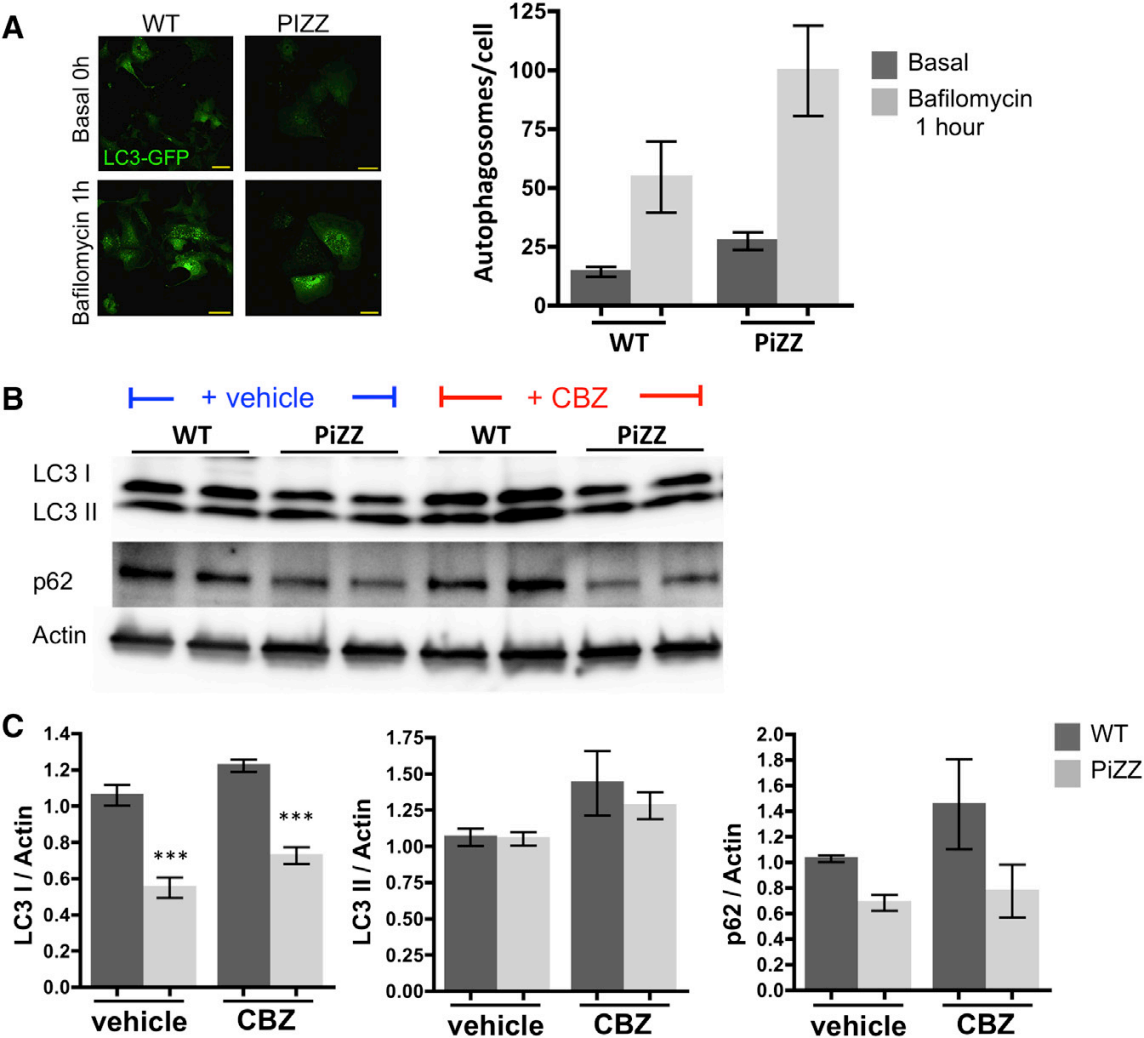
Enhanced Autophagic Flux in PiZZ iPSC-Hepatic Cells (A) Differentiating iPSCs were transduced with a lentivirus encoding an LC3-GFP fusion protein. Following differentiation to the hepatic stage, LC3-GFP+ autophagosomes are quantified in the presence or absence of bafilomycin, an inhibitor of lysosomal fusion. Data are representative of one to three biological replicates per group per condition. Scale bars, 50 μM. (B) Protein levels of the autophagosome components LC3-I and -II, together with p62 are quantified by western blot in WT and PiZZ iPSC-hepatic cells in the presence and absence of CBZ treatment. (C) Protein levels from (B) are displayed graphically and normalized to actin (n = 3 biological replicates per group). Data are represented as mean ± SEM. ^∗∗∗^p < 0.001 by two-tailed t test.

The autophagy pathway is a key cellular mechanism thought to be responsible for processing intracellular Z AAT protein aggregates, based on documented increases in autophagic flux in mammalian cell lines and mouse models featuring overexpression of human Z AAT (Teckman and Perlmutter, 2000; Teckman et al., 2002). Indirect evidence of altered autophagy in humans derives from observation of increased numbers of autophagic vesicles in PiZZ liver biopsy specimens (Teckman and Perlmutter, 2000). We therefore sought to monitor and manipulate autophagic flux in iPSC-derived hepatic lineages. We first transduced PiZZ versus normal iPSC-hepatic cells with a lentiviral LC3-GFP reporter (Twig et al., 2008) to count autophagosomes per cell. After treating T18 cells for 1 hr with the autophagosome clearance blocker bafilomycin, we observed more autophagosomes per cell in the PiZZ group (n = 50–60 cells per group; Figure 4A). Because these findings are consistent both with increased autophagosome formation and clearance in PiZZ iPSC-hepatic cells, we next determined LC3-I, LC3-II, and p62 protein levels in iPSC-hepatic cells by western blot analysis (Figures 4B and 4C). We found decreased p62 and decreased LC3-I with no significant changes in LC3-II in PiZZ iPSC-hepatic cells compared to controls, indicating increased clearance of autophagosomes in PiZZ cells. Together, these data indicate that increased autophagic flux is present in the PiZZ iPSC-hepatic cells, consistent with prior studies in other models (Hidvegi et al., 2010; Teckman and Perlmutter, 2000).

To determine whether drug treatment could further augment autophagic flux in iPSC-hepatic cells, we employed CBZ treatment based on publications by Perlmutter et al. who found this drug augments flux and reduces the burden of accumulated intracellular Z AAT protein in transgenic mice and heterologous cell lines. We observed increased LC3-II and LC3-I in both normal and PiZZ iPSC-hepatic cells, indicating increased autophagosome formation with CBZ treatment. The differences between normal and PiZZ cells were maintained after treatment, indicating increased clearance and increased formation of autophagosomes in PiZZ cells. However, the increase in p62 after CBZ treatment, likely resulting from increased autophagosome formation and accumulation, was greater in normal cells (∼40%) than in PiZZ cells (∼10%) (Figures 4B and 4C). Together, these findings provide evidence in human patient samples that supports and extends findings from prior non-human model systems demonstrating increased baseline autophagic flux in PiZZ-producing cells that is further augmented by CBZ.

### iPSC-Hepatic Cells Serve as a Model of Disease-Specific Drug Therapy

**Figure 5:**
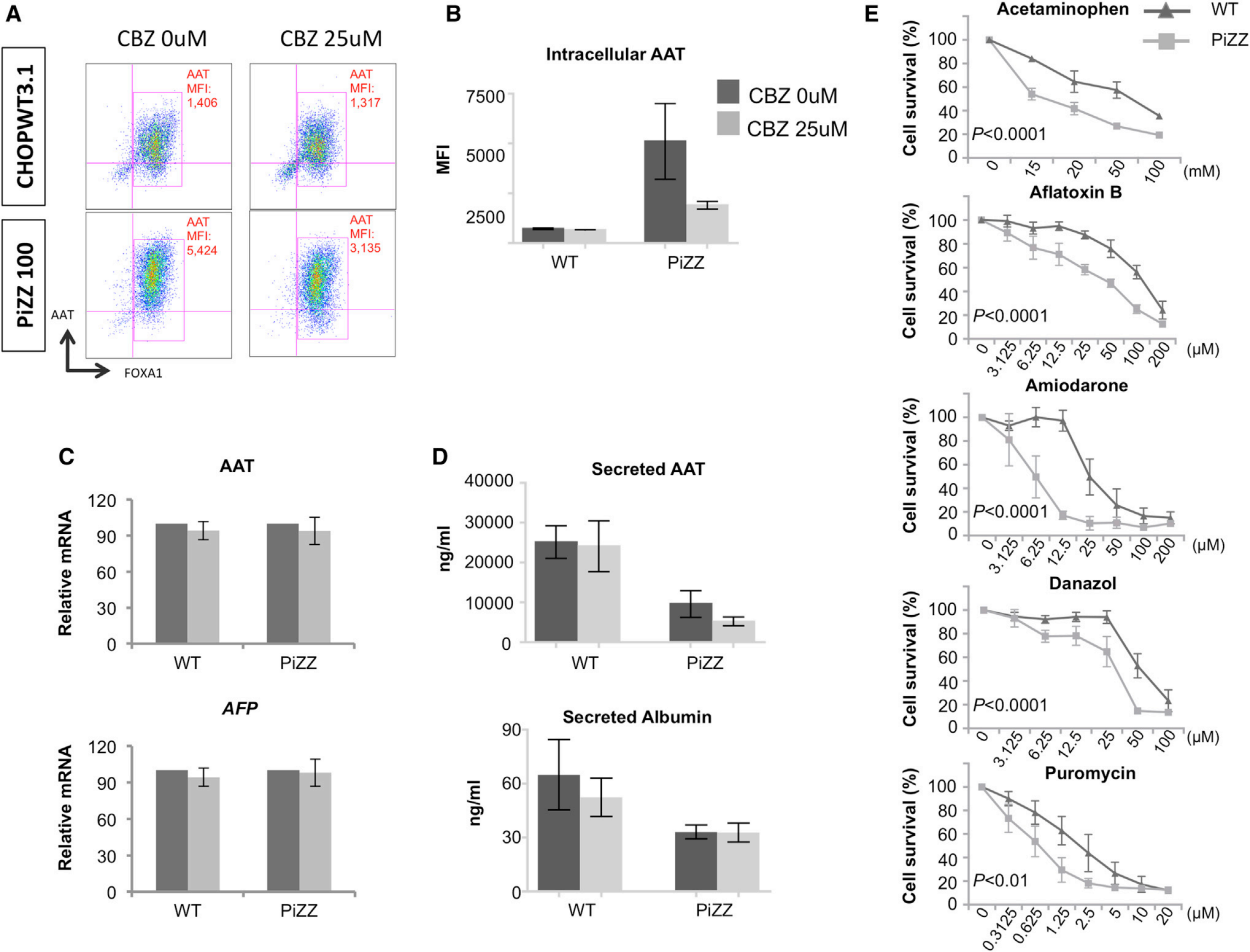
PiZZ and WT iPSC-Hepatic Cells Diverge in Their Response to Drug Treatment (A) Representative flow cytometric analysis of AAT MFI in WT and PiZZ iPSC-hepatic cells (T24) following a 48-hr treatment with control vehicle or CBZ. (B) Aggregate data from three WT and three PiZZ lines, including data represented in (A), are quantified graphically. n = 3 independent experiments. (C) AAT (*SERPINA1*) (top) and *AFP* (bottom) mRNA expression levels with and without CBZ treatment, expressed as a percentage of untreated levels. (D) Concentration of AAT (top) and albumin (bottom) in iPSC-hepatic cell supernatants measured by ELISA. n = 3 independent experiments. (E) Viability of PiZZ and WT iPSC-hepatic cells is quantified by MTT assay after incubation with acetaminophen (4 hr) or aflatoxin B, amiodarone, danazol, or puromycin (4 days) at the indicated concentrations. Survival percentage at each concentration is determined as a percentage of vehicle control treatment. n = 3 independent experiments. Data are represented as mean ± SEM. P values represent contribution of cell type to variance as determined by two-way ANOVA.

Having demonstrated that CBZ is able to augment autophagic flux in human iPSC-hepatic cells, we next sought to determine whether augmented flux would decrease intracellular AAT accumulation in PiZZ cells. We found all three PiZZ donor lines demonstrated CBZ dose-dependent amelioration in accumulation of intracellular AAT protein, whereas no significant difference was observed in the low levels of intracellular AAT in normal cells before versus after treatment (Figures 5A and 5B; Figure S5). CBZ treatment did not result in changes in expression of AAT or differentiation marker genes, indicating that the observed reduction in intracellular AAT was not due to the inhibition of AAT transcription or to suppressed hepatic differentiation of iPSCs (Figure 5C). Finally, CBZ did not affect levels of secreted AAT (or an alternative hepatic secreted protein, albumin) in iPSC-hepatic cell supernatants (Figure 5D).

### PiZZ iPSC-Hepatic Cells Display Increased Susceptibility to Drug-Induced Toxicity

A hallmark of diseased hepatocytes in humans is increased susceptibility to environmental insults or drug toxicity compared to normal hepatocytes. While iPSC-hepatic cells recently have been evaluated as a potential platform for detecting drug toxicity (Sjogren et al., 2014; Szkolnicka et al., 2014), the relative sensitivity of diseased and normal differentiated iPSCs to hepatotoxic agents has not to our knowledge been examined. Hence, we sought to test whether PiZZ iPSC-hepatic cells are more susceptible to the toxic effects of a common pharmaceutical analgesic, acetaminophen. Indeed, we found PiZZ iPSC-hepatic cells were more susceptible to acetaminophen-induced toxicity than normal controls, as evidenced by lower viability at each dose of acetaminophen tested (Figure 5E). To evaluate the specificity of this finding, we extended our evaluation to include a panel of four additional drugs (amiodarone, danazol, puromycin, and aflatoxin-B) known to cause hepatotoxicity through several mechanisms. In each case, PiZZ cells were more sensitive than normal control iPSC-hepatic cells to drug-induced toxicity, consistent with a broad susceptibility to hepatotoxic agents (Figure 5E).

## Discussion

In summary, our results demonstrate the application of iPSCs from multiple donors to model disease, predict drug efficacy or toxicity, and unveil mechanisms not easily studied in vivo in humans or mouse models. We present a bioinformatic approach to defining disease-specific signatures for monogenic diseases based on the delineation of a global transcriptomic signature that emerges in diseased cells only upon reaching the developmental stage at which the mutant protein is expressed. Using this approach, we found PiZZ iPSC lines did not differ from control iPSCs or ESCs when in the undifferentiated or endodermal states but diverged at the hepatic stage, exhibiting evidence of intracellular accumulation of mutant AAT protein, a transcriptomic disease-specific signature that likely represents the downstream effect of accumulated protein aggregates, and activation of pathways known to respond to the burden of misfolded intracellular protein, such as augmented autophagic flux.

In these studies, we included multiple donors in each cohort, rather than multiple isogenic clones, in an approach intended to mimic the design of human clinical trials. This allowed us to determine whether, within a genetically diverse cohort, we could detect disease-specific differences emerging above the known phenotypic variation among normal individuals. Indeed, we were able to detect disease-state-specific differences in gene expression among cohorts, in addition to some differences in levels of intracellular protein accumulation among clones, that could represent genetic differences in protein processing or the cellular response to misfolded proteins that has been postulated to exist among individuals (Pan et al., 2009; Wu et al., 1994).

Human iPSC-hepatic cells derived using our differentiation protocol were similar to primary human fetal hepatocytes in terms of expression levels of a subset of hepatic genes. These results are consistent with other published protocols (Rashid et al., 2010; Si-Tayeb et al., 2010) demonstrating differentiation of hepatic cells that were incomplete in their maturity, as evidenced by persistent, high levels of *AFP* expression that were similar to fetal levels in our experiments. This hurdle in directing differentiation of pluripotent stem cells to fully mature differentiated cells has been seen across germ layers and cell types (Baxter et al., 2015; Smith et al., 2013), reflects the general state of the field, and is the focus of a growing number of investigators (Ogawa et al., 2013; Shan et al., 2013). As our data and the published literature suggest (Leung et al., 2013; Rashid et al., 2010; Suzuki et al., 2014), however, the ability to fully mature a cell in vitro might not be necessary to model and study key disease features when disease-causative genes are expressed at high levels.

We used our iPSC human disease model to assess both well-accepted and controversial pathways for handling protein misfolding that have been interrogated using other approaches. Our report utilizes the classical pulse-chase labeling technique to quantify the kinetics of AAT processing and secretion using human patient-derived hepatocyte-like cells, demonstrating the ability of iPSC-hepatic cells to model a key feature of Z AAT protein-driven cellular dysfunction. In cells accumulating misfolded, insoluble Z AAT protein polymers, the autophagy pathway is activated in an attempt to deal with this toxic protein accumulation. Our studies document increased formation as well as increased clearance of autophagosomes in PiZZ iPSC-hepatic cells, consistent with augmented autophagic flux. These findings are in accord with those previously observed in mouse embryonic fibroblasts (MEFs), cell lines, and transgenic mice overexpressing human Z AAT. Increased autophagosome numbers have been observed in liver biopsy specimens from PiZZ individuals (Teckman and Perlmutter, 2000), but it has not been possible previously to measure flux in their tissues. Our report extends to human hepatic cells the observation made in PiZ transgenic mice (Hidvegi et al., 2010) that further CBZ-induced augmentation of this flux ameliorates intracellular accumulation of mutant protein.

A second cellular stress pathway implicated in the setting of accumulated intracellular Z AAT protein is the UPR, postulated to link Z AAT polymer-induced cellular injury and downstream development of liver disease, a poorly understood progression (Lawless et al., 2004; Perlmutter et al., 2007). Previous studies of PiZZ liver disease in other model systems have not detected a UPR, despite its known role in the cellular response to high volumes of protein misfolding (Hidvegi et al., 2005). Our detection of a UPR, early during the hepatic differentiation of PiZZ iPSCs, thus contrasts with these studies, though it is in keeping with recent patient-based findings in circulating monocytes from PiZZ individuals (Carroll et al., 2010).

The potential of iPSC-based prediction of drug toxicity is a topic that has generated wide interest as protocols for directed differentiation have progressed. Our finding that PiZZ iPSC-hepatic cells exhibit increased sensitivity to an assortment of hepatotoxic drugs, including the analgesic acetaminophen, are in accord with the common clinical recommendation that patients with liver disease limit acetaminophen intake and suggest the possibility that they may be at risk from other medications as well. While our findings likewise reinforce the potential application of iPSC-derived cell types for drug toxicity testing, further studies to evaluate mechanisms by which specific agents induce toxicity in iPSC-hepatic cells versus primary human hepatocytes are likely to be important in understanding their predictive capacity (Sjogren et al., 2014).

In summary, our findings provide both global, genome-wide and focused, pathway-based views of disease-specific iPSCs as they differentiate in vitro into a target lineage responsible for AATD pathogenesis in patients. Our results provide comprehensive genetic and epigenetic databases for those interested in modeling human endoderm and liver development or liver disease onset. Future work can now focus on testing whether iPSC lines derived from each individual patient also have the capacity to model distinct patient-to-patient disease severity and clinical phenotypes known to occur among individuals with the same monogenic AATD mutation. Our results demonstrating drug responses in AATD iPSC-hepatic cells also raise the important question of whether individual patient iPSC lines also might predict in vivo individual therapeutic responses or toxicities to drugs, a hypothesis that will likely be tested in partnership with clinical drug trials in the years ahead.

## Experimental Procedures

### Production, Characterization, and Maintenance of PSCs

The recruitment of human subjects and all iPSC studies were approved by the Boston University Institutional Review Board (BUMC IRB H-27636). Human dermal fibroblast-derived iPSCs were generated and characterized to confirm pluripotency as previously described (Mills et al., 2013; Somers et al., 2010; Terrenoire et al., 2013). Where indicated, clones underwent additional evaluation by pluripotency array scores. iPSC and ESC lines were maintained in a 5% CO_2_ air environment in human iPSC media (Somers et al., 2010). iPSC clones underwent a minimum of 20 passages prior to additional experimentation. Additional details are described in the Supplemental Experimental Procedures.

### Directed Endodermal and Hepatic Differentiation of PSCs with Flow Cytometry Sorting/Analysis

Human PSCs were differentiated via the serial introduction of growth factors using previously described endodermal and hepatic differentiation protocols (Cheng et al., 2012). Additional details of the protocol and antibody staining procedures for flow cytometry and cell sorting are described in the Supplemental Experimental Procedures.

### ELISA

Secreted AAT and albumin were quantified in the supernatants of differentiated PSCs as previously described (Wilson et al., 2008, 2010). Human albumin was quantified using the Abcam Albumin Human ELISA kit per the manufacturer’s instructions.

### RNA Isolation and qPCR Analysis

Total RNA and miRNA were isolated from cells using an miRNeasy kit (QIAGEN) according to the manufacturer’s instructions. Human fetal liver control RNA was purchased from Clontech Laboratories. Real-time qPCR was performed in triplicate for all samples using either the SYBR Green or TaqMan systems as previously described (Mills et al., 2013; Somers et al., 2010). Additional details are described in the Supplemental Experimental Procedures.

### Gene Expression and DNA Methylation Microarray Analysis

Large and small RNAs were labeled with biotin before hybridization to Affymetrix GeneChip Human Gene 1.0 ST or miRNA 2.0 arrays and analyzed using previously published methods (Mills et al., 2013). miRNA 2.0 arrays were normalized to produce probeset-level expression values using the Affymetrix miRNA QC Tool (version 1.1.1.0), using default background detection, RMA global background correction, quantile normalization, and median polish summarization. Quantitative measurement of DNA methylation in study samples was achieved using Illumina’s Infinium HD Methylation Assay with HumanMethylation 450 BeadChip arrays. Additional details are described in the Supplemental Experimental Procedures.

### Statistical Analysis

PCA was performed using the *prcomp* R function with expression values normalized across all samples to a mean of zero and an SD of one. Differential gene expression was assessed using *limma* (version 3.14.4) by modeling expression as a linear function of cell type, time, and their interaction with *lmFit*, followed by adjustment with *eBayes*. Two-way ANOVA and moderated t tests were then performed using the *limma* functions *topTableF* and *topTable*, respectively. Correction for multiple hypothesis testing was accomplished using Benjamini-Hochberg FDR. All analyses were performed using the R environment for statistical computing.

### AAT Pulse-Chase Radiolabeling

iPSC line B-16 and its isogenic, zinc-finger nuclease (ZFN)-corrected daughter iPSC line B-16-C-2 were differentiated to the hepatic stage before labeling with ^35^S-Met/Cys. The kinetic of AAT post-translational intracellular processing and secretion was then assayed via pulse-chase labeling using previously described methods (Ordóñez et al., 2013). Additional details are described in the Supplemental Experimental Procedures.

### Quantification of Autophagic Flux

To quantify autophagosomes in differentiating iPSC-hepatic cells, T9 PiZZ or WT cells were transduced overnight with an LC3-GFP encoding lentivirus at an MOI of 12.5 in differentiation media containing polybrene (5 μg/ml). Cells were then differentiated until T16 before treatment with either CBZ (30 μM) or DMSO vehicle for 48 hr. At T18, live cells were imaged at 37°C, 5% CO2, using a Leica LSM710 confocal microscope (488-nm excitation), and GFP+ punctate autophagosomes were quantified by visual inspection in 50–60 cells per group before and after 4 hr of incubation with bafilomycin (LC Laboratories). LC3 and p62 were further quantified by western blot.

### Western Blot

PiZZ or WT iPSCs were differentiated to T16 or T18 before treatment with either CBZ, DMSO vehicle, or regular media as described for each experiment. Cell protein lysates were collected and separated in a 12% polyacrylamide gel before transfer onto a polyvinylidene fluoride (PVDF) membrane. Membranes were probed with antibodies against LC3 (Sigma-Aldrich), p62 (Abnova), B-actin (Sigma-Aldrich), KDEL (Grp78 and Grp94; Enzo Life Sciences), sXBP-1 (BioLegend), IκBα (Cell Signaling Technology), or GAPDH (Millipore). Signal was detected using goat anti-mouse or anti-rabbit HRP substrate (Bio-Rad) on a LAS-4000 luminescent image analyzer (Fuji) and Image J software was utilized to measure densitometry.

### PSC Drug Treatment and Analysis

CBZ (Sigma-Aldrich) was added at the concentrations indicated in the text to differentiation media. Cells, RNA, and supernatants were collected 48 hr later for analysis by flow cytometry, qPCR, ELISA, or western blot. To quantify the sensitivity of differentiating iPSC-hepatic cells to hepatotoxic drugs, acetaminophen, aflatoxin B, amiodarone, danazol, or puromycin was added to T20 differentiation media at the indicated concentrations. Cell viability was quantified after 4 hr (acetaminophen) or 4 days (aflatoxin B, amiodarone, danazol, and puromycin) via MTT assay. For 4-day drug exposures, media were changed once at the 48-hr time point.

## Author Contributions

D.N.K. and P.G. jointly conceived the study. D.N.K., P.G., A.A.W., L.Y., D.A.L., L.V., G.J.M., G.M., O.S.S., and M.I.R. contributed to experimental design. L.Y., A.A.W., M.L., C.-P.S., J.A.M., J.J., G.C.L., D.C.L., and A.O. performed experiments. L.Y., A.A.W., M.L., C.-P.S., S.S.S., A.H.L., J.N., A.C.G., F.-J.M., P.M., A.S., M.I.R., D.N.K., and P.G. analyzed and interpreted experimental data. D.N.K. and A.A.W. wrote and edited the paper.

## Accession Numbers

The accession number for the array data reported in this paper is GSE66078.

## Supplemental Information

Document S1. Supplemental Experimental Procedures, Figures S1–S5, and Tables S1–S3

Document S2. Article plus Supplemental Information
