# Supplementary material for: Emergence of a Stage-Dependent Human Liver Disease Signature with Directed Differentiation of Alpha-1 Antitrypsin-Deficient iPS Cells
Source: Stem Cell Reports. 2015 Apr 2;4(5):873–85. doi: 10.1016/j.stemcr.2015.02.021 (PMC4437473; doi:10.1016/j.stemcr.2015.02.021)
Supplement: Document S1. Supplemental Experimental Procedures, Figures S1–S5, and Tables S1–S3 [file mmc1.pdf]

Stem Cell Reports, Volume *4*

Supplemental Information

Emergence of a Stage-Dependent Human Liver Disease Signature with Directed Differentiation of Alpha-1 Antitrypsin-Deficient iPS Cells

Andrew A. Wilson, Lei Ying, Marc Liesa, Charis-Patricia Segeritz, Jason A. Mills, Steven S. Shen, Jyhchang Jean, Geordie C. Lonza, Derek C. Liberti, Alex H. Lang, Jean Nazaire, Adam C. Gower, Franz-Josef Müeller, Pankaj Mehta, Adriana Ordóñez, David A. Lomas, Ludovic Vallier, George J. Murphy, Gustavo Mostoslavsky, Avrum Spira, Orian S. Shirihai, Maria I. Ramirez, Paul Gadue, and Darrell N. Kotton

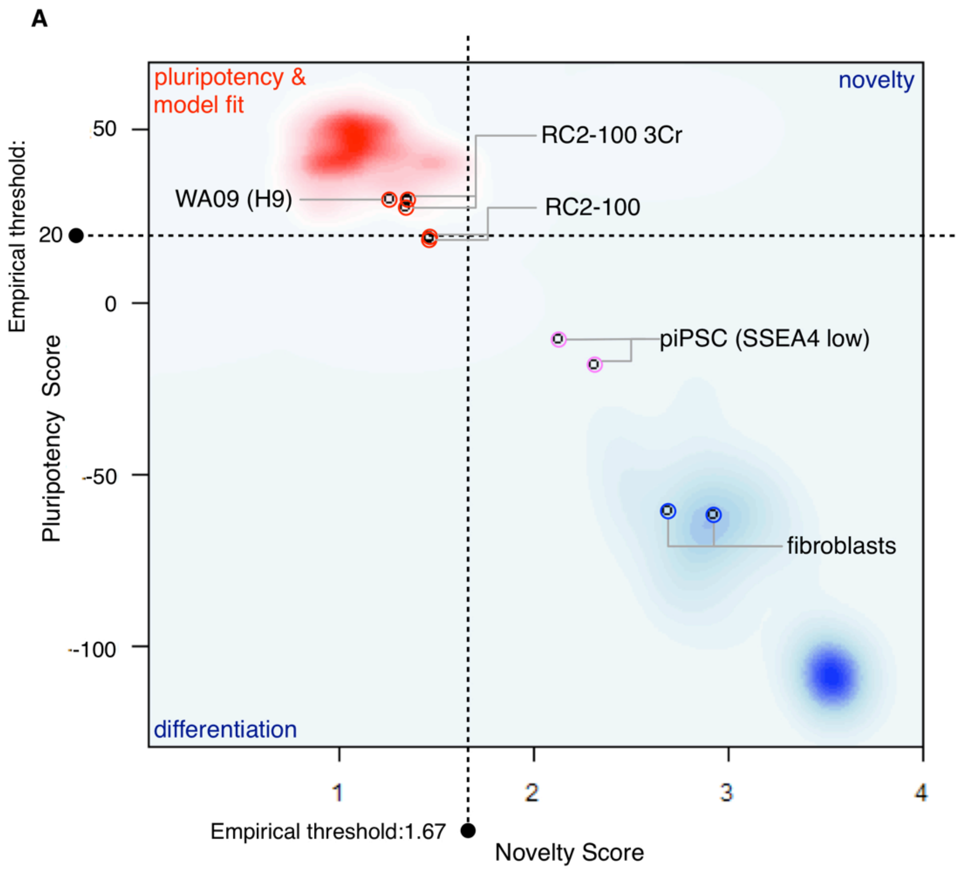

Figure S1 Pluritest scores based on global transcriptomic profiling of PiZZ iPSCs before and after Cre-mediated excision of the STEMCCA reprogramming vector. Pluritest characterization of the transcriptome of PiZZ iPSCs compared to benchmark pluripotent and partially reprogrammed clones demonstrates that PiZZ clones reprogrammed with the floxed STEMCCA polycistronic lentiviral reprogramming cassette have a high pluripotency score and cluster with ESCs as opposed to partially reprogrammed iPSCs. PluriTest (Muller et al. Nat Methods 2011 Mar 6) is a bioinformatic assay for accurate assessment of pluripotency. PluriTest analyzes the expression of a large number of pluripotency associated transcripts with a “Pluripotency Score” and tests for the conformity of a given sample with the global transcriptional profile typical for genetically and epigenetically normal human embryonic and induced pluripotent stem cells with a metric termed “Novelty Score”. The PluriTest assay provides a global statistical model for the genome-wide transcriptional landscape of human PSC. PluriTest results were plotted on the empirically defined density distribution for previously referenced pluripotent cells (red cloud) and somatic cells (blue cloud (Müller F-J, Schuldt BM, Williams R, Mason D, Altun G, Papapetrou EP, et al. A bioinformatic assay for pluripotency in human cells. Nat Methods. 2011 Mar 6)

Figure S2

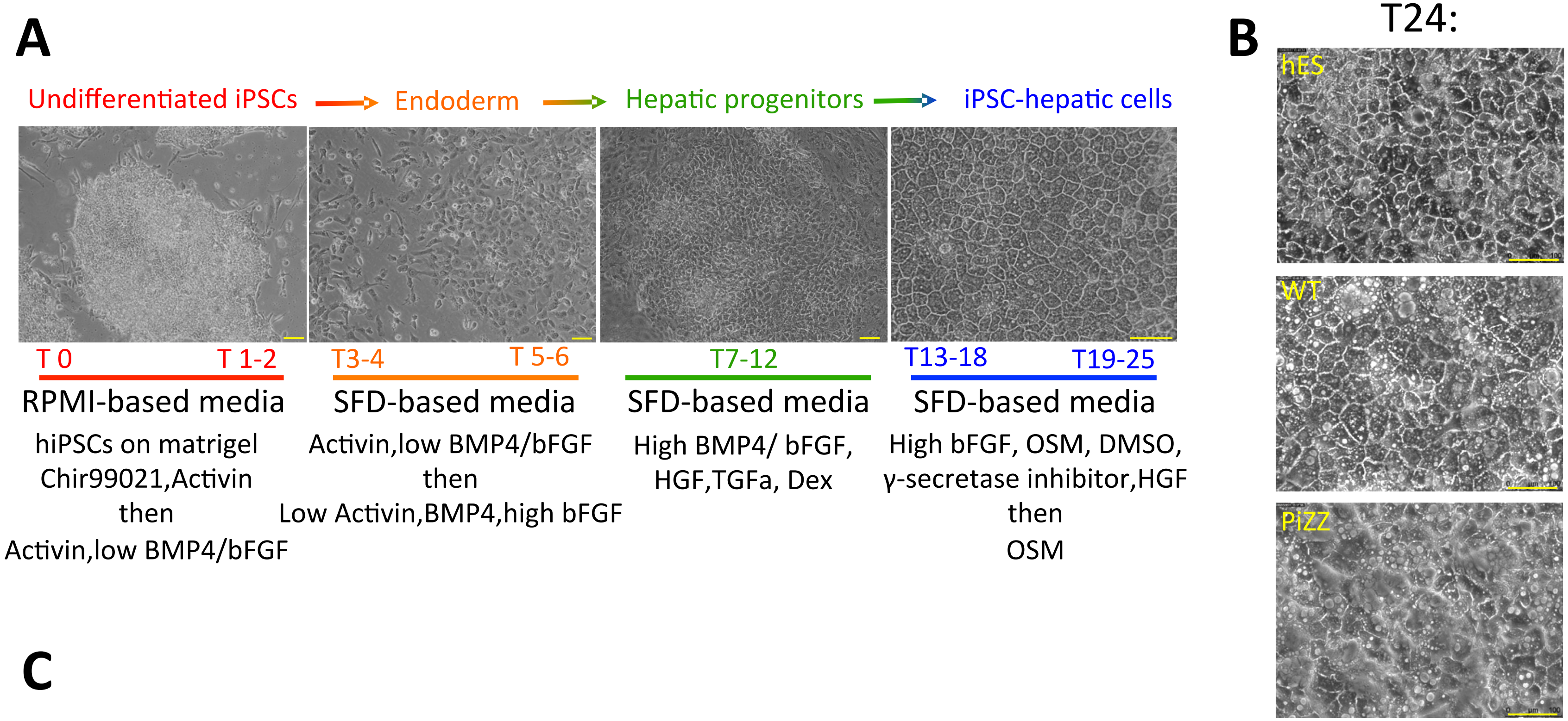

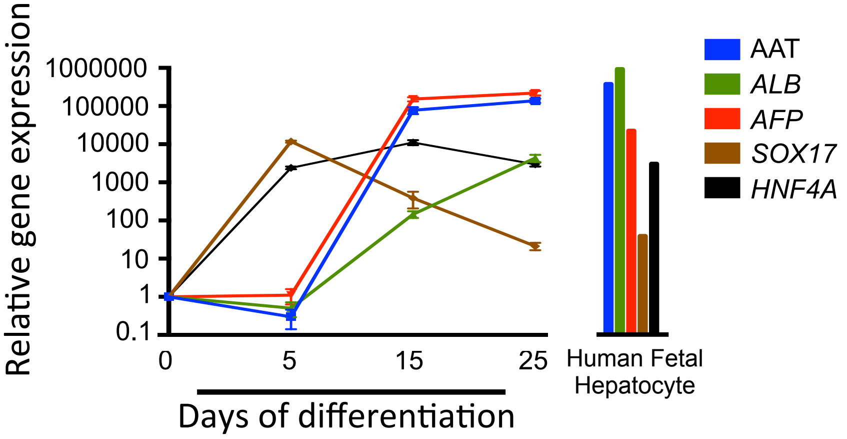

Figure S2 iPSC directed differentiation protocol. (**A**) Undifferentiated iPSCs are passaged off of MEFs onto gelatin-coated dishes 2 days before starting differentiation. At T0, endodermal differentiation is initiated by 1 day of media supplementation with Chir99021 and Activin A. Media is changed daily through T6 and every other day for the remainder of the protocol. The base media is changed to SFD beginning at T3. Scale bars: 100 uM. (**B**) At T24, cells exhibit a characteristic polygonal hepatocyte-like morphology. Scale bars: 100 uM.(**C**) qPCR demonstrates gene expression levels at key developmental time points during differentiation compared to T0 iPSCs. *Sox17*and *HNF4* expression levels are increased at the endodermal stage while expression of hepatic genes, such as AAT, albumin, and *AFP* increases later during differentiation. Gene expression levels at hepatic stage are similar to those seen in human fetal hepatocytes. n = 3 biological replicates. Data are represented as mean +/- SEM.

Figure S3

Figure S3 Flow cytometric characterization of ESC, WT iPSC, and PiZZ iPSC lines at key developmental timepoints. All 9 pluripotent stem cell lines were analyzed at the undifferentiated stage (T0), definitive endoderm stage (T5), or during hepatic specification and maturation (T7-T24) with stage-specific markers at each time point. Cells were sorted using the indicated gates for TRA-1-81+/SSEA3+ (T0) or C-kit+/CXCR4+ (T5) cells. The AAT/FOXA1 protein expression kinetic exhibits some line to line variation but all lines achieve high levels of coexpression by T24.

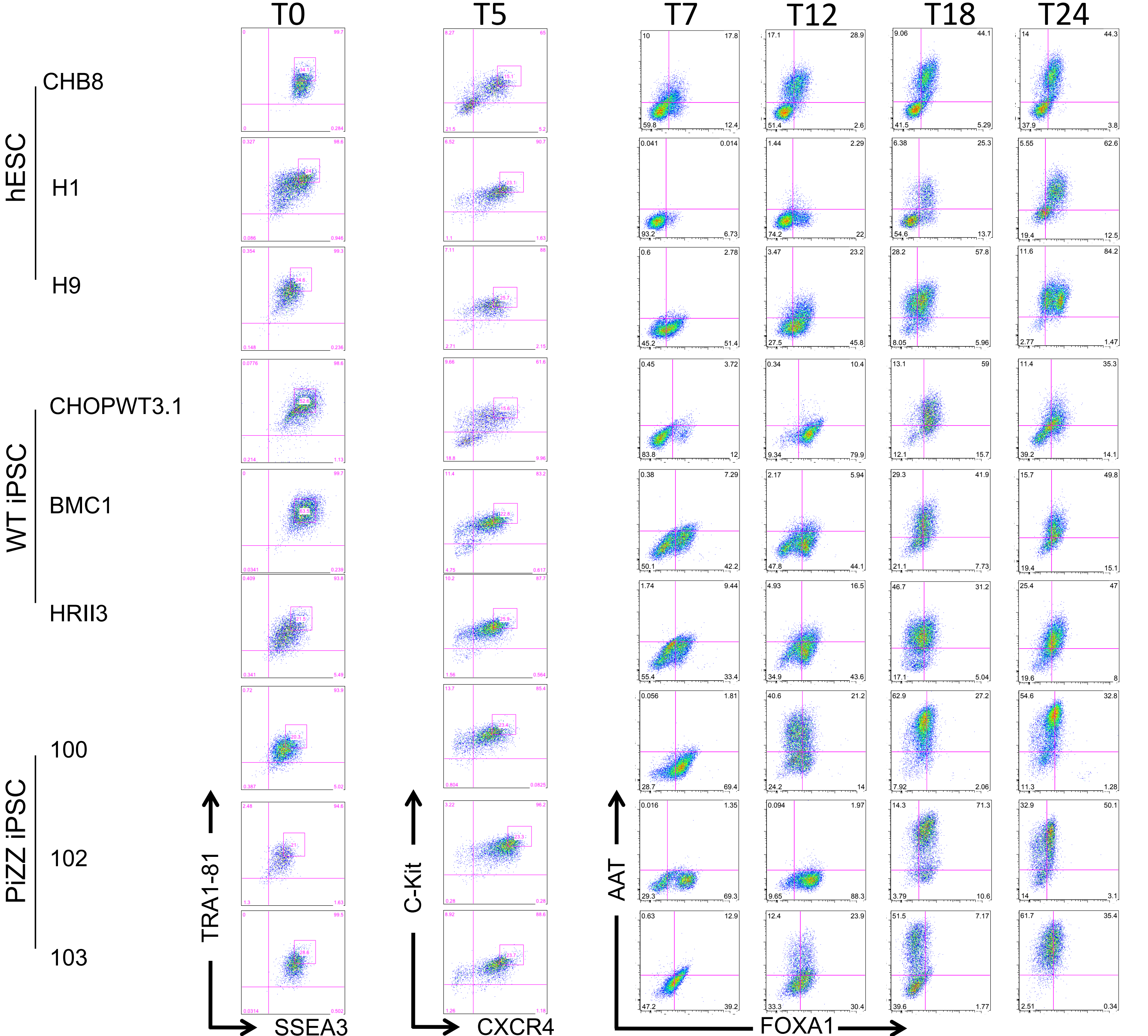

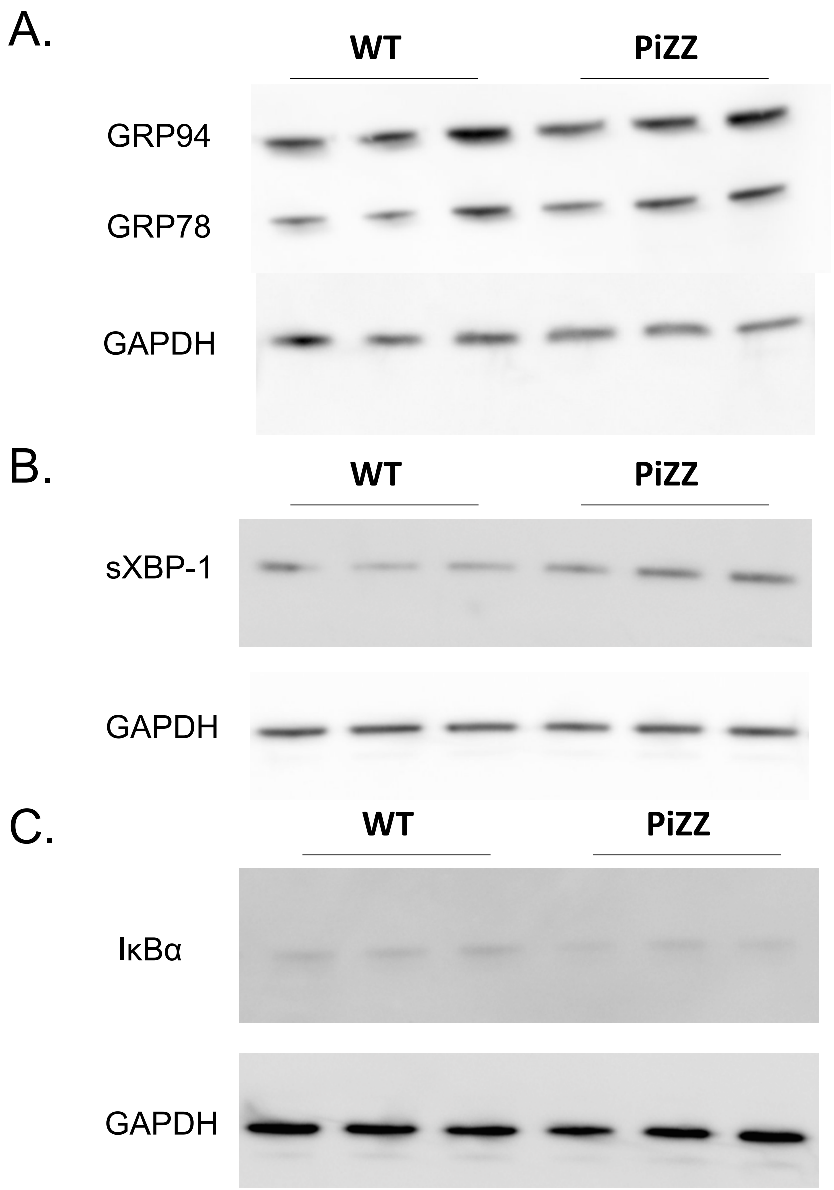

Figure S4 Differential expression of UPR-associated proteins in PiZZ iPSC-hepatic cells. Cell lysates were prepared from triplicate wells of differentiating cells at T16 or T18. Western blots were then performed using primary antibodies against GRP78, GRP94 **(A)**, the mature spliced form of XBP-1 (**B**), and the NF-κB inhibitory protein IκBα. In each case, membranes were strippled and re-probed for GAPDH to control for protein loading.

Figure S5

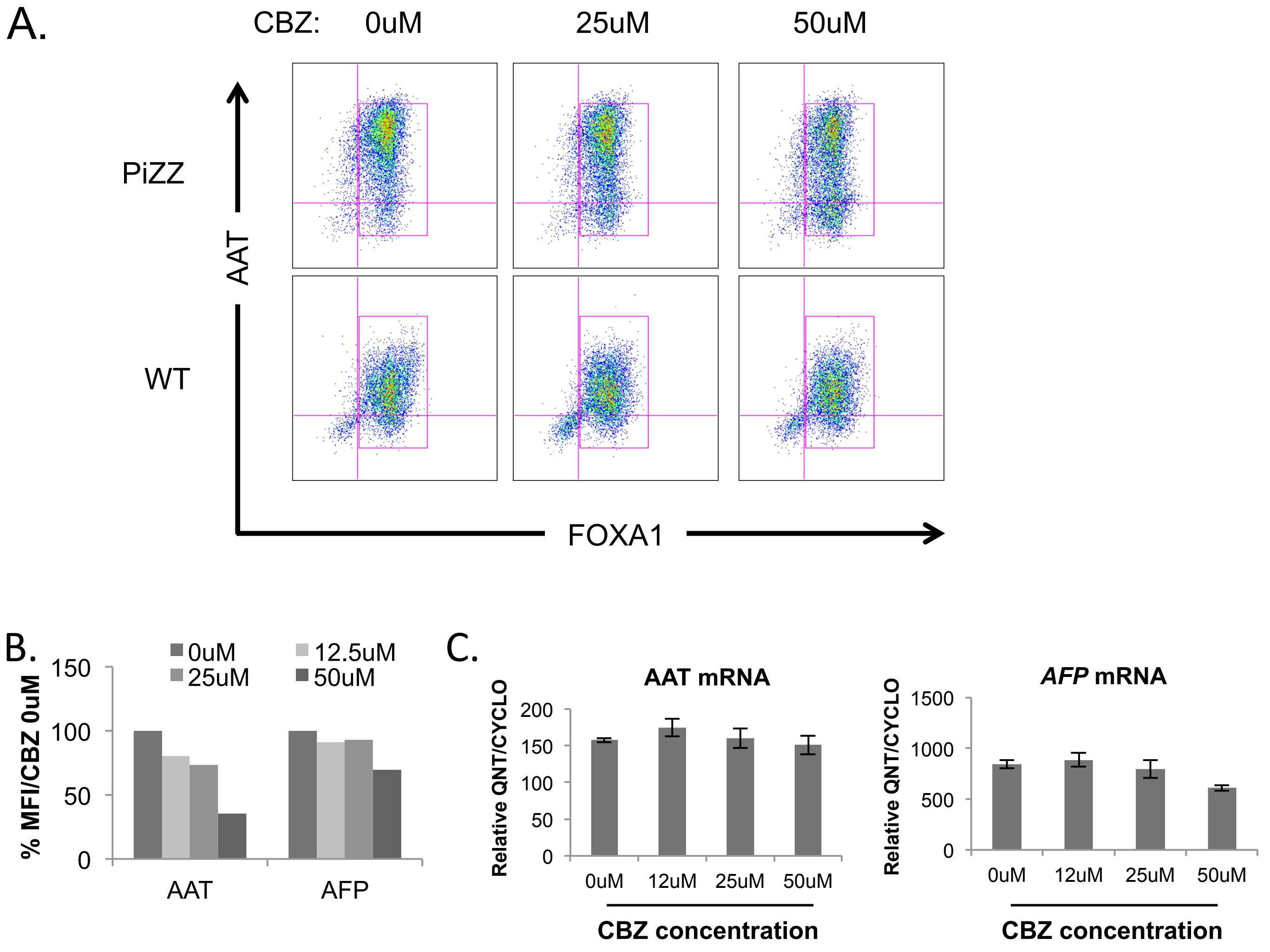

Figure S5 CBZ treatment decreases intracellular AAT in PiZZ iPSC-hepatic cells. (**A**) Flow cytometry demonstrates that the mean fluorescence intensity (MFI) of intracellular AAT antibody staining decreases with CBZ treatment in PiZZ, but not WT, iPSC-hepatic cells. (**B**) MFI of intracellular AAT antibody staining decreases with increasing CBZ concentration in PiZZ cells. This effect is not seen with intracellular AFP. (**C**) CBZ treatment does not affect levels of AAT or *AFP* transcription. n = 3 independent experiments. Data are represented as mean +/- SEM.

Table S1

TABLE: DISEASE SIGNATURE: 2-WAY ANOVA INTERACTION EFFECT; FDR<0.25				
Gene.Symb	adj.P.Val (FDR)	logFC Alpha vs iPSC T24	P.Value	mRNA.Accession
DNAH5	0.001387322	1.889999333	4.75E-08	NM_001369
CASP4	0.014884331	1.907756	1.46E-06	NM_033306
CFH	0.014884331	2.682600333	1.88E-06	NM_000186
HAVCR2	0.014884331	2.277453333	2.04E-06	NM_032782
ERAP2	0.029128034	2.761159	4.99E-06	NM_022350
KCNJ6	0.04186302	1.373596	8.60E-06	NM_002240
---	0.109360946	1.046387333	2.62E-05	ENST00000474296
WFDC10B	0.116203146	1.470892667	3.58E-05	NM_172006
GRM4	0.116203146	0.798611667	3.61E-05	NM_000841
---	0.116203146	-0.723990667	3.98E-05	NM_033534.1
TFEC	0.14674925	-1.188121667	5.53E-05	NM_012252
---	0.156395992	0.467866333	6.82E-05	ENST00000517114
CA10	0.156395992	0.965961333	6.96E-05	NM_001082533
CGA	0.172961529	-1.958114	8.29E-05	NM_000735
CCL20	0.181722404	2.338906667	9.92E-05	NM_004591
---	0.181722404	0.719536333	0.0001026	ENST00000482613
WIF1	0.181722404	-1.585783	0.0001233	NM_007191
DLG3	0.181722404	-0.57688	0.0001283	NM_021120
---	0.181722404	0.486010667	0.0001304	ENST00000515976
IL4R	0.181722404	0.987948667	0.0001421	NM_000418
RNF133	0.181722404	1.055310333	0.0001536	NM_139175
LOC100133	0.181722404	1.326300667	0.0001538	AY358688
LSM14B	0.181722404	-0.522890667	0.0001562	NM_144703
RNF148	0.181722404	1.341163333	0.0001578	NM_198085
SERPINA3	0.181722404	1.056103667	0.000164	NM_001085
---	0.181722404	0.479801	0.0001648	BC031827
TUBB4	0.181722404	-1.582957333	0.0001749	NM_006087
---	0.181722404	1.277718	0.0001762	ENST00000472474
SQRDL	0.181722404	1.140718333	0.0001829	NM_021199
---	0.181722404	0.622869	0.0002112	ENST00000463780
---	0.181722404	0.622869	0.0002112	ENST00000463780
---	0.181722404	1.922892333	0.0002119	ENST00000517171
DPPA3	0.181722404	-1.757035	0.000214	NM_199286
ROCK1P1	0.181722404	-1.561588333	0.0002168	NR_033770
---	0.181722404	0.604009667	0.0002178	ENST00000517281
---	0.187512805	1.229577333	0.0002405	---
---	0.187512805	0.834795	0.0002476	ENST00000391266
AMY2A	0.187512805	-0.95882	0.0002559	NM_000699
FOXO4	0.187512805	-1.266531333	0.0002681	NM_005938
---	0.187512805	1.068346667	0.000274	---
AMY1A	0.187512805	-0.854025	0.0002829	NM_004038
AMY1A	0.187512805	-0.854025	0.0002829	NM_004038
AMY1A	0.187512805	-0.854025	0.0002829	NM_004038
C1R	0.187512805	0.820498333	0.0002853	NM_001733
ZNF70	0.187512805	-0.585746667	0.0002933	NM_021916
SCN3A	0.187512805	1.276423333	0.0002983	NM_006922
SLAMF8	0.187512805	-0.866773333	0.000314	NM_020125
CFHR1	0.187512805	2.519885667	0.0003146	NM_002113
HSD3BP4	0.187512805	1.100559	0.0003147	NR_033781
ERVFRDE1	0.194279346	-0.507456333	0.0003375	NM_207582
APOL1	0.194279346	2.046024333	0.0003429	NM_145343
OVCH2	0.194279346	2.153060333	0.0003483	NM_198185
GEM	0.194279346	-1.298775667	0.0003621	NM_005261
CCDC151	0.194279346	0.563474667	0.0003631	NM_145045
VNN3	0.194279346	0.810899667	0.0003766	NR_028290
FURIN	0.194279346	1.012876	0.0003783	NM_002569
---	0.194279346	0.544883667	0.0003793	ENST00000470887
STYK1	0.199898667	1.076626333	0.0003971	NM_018423
SPAG5	0.209295324	-1.853380333	0.0004349	NM_006461
LGALS17A	0.209295324	0.614917667	0.0004358	NR_034156
C7orf34	0.209295324	0.572407	0.0004373	NM_178829
---	0.212105936	0.479675333	0.0004525	ENST00000516879
C20orf151	0.212105936	0.511883667	0.0004577	NM_080833
SLC22A12	0.2159028	0.497858667	0.0004732	NM_144585
SOD2	0.219531946	0.871593667	0.0004905	NM_001024465
COMMD9	0.219531946	0.520874333	0.0004989	NM_014186
---	0.219531946	-0.536388333	0.0005112	ENST00000410424
---	0.219531946	0.608298333	0.0005133	ENST00000469282

TABLE: DISEASE SIGNATURE: 2-WAY ANOVA INTERACTION EFFECT; FDR<0.25				
Gene.Symb	adj.P.Val (FDR)	logFC Alpha vs iPSC T24	P.Value	mRNA.Accession
PRDM16	0.219531946	0.516072667	0.0005188	NM_022114
---	0.221976556	0.588018667	0.0005322	ENST00000490342
---	0.223906356	-0.955998667	0.0005601	ENST00000516933
---	0.223906356	-2.844913333	0.0005653	GENSCAN00000020996
SLC4A8	0.223906356	-2.024568333	0.000566	NM_001039960
---	0.223906356	0.616375333	0.0005711	ENST00000462689
DNAH14	0.223906356	0.708480333	0.0005866	NM_001373
RBMXL3	0.223906356	0.536356333	0.0005871	NM_001145346
LSM14B	0.223906356	-0.608586333	0.0005905	NM_144703
C1QTNF1	0.225500686	1.047790667	0.0006112	NM_030968
SIRPA	0.225500686	0.853482667	0.0006147	NM_001040022
---	0.225500686	0.502454	0.0006216	ENST00000364309
---	0.225500686	-0.706863	0.0006256	ENST00000466549
GDPD2	0.227361845	-1.2859	0.0006473	NM_001171192
VSIG8	0.227361845	0.559231667	0.0006487	NM_001013661
---	0.227361845	0.585405667	0.0006541	ENST00000469816
SCARA3	0.235795427	-1.048902	0.0006946	NM_016240
VNN2	0.235795427	0.542387667	0.0007088	NM_004665
---	0.235795427	0.776246	0.0007091	AB062477
---	0.235795427	-0.806129333	0.0007178	ENST00000485415
ANGPTL4	0.235795427	1.398495333	0.0007187	NM_139314
---	0.237970592	0.681261333	0.0007335	ENST00000384144
---	0.238118688	0.469489	0.0007421	ENST00000517020
RIMBP3	0.239089881	0.417748	0.0007533	NM_015672
BIN1	0.249495739	-1.057385667	0.0007963	NM_139343
---	0.249495739	1.000222667	0.00081	ENST00000411306
MBOAT4	0.249495739	0.486972	0.0008414	NM_001100916
---	0.249495739	0.759812	0.0008696	ENST00000459212
CENPI	0.249495739	-1.301527333	0.0008794	NM_006733
PDZK1IP1	0.249495739	1.262729667	0.0008899	NM_005764
FLJ43763	0.249495739	0.592882333	0.0008916	ENST00000381078
AZGP1	0.249495739	0.897379	0.0008922	NM_001185
TNFSF10	0.249495739	1.447382	0.0008922	NM_003810
BHLHE41	0.249495739	-0.876479667	0.0009045	NM_030762
FAM49A	0.249495739	-1.123131333	0.0009089	NM_030797
ZYG11A	0.249495739	-1.441144333	0.0009157	NM_001004339
LRG1	0.249495739	0.693725667	0.0009164	NM_052972
COX7A1	0.249495739	-0.812038667	0.0009419	NM_001864
C2orf83	0.249495739	0.599935	0.0009563	NM_020161
BATF	0.249495739	0.612902	0.0009624	NM_006399
PSG5	0.249495739	-2.975424333	0.0009645	NM_002781
---	0.249495739	0.549399667	0.0009702	ENST00000484467
EHF	0.249495739	1.758787667	0.0009958	NM_012153
KY	0.249495739	0.474578333	0.0010185	NM_178554
---	0.249495739	0.530830667	0.0010269	ENST00000505282
KIF22	0.249495739	-1.472044	0.0010341	NM_007317
GALNT5	0.249495739	1.475392333	0.0010432	NM_014568
TGM2	0.249495739	0.690833	0.001051	NM_004613
KIF22	0.249495739	-1.478056333	0.0010542	NM_007317
---	0.249495739	0.546366667	0.0010566	---
OR5A2	0.249495739	0.772161667	0.0010634	NM_001001954
---	0.249495739	-1.686442667	0.0010741	NM_199286.2
CPD	0.249495739	0.589138333	0.0010802	NM_001304
ABHD10	0.249495739	-0.79874	0.0010842	NM_018394
GCM1	0.249495739	-0.595818667	0.0010876	NM_003643
RIMBP3	0.249495739	0.441950667	0.0010905	NM_015672
---	0.249495739	0.506613	0.0010976	ENST00000516864
PLEK2	0.249495739	1.571681	0.0011055	NM_016445
NUSAP1	0.249495739	-1.449468333	0.0011097	NM_016359
---	0.249495739	1.235091667	0.0011099	ENST00000365415
---	0.249495739	0.429498	0.0011166	ENST00000486780
---	0.249495739	0.977655667	0.0011282	AK097085
FLJ36840	0.249495739	0.942958333	0.0011298	AK094159
CDCA7	0.249495739	-1.021459667	0.0011363	NM_031942
---	0.249495739	0.640481667	0.0011393	ENST00000364488
---	0.249495739	0.901475667	0.0011515	ENST00000493687
TNFAIP6	0.249495739	-1.582938	0.0011536	NM_007115

Table S1: PiZZ disease-specific transcriptomic signature. Post-hoc moderated t testing of the “interaction effect” identified 135 differentially expressed transcripts at T24 in PiZZ diseased versus normal iPSC-hepatic cells.

PiZZ:ESC/WT	miRNA T0	miRNA T5	miRNA T24	methylation T0	methylation T5	methylation T24
**Up**	0	0	0	20	37	150
**Down**	0	0	0	3	39	45
**Unchanged**	638	638	638	322311	322258	322139

Table S2: Summary of changes in miRNA expression and CpG methylation between diseased and normal pluripotent stem cells at key developmental stages. PiZZ iPSCs are compared to WT iPSCs and ESCs. FDR adjusted q values are <0.001 (miRNA), and <0.05 (methylation).

		Gene Expression			Methylation			Correlation	
GeneSymbol	GeneName	Fold Change	P Value	FDR-adjusted P	ID Methylation Site	Fold Change	P Value	Pearson Correlation	P Value
BATF	basic leucine zipper transcription factor, ATF-like	1.53	9.62E-04	0.249	cg09937039	-1.81	6.91E-04	-0.91	1.3E-02
BATF	basic leucine zipper transcription factor, ATF-like	1.53	9.62E-04	0.249	cg14266927	-1.93	2.81E-03	-0.86	2.9E-02
BATF	basic leucine zipper transcription factor, ATF-like	1.53	9.62E-04	0.249	cg14424070	-3.38	5.55E-03	-0.89	1.7E-02
BATF	basic leucine zipper transcription factor, ATF-like	1.53	9.62E-04	0.249	cg15645309	-1.82	1.48E-02	-0.86	2.9E-02
BATF	basic leucine zipper transcription factor, ATF-like	1.53	9.62E-04	0.249	cg21531300	-2.85	3.60E-03	-0.91	1.1E-02
BATF	basic leucine zipper transcription factor, ATF-like	1.53	9.62E-04	0.249	cg22995449	-2.02	1.57E-03	-0.83	3.9E-02
BATF	basic leucine zipper transcription factor, ATF-like	1.53	9.62E-04	0.249	cg23723793	-1.92	4.03E-03	-0.91	1.2E-02
C1QTNF1	C1q and tumor necrosis factor related protein 1	2.07	6.11E-04	0.226	cg14020904	-1.33	1.36E-02	-0.92	9.5E-03
C1QTNF1	C1q and tumor necrosis factor related protein 1	2.07	6.11E-04	0.226	cg17758081	-1.29	7.56E-03	-0.85	3.0E-02
C1R	complement component 1, r subcomponent	1.77	2.85E-04	0.188	cg08799922	-1.43	2.02E-02	-0.85	3.1E-02
CFHR1	complement factor H-related 1	5.74	3.15E-04	0.188	cg12687463	-1.56	2.73E-03	-0.83	4.3E-02
GRM4	glutamate receptor, metabotropic 4	1.74	3.61E-05	0.116	cg08969344	-1.20	2.48E-02	-0.89	1.7E-02
HAVCR2	hepatitis A virus cellular receptor 2	4.85	2.04E-06	0.015	cg19110684	-1.41	5.11E-03	-0.87	2.4E-02
KCNJ6	potassium inwardly-rectifying channel, subfamily J, member 6	2.59	8.60E-06	0.042	cg03531951	-1.30	1.40E-02	-0.87	2.6E-02
LRG1	leucine-rich alpha-2-glycoprotein 1	1.62	9.16E-04	0.249	cg03882382	-1.84	1.52E-03	-0.81	4.8E-02
LRG1	leucine-rich alpha-2-glycoprotein 1	1.62	9.16E-04	0.249	cg17272620	-1.39	7.14E-03	-0.96	2.1E-03
LRG1	leucine-rich alpha-2-glycoprotein 1	1.62	9.16E-04	0.249	cg22375763	-1.48	1.67E-02	-0.89	1.6E-02
MBOAT4	membrane bound O-acyltransferase domain containing 4	1.40	8.41E-04	0.249	cg00940560	-1.51	3.53E-02	-0.85	3.2E-02
MBOAT4	membrane bound O-acyltransferase domain containing 4	1.40	8.41E-04	0.249	cg21058822	-2.60	2.71E-02	-0.94	5.2E-03
PRDM16	PR domain containing 16	1.43	5.19E-04	0.22	cg04873098	-1.40	3.80E-02	-0.96	2.6E-03
PRDM16	PR domain containing 16	1.43	5.19E-04	0.22	cg15519786	-2.01	3.18E-02	-0.83	4.0E-02
PRDM16	PR domain containing 16	1.43	5.19E-04	0.22	cg17220278	-1.19	4.14E-02	-0.89	1.6E-02
PRDM16	PR domain containing 16	1.43	5.19E-04	0.22	cg21789941	-1.70	2.62E-03	-0.85	3.0E-02
SLC22A12	solute carrier family 22 (organic anion/urate transporter), member 12	1.41	4.73E-04	0.216	cg07220939	-1.59	3.87E-03	-0.95	3.4E-03
TNFSF10	tumor necrosis factor (ligand) superfamily, member 10	2.73	8.92E-04	0.249	cg08144586	-1.28	8.60E-03	-0.87	2.6E-02
VSIG8	V-set and immunoglobulin domain containing 8	1.47	6.49E-04	0.227	cg21574855	-1.46	5.12E-03	-0.85	3.3E-02

Table S3 Anticorrelation of gene expression and CpG methylation in genes comprising the PiZZ disease signature. 13 of the 135 genes comprising the signature exhibit differential methylation at the indicated sites that is decreased in PiZZ iPSC-hepatic cells in comparison with WT and anticorrelated with gene expression (increased in PiZZ iPSC-hepatic cells compared to WT).

Supplemental Experimental Procedures:

Production of iPSCs from human fibroblasts. Normal human dermal fibroblast-derived iPSCs were generated by reprogramming with a single-integrated excisable copy of the floxed hSTEMCCA lentiviral reprogramming vector(Somers et al., 2010) followed by excision with transient Cre recombinase-exposure. These three lines generated from three separate individuals have been previously characterized, published, and named (BMC1, CHOPWT3.1, and HRII-3(Mills et al., 2013; Somers et al., 2010; Terrenoire et al., 2012). PiZZ fibroblasts, isolated by 6-mm full thickness arm skin punch biopsy from volunteer subjects with AAT deficiency as previously described(Somers et al., 2010), were grown in DMEM with 10% FBS. The recruitment of human subjects and all iPSC studies were approved by the Boston University Institutional Review Board (BUMC IRB H-27636). For reprogramming, fibroblasts were transduced with the humanized hSTEMCCA lentiviral reprogramming vector(Mills et al., 2013; Somers et al., 2010). iPSC colonies were mechanically isolated 30 days after transduction and expanded on MEF feeders in human iPSC media. Integrated hSTEMCCA copy number was assessed by Southern blot of gDNA extracts as previously published(Somers et al., 2010), and only iPSC clones with single copy hSTEMCCA integrations were selected for vector excision and further study.

iPSC expansion and characterization. iPSC clones were passaged and expanded in hiPSC media for a minimum of 20 passages prior to additional experimentation. Clones were characterized for expression of genes associated with pluripotency and teratoma assays performed as previously published to confirm functional pluripotency(Somers et al., 2010). Where indicated in the text and supplement, global transcriptomes were scored by Pluritest assay to obtain pluripotency array scores(Müller et al., 2011).

Cre-mediated hSTEMCCA excision. The single copy hSTEMCCA lentiviral cassette was removed from each iPSC clone via transient transfection of pHAGE2-Cre-IRES-PuroR plasmid DNA using Hela Monster transfection reagent (Mirus, Madison, WI, www.mirusbio.com) according to the manufacturer's instructions(Somers et al., 2010). Approximately 11-14 days later, colonies were picked and gDNA from each subclone screened for vector excision by PCR using the following primers and conditions: cMYC F5'-GGA ACT CTT GTG CGT AAG TCG ATA G-3'; WPRE R5'-GGA GGC GGC CCA AAG GGA GAT CCG-3'; 95° C for 3 minutes; followed by 33 cycles of 94°C for 30 seconds, 60°C for 30 seconds, and 72°C for 1 minute; followed by a single cycle of 72°C for 5 minutes. Vector excision was then confirmed by Southern blot using BamHI digested gDNA probed for the WPRE element as previously published(Somers et al., 2010).

Cell culture and maintenance of PSCs. iPSC and ESC lines were maintained in "hiPSC Media" composed of DMEM/F12 (Sigma-Aldrich) with 20% KnockOut Serum Replacement (Invitrogen), 1mM nonanimal L-glutamine (Sigma-Aldrich), 0.1mM B-mercaptoethanol, and 10 ng/ml FGF2 (R&D Systems) on 0.1% gelatin (Sigma-Aldrich) coated plates preseeded with mitomycin C-inactivated or irradiated mouse embryonic fibroblast (MEF) feeder cells. Cells were maintained in a 5% CO_2 air environment.

Directed Endodermal and Hepatic Differentiation of PSCs. Human PSCs were differentiated using previously described protocols(Cheng et al., 2012). For endodermal differentiation, cells were passed onto matrigel-coated dishes at 80% confluency. On the following day, designated "T0", differentiation was induced by culture in media containing growth factors listed below. From T0-T6, differentiation media included 2mM l-glutamine, and $4.5x10^{-4}$ M monothioglycerol (MTG). Cells were grown in T0 media, consisting of RPMI-based serum-free medium with Chir

99021 (2ug/ml) and Activin A (100 ng/ml), for one day. On days “T1-2”, medium was changed to RPMI with BMP4 (0.5 ng/ml), FGF2 (10ng/ml), Activin A (100 ng/ml), and VEGF (10 ng/ml). On days “T3-4”, cells were cultured in SFD media(Gouon-Evans et al., 2006) with BMP4 (0.5 ng/ml), FGF2 (10ng/ml), Activin A (100 ng/ml), and VEGF (10 ng/ml). For hepatic differentiation, PSCs were differentiated as monolayer cultures as outlined above to generate definitive endoderm and then further differentiated for an additional 3 weeks in SFD-based media with ascorbic acid (50mcg/ml), monothioglycerol ($4.5x10^{-4}$ M), and the following supplements: T7-12: BMP4 (50 ng/ml), FGF2 (10 ng/ml), VEGF (10 ng/ml), EGF (10ng/ml), TGFa (20 ng/ml), HGF (100 ng/ml), and 0.1 uM Dexamethasone; T13-18: FGF2 (10 ng/ml), VEGF (10 ng/ml), EGF (10 ng/ml), HGF (100 ng/ml), Oncostatin M (20 ng/ml), Vitamin K (6 ug/ml), 1.5 uM gamma secretase inhibitor, 0.1 uM Dexamethasone, and 1% DMSO; T19-24: HGF (100 ng/ml), Oncostatin M (20 ng/ml), Vitamin K (6 ug/ml), and 0.1 uM Dexamethasone. Differentiating human PSCs were maintained in a 5% CO_2, 5% O_2, 90% N_2 environment.

Flow cytometry and cell sorting. PSCs were stained with antibodies for the following cell surface antigens: CD117-APC (Invitrogen, #11705), CXCR4-PE (Invitrogen, #MHCXCR404), TRA-1-81-Alexa Fluor 647 (Biolegend, #330706), and SSEA3-AlexaFlour488 (Biolegend, #330306). For staining of intracellular antigens, cells were fixed in 1.6% paraformaldehyde for 20 minutes at 37°C and then permeabilized in saponin buffer (Biolegend). Fixed cells were stained with antibodies against human FOXA1 (Santa Cruz, #sc-101058), AAT (Santa Cruz, #sc-59438), or AFP (R&D, # MAB1368) followed by goat anti-mouse IgG2a-DyLight488 (Jackson ImmunoResearch, # 115-485-206), IgG1-DyLight649 (Jackson ImmunoResearch, #705-496-147) or IgG-AlexaFluor647(Jackson ImmunoResearch, # 115-605-205) antibodies. For all flow cytometry experiments, gating was based on isotype-stained controls. Staining was quantified using a FACSCantos II flow cytometer (BD Biosciences) and analyzed with FlowJo software (Tree Star Inc). To obtain cell pure populations for analysis, T0 and T5 cells were first sorted by flow sorting SSEA3/TRA-1-81 double positive (T0) or CD117/CXCR4 double positive (T5) cells on a FACS Aria II (Becton Dickenson) sorter. Cells were resuspended in Qiazol Lysis Reagent (Qiagen) and snap frozen before storage at -80°C.

Gene expression microarray analysis. Biotin labeling of large RNA was performed using the Ambion WT Expression Kit (Life Technologies, Grand Island, NY) according to the manufacturer's protocol, followed by the GeneChip WT Terminal Labeling and Controls Kit (Affymetrix, Santa Clara, CA). Biotin labeling of small RNA was performed using the Affymetrix FlashTag Biotin HSR RNA Labeling Kit according to the manufacturer's protocol. Labeled, fragmented DNA was then hybridized to Affymetrix GeneChip Human Gene 1.0 ST or miRNA 2.0 arrays, and after staining, microarrays were immediately scanned using an Affymetrix GeneArray Scanner 3000 7G Plus. Human Gene 1.0 ST arrays were normalized to produce gene-level expression values using the implementation of the Robust Multiarray Average (RMA) in the Affymetrix Expression Console software package. miRNA 2.0 arrays were normalized to produce probeset-level expression values using the Affymetrix miRNA QC Tool (version 1.1.1.0), using default background detection, RMA global background correction, quantile normalization, and median polish summarization. Analysis was limited to the 1,105 human microRNAs interrogated by the array. Human Gene 1.0 ST and miRNA 2.0 files have been desposited in the Gene Expression Omnibus (GEO accession number GSE66078).

DNA methylation microarray analysis. Quantitative measurement of DNA methylation in study samples was achieved using Illumina’s Infinium HD Methylation Assay with HumanMethylation 450 BeadChip arrays. Briefly, 500ng gDNA extracted from hESCs, iPSCs derived from normal individuals, and iPSCs from alpha-1 antitrypsin deficient patients underwent bisulfite conversion of unmethylated cytosine bases to uracil. Bisulfite converted DNA was then amplified and

purified prior to overnight hybridization to BeadChip arrays. Next day staining of hybridized arrays produced methylation-dependent differential fluorescence that was detected via an Illumina iScan array scanner. Arrays were visualized and processed using the GenomeStudio software package, which produced IDAT files that were read into a MethyLumiSet using the *methylumIDAT* function in the *methylumi* R package (version 2.4.0). This object was then coerced to a MethyLumiM object, quantile-normalized by sequentially applying the *lumiMethyC* and *lumiMethyN* functions from the *lumi* R package (version 2.10.0), and annotated using the *IlluminaHumanMethylation450k.db* R package (version 1.4.7). Differential methylation between wildtype and PiZZ iPSCs at stage T24 was assessed using the *limma* R package (version 3.14.4) by modeling M values as a linear function of genotype with *lmFit*, followed by empirical Bayesian adjustment with *eBayes* and a moderated *t* test performed using *topTable*. Methylation and expression probesets were matched on gene symbols in order to compute Pearson correlation coefficients between M values and log2 (expression) values from iPSCs at stage T24. All methylation analyses were performed using the R environment for statistical computing (version 2.15.1). Illumina 450K files have been desposited in the Gene Expression Omnibus (GEO accession number GSE66078).

*RNA isolation and quantitative PCR analysis.*Total RNA and miRNA were isolated from cells using an miRNeasy kit (Qiagen) with the optional column RNAse-free DNase treatment, according to the manufacturer's instructions. 200 nanograms to one microgram of RNA was reverse transcribed into cDNA using random hexamers with Superscript III Reverse Transcriptase (Invitrogen). Real-time, quantitative PCR (qPCR) was performed in triplicate for all samples using either SYBR Green QPCR master mix with the Light Cycler 480II qPCR System (Roche, Indianapolis, IN, www.roche.com) or TaqMan primers and master mix with a StepOne Real Time PCR system (Applied Biosystems, Carlsbad, CA, www.lifetechnologies.com). For SYBR Green qPCR, a 10-fold gDNA dilution series ranging from 0.1 to10 ng per reaction was used to evaluate the efficiency of the PCR and calculate the copy number of each gene relative to the housekeeping gene Cyclophilin. Calculated expression levels for each indicated gene were then reported as number of molecules of RNA for that gene per number of molecules of cyclophilin, as previously published(Mills et al., 2013; Somers et al., 2010). Primer sequences are: *CYCLOPHILIN*: F' GAA GAG TGC GAT CAA GAA CCC ATG AC, R' GTC TCT CCT CCT TCT CCT CCT ATC TTT ACT T; AAT(*SERPINA1*): F'AGG GCC TGA AGC TAG TGG ATA AGT, R' TCT GTT TCT TGG CCT CTT CGG TGT; *AFP*: F' CTA CCT GCC TTT CTG GAA GAA CTT TG, R'TCT GTT TCT TGG CCT CTT CGG TGT.

AAT pulse-chase radiolabeling. The kinetic of AAT post-translational intracellular processing and secretion was assayed via pulse-chase radiolabeling. Before radiolabelling, the patient-derived iPSC line B-16 and its isogenic, zinc finger nuclease-corrected daughter iPSC line B-16-C-2 were differentiated to hepatic stage and assayed via pulse-chase labelling using previously described methods(Ordóñez et al., 2013). Briefly, cells were starved in methionine (Met)- and cysteine (Cys)-free pulse medium for 90 min and then incubated with 1.3MBq of ^{35}S-Met/Cys for 30 min at 37°C to allow incorporation of radioactive amino acids. Cells were then washed and incubated in L-Met and L-Cys-supplemented chase medium for the time intervals indicated in the text before collection of cell supernatants and cell harvesting. Total protein was isolated from cell lysates using Nonidet lysis buffer (150mM NaCl, 50mM Tris-HCl pH 7.5, 1% Nonidet P-40) containing 25mM protease inhibitor mixture, followed by centrifugation. AAT was immunoprecipitated from lysates or from supernatants with a polyclonal anti-human α_1-antitrypsin antibody generated by the group of Prof. D.A. Lomas(Miranda et al., 2010) and resolved by 10% v/v SDS-PAGE. Radiolabelled AAT was visualized and quantified on a Cyclone Phosphor Imager (Packard Instrument Co.). Total densitometric value of the combined

lysate and supernatant at each time point were set at 100% and the value of each component displayed as a percent of the total.

Western Blot. PiZZ or WT iPSCs were differentiated to T16 or T18 before treatment with either CBZ, DMSO vehicle, or regular media as described for each experiment. Cell protein lysates were collected and separated in a 12% polyacrylamide gel before transfer onto a PVDF membrane. Membranes were probed with antibodies against LC3 (Sigma-Aldrich, # L7543), p62 (Abnova, #H00008878-M0), B-actin (Sigma-Aldrich, #A5316), KDEL (Grp78, Grp94; Enzo Life Sciences, # SPA-827-F), sXBP-1 (Biolegend, #619502), IκBα (Cell Signaling Technology, #9242), or GAPDH (Millipore, # MAB374). Signal was detected using goat anti-mouse or anti-rabbit HRP substrate (Biorad) on a LAS-4000 luminescent image analyzer (Fuji) and Image J software was utilized to measure densitometry.

Supplemental References:

Gouon-Evans, V., Boussemart, L., Gadue, P., Nierhoff, D., Koehler, C.I., Kubo, A., Shafritz, D.A., and Keller, G. (2006). BMP-4 is required for hepatic specification of mouse embryonic stem cell-derived definitive endoderm. Nat Biotechnol *24*, 1402–1411.

Miranda, E., Pérez, J., Ekeowa, U.I., Hadzic, N., Kalsheker, N., Gooptu, B., Portmann, B., Belorgey, D., Hill, M., Chambers, S., et al. (2010). A novel monoclonal antibody to characterize pathogenic polymers in liver disease associated with α 1-antitrypsin deficiency. Hepatology *52*, 1078–1088.
