# Supplementary material for: Emergence of a Stage-Dependent Human Liver Disease Signature with Directed Differentiation of Alpha-1 Antitrypsin-Deficient iPS Cells
Source: Stem Cell Reports. 2015 Apr 2;4(5):873–85. doi: 10.1016/j.stemcr.2015.02.021 (PMC4437473; doi:10.1016/j.stemcr.2015.02.021)
Supplement: Document S2. Article plus Supplemental Information [file mmc2.pdf]

Stem Cell Reports
Article
OPEN ACCESS

Emergence of a Stage-Dependent Human Liver Disease Signature with Directed Differentiation of Alpha-1 Antitrypsin-Deficient iPS Cells

Andrew A. Wilson,[1,10] Lei Ying,[2,10] Marc Liesa,[3] Charis-Patricia Segeritz,[4] Jason A. Mills,[2] Steven S. Shen,[5] Jyhchang Jean,[1] Geordie C. Lonza,[1] Derek C. Liberti,[1] Alex H. Lang,[6] Jean Nazaire,[7] Adam C. Gower,[5] Franz-Josef Müeller,[8] Pankaj Mehta,[6] Adriana Ordóñez,[9] David A. Lomas,[9] Ludovic Vallier,[4] George J. Murphy,[1] Gustavo Mostoslavsky,[1] Avrum Spira,[5] Orian S. Shirihai,[3] Maria I. Ramirez,[7] Paul Gadue,[2,11] and Darrell N. Kotton[1,11,*]

[1]Center for Regenerative Medicine (CReM) of Boston University and Boston Medical Center, Boston, MA 02118, USA
[2]Department of Pathology and Laboratory Medicine, The Children's Hospital of Philadelphia, Philadelphia, PA 19104, USA
[3]Evans Center for Interdisciplinary Research, Department of Medicine, Mitochondria ARC, Boston University School of Medicine, Boston, MA 02118, USA
[4]Wellcome Trust-Medical Research Council Cambridge Stem Cell Institute, Anne McLaren Laboratory for Regenerative Medicine and Department of Surgery, University of Cambridge, Cambridge CB2 0SZ, UK
[5]Division of Computational Biomedicine and Department of Pathology and Laboratory Medicine, Boston University School of Medicine, Boston, MA 02118, USA
[6]Physics Department, Boston University, Boston, MA 02215, USA
[7]The Pulmonary Center and Department of Medicine, Boston University School of Medicine, Boston, MA 02118, USA
[8]Zentrum für Integrative Psychiatrie, Universitätsklinikums Schleswig-Holstein, Kiel 24105, Germany
[9]Cambridge Institute for Medical Research, Cambridge CB0 2XY, UK
[10]Co-first author
[11]Co-senior author
*Correspondence: dkotton@bu.edu
http://dx.doi.org/10.1016/j.stemcr.2015.02.021
This is an open access article under the CC BY license (http://creativecommons.org/licenses/by/4.0/).

SUMMARY

Induced pluripotent stem cells (iPSCs) provide an inexhaustible source of cells for modeling disease and testing drugs. Here we develop a bioinformatic approach to detect differences between the genomic programs of iPSCs derived from diseased versus normal human cohorts as they emerge during in vitro directed differentiation. Using iPSCs generated from a cohort carrying mutations (PiZZ) in the gene responsible for alpha-1 antitrypsin (AAT) deficiency, we find that the global transcriptomes of PiZZ iPSCs diverge from normal controls upon differentiation to hepatic cells. Expression of 135 genes distinguishes PiZZ iPSC-hepatic cells, providing potential clues to liver disease pathogenesis. The disease-specific cells display intracellular accumulation of mutant AAT protein, resulting in increased autophagic flux. Furthermore, we detect beneficial responses to the drug carbamazepine, which further augments autophagic flux, but adverse responses to known hepatotoxic drugs. Our findings support the utility of iPSCs as tools for drug development or prediction of toxicity.

INTRODUCTION

Alpha-1 antitrypsin deficiency (AATD) is a common genetic cause of both liver and lung disease affecting an estimated 3.4 million patients worldwide (de Serres, 2002). The most common disease variant is caused by an inherited single base pair mutation of the *SERPINA1* gene that results in a glutamate to lysine substitution (Glu342Lys) and production of a mutant version of the protease inhibitor AAT, known as Z AAT (Brantly et al., 1988). Z AAT protein is prone to misfolding and polymerization and has reduced capacity to inactivate neutrophil elastase, its primary substrate, resulting in both toxic gain-of-function and loss-of-function phenotypes (Brantly et al., 1988; Crystal, 1990; Lomas et al., 1992; Perlmutter and Pierce, 1989). AATD has proven difficult to model experimentally in mice and in human primary or immortalized cells, a factor that has limited the progress of research aimed either at elucidating mechanisms of disease or developing new treatment approaches. Studies based on transgenic PiZ mice or immortalized cell lines engineered to express the human mutant Z AAT allele or on primary human hepatocytes have provided significant insights into the pathogenesis of AATD-associated liver disease. These studies have demonstrated that polymerization of Z AAT protein in the ER results in activation of an ER overload response (Hidvegi et al., 2005; Lawless et al., 2004), characterized by chronic activation of the proinflammatory transcription factor NF-κB (Pahl and Baeuerle, 1995), together with activation of ER stress-specific caspases (Hidvegi et al., 2005). Each of these models, however, has shortcomings that potentially limit its ability to delineate the mechanisms of a disease that develops over time in human liver tissue.

Recently, the discovery of induced pluripotent stem cells (iPSCs) (Takahashi and Yamanaka, 2006) has made it possible to model a variety of genetic diseases in vitro using patient-derived stem cells (Ebert et al., 2009; Park et al., 2008; Rashid et al., 2010). The differentiated progeny of patient-derived iPSCs provide disease-relevant cells in an individual patient's genetic background, potentially allowing

personalized, in vitro assessments of disease pathogenesis and treatment responsiveness. As with human clinical trials, however, studies utilizing multiple patient-derived iPSC lines introduce the complexity of genetic variability. This experimental approach increases the likelihood that findings will be generalizable to a population rather than specific to an individual, but also potentially decreases the signal-to-noise ratio.

Here we sought to apply an iPSC-based approach to study generalizable effects of the Z mutation, rather than the effects of any single individual's genetic background. To do so, we incorporated iPSC lines derived from multiple individuals homozygous for the Z allele (termed PiZZ), ensuring the inclusion of genetic heterogeneity. We found that the transcriptional profile of iPSCs derived from individuals homozygous for the Z allele diverges from normal controls only upon differentiation to the hepatic stage, when the AAT gene is expressed. Expression of 135 genes distinguishes PiZZ iPSC-hepatic cells from controls at this stage, providing potential clues to liver disease pathogenesis. PiZZ iPSC-hepatic cells model key features of AATD-associated liver disease, including intracellular accumulation and reduced secretion of AAT protein as well as increased autophagic flux. Augmented autophagic flux can be further enhanced in iPSC-hepatic cells upon treatment with the drug carbamazepine (CBZ), an observation first made in transgenic PiZ mice (Hidvegi et al., 2010) that has important implications for treating patients with AATD-related liver disease. Finally PiZZ iPSC-hepatic cells exhibit increased sensitivity to a panel of hepatotoxic drugs, including the common analgesic acetaminophen, confirming their potential application as tools for drug discovery or prediction of toxicity.

RESULTS

To develop iPSC-based model systems of disease, we first prepared a bank of >60 iPSC clones (ten clones per donor; partial set and reprogramming methodology described previously [Mills et al., 2013; Somers et al., 2010]) derived from the dermal fibroblasts of three control individuals without any known disease and three recruited volunteers previously diagnosed with AATD due to homozygous inheritance of mutant Z alleles encoding the AAT protease inhibitor (PiZZ genotype, a common monogenic cause of hepatic cirrhosis [Eriksson et al., 1986]). Pluripotency of the resulting cells was confirmed in teratoma assays (Somers et al., 2010), as well as by Pluritest global transcriptomic analysis (Müller et al., 2011; Figure S1).

We previously published microarray profiling of the global transcriptomes of nine iPSC lines made from three normal donors (three iPSC lines/donor) and three embryonic stem cell (ESC) lines. We determined that donor-to-donor genetic variability in stem cell phenotype (defined as the global transcriptome expressed in the pluripotent/undifferentiated state) rather than clone-to-clone variability per donor was most responsible for variance in gene expression among cell lines (Mills et al., 2013). In addition to gene expression, we found the propensity to differentiate into a particular fate was also due mainly to genetic background as opposed to clonal variability, consistent with others' work focused on the relative contribution of donor, cell type of origin, and reprogramming approach to differentiation efficiency (Kajiwara et al., 2012). These data prompted us to select only a single clone per donor for all disease-modeling experiments, as individual clones of a given genetic background tend to behave similarly.

PiZZ iPSC-Hepatic Cells Demonstrate Accumulation of Intracellular AAT Protein and Expression of a Transcriptomic Disease-Specific Signature

We sought to test the hypothesis that a single transgene-free iPSC clone from each donor could be used to detect disease-specific differences between the normal cohort and the PiZZ cohort, anticipating that differences would emerge only at a developmental stage in which the mutant AAT gene is expressed. Employing single iPSC clones from each normal or PiZZ individual as well as normal ESC control clones (n = 3 per group), we utilized sequential growth factor stimulation with serum-free conditions that we established previously (Cheng et al., 2012; Somers et al., 2010) for the directed differentiation of pluripotent cells into definitive endoderm followed by early hepatocyte-like lineages (Figure 1A; Figure S2).

We monitored all nine cell lines across three time points of hepatic directed differentiation, representing three developmental stages: (1) undifferentiated (T0), (2) definitive endoderm (T5), and (3) early hepatocyte (T24). No significant differences in differentiation efficiency were observed between PiZZ and normal lines as measured by flow cytometric quantitation of markers of pluripotency (SSEA3, TRA-1-81), definitive endoderm (CKIT/CXCR4), and hepatic (AAT/FOXA1) lineages at each developmental stage (Figure 1B; Figure S3). As previously reported by others (Choi et al., 2013; Rashid et al., 2010), we found intracellular accumulation of AAT protein in all three of our PiZZ iPSC lines at the hepatic stage (T24). The mean fluorescence intensity (MFI) of immunostained intracellular AAT protein measured by flow cytometry was logarithmically elevated in iPSC-hepatic cells from three of three PiZZ individuals compared to the six other lines (Figures 1B and 1C). This accumulation was neither due to differences in AAT (*SERPINA1*) expression at the RNA level nor to generalized accumulation of liver-related proteins, such

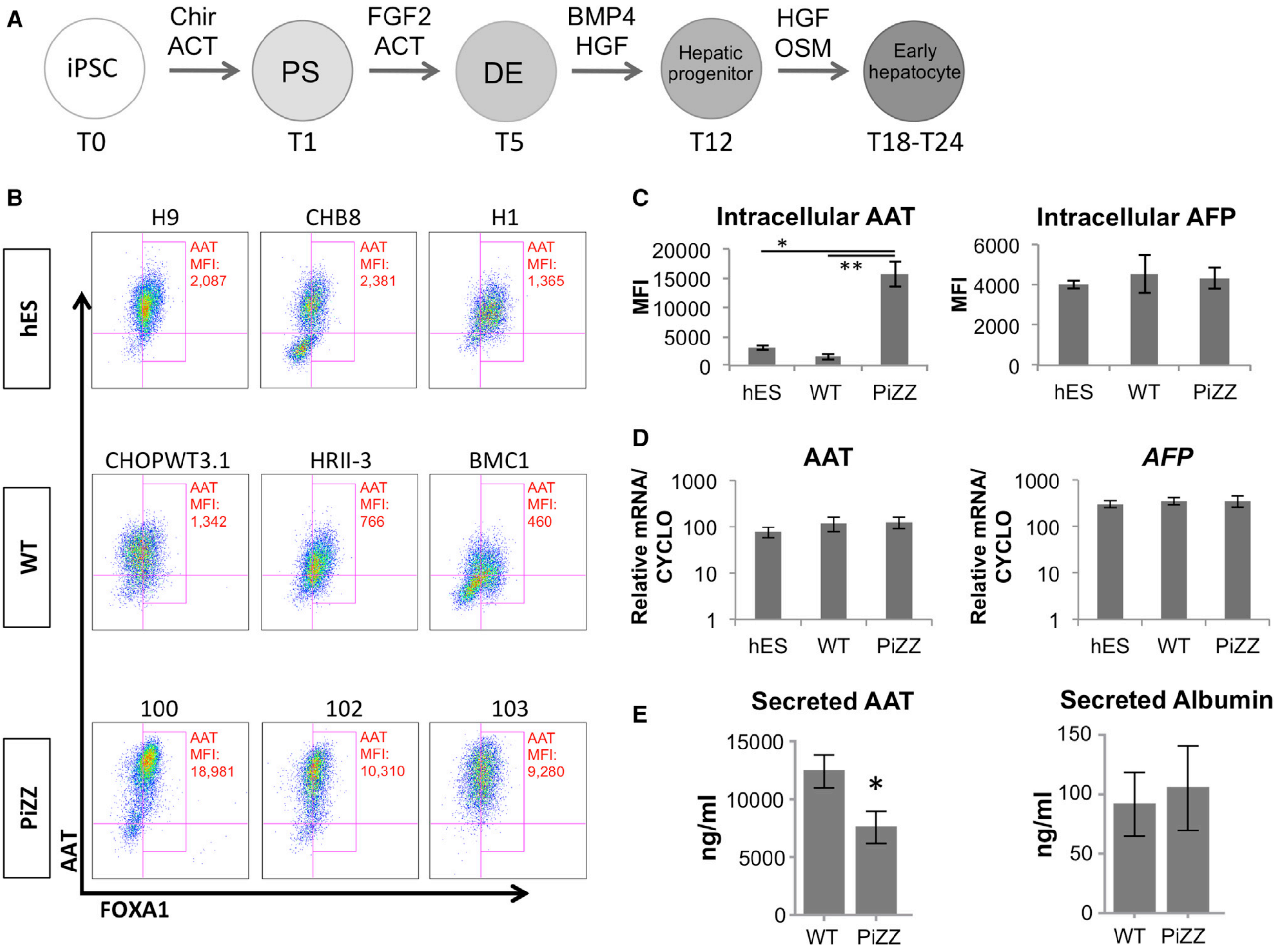

Figure 1. Patient-Derived PiZZ iPSC-Hepatic Cells Exhibit Intracellular AAT Retention

(A) Schematic overview of iPSC-hepatic directed differentiation protocol. $^*p < 0.05$, $^{**}p < 0.01$ by one-way ANOVA.

(B) Flow cytometry of fixed, permeabilized iPSC-hepatic cells using anti-AAT and anti-FOXA1 antibodies in nine genetically distinct ESC or iPSC lines at T24.

(C) MFI of AAT and AFP in PiZZ iPSC-hepatic cells compared to WT iPSC- or ESC-hepatic cells.

(D) Quantification of AAT (*SERPINA1*) and *AFP* mRNA levels in iPSC-hepatic cells.

(E) ELISA of secreted AAT and albumin in cell supernatants at T24. $^*p < 0.05$ by two-tailed t test. PS, primitive streak; DE, definitive endoderm; ACT, Activin A; Chir, Chir99021; OSM, oncostatin M. n = 3 independent experiments. Data are represented as mean ± SEM.

as AFP (Figures 1C and 1D). AAT secretion was significantly decreased in the PiZZ iPSC-hepatic cells while albumin secretion was similar to controls (Figure 1E), suggesting that the increase in intracellular AAT was due to a failure to properly process and secrete this protein.

To address definitively whether accumulation of intracellular protein observed in PiZZ iPSC-hepatic cells was due to reduced AAT flux and specifically resulted from the Z mutation, we performed pulse-chase labeling experiments. Employing this classical assay for defining the kinetics of AAT protein processing and secretion, we utilized an AATD iPSC line in which both mutant Z alleles had undergone zinc-finger-mediated gene correction, comparing the corrected clone to its parental, syngeneic PiZZ line (Yusa et al., 2011). Published studies in PiZ mice (Graham et al., 1990), HeLa cells (Hidvegi et al., 2005), and primary human fibroblasts (Wu et al., 1994) engineered to express the mutant Z protein previously have demonstrated altered posttranslational modification of AAT protein and delayed secretion in Z AAT-expressing cells. In keeping with these heterologous models, we found delayed processing of the native 52-kDa AAT protein to its mature 55-kDa form in PiZZ iPSC-hepatic cells together with delayed AAT secretion compared to its gene-corrected parental line and a wild-type (WT) control line (Figures 2A–2C).

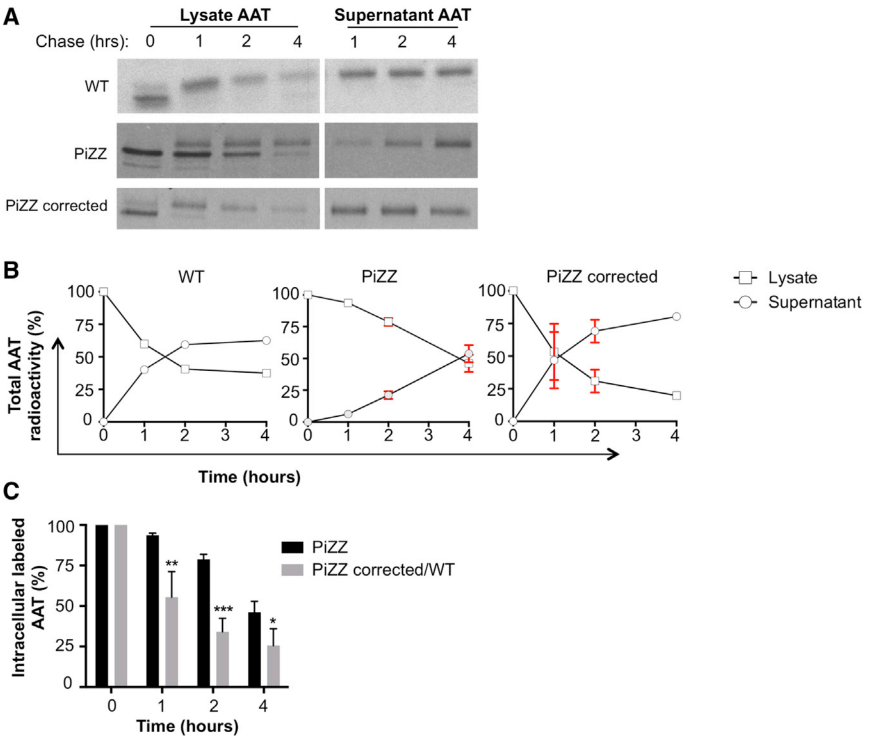

Figure 2. Pulse-Chase Radiolabeling Demonstrates Altered Post-translational AAT Processing and Secretion Kinetic in PiZZ iPSC-Hepatic Cells

(A) Quantification of radiolabeled AAT protein from cell lysates or supernatants at indicated time points after ^{35}S-Met/Cys radiolabeling. Both the 52-kDa native AAT protein and its 55-kDa mature are detected in WT, PiZZ, and gene-corrected PiZZ (to PiMM, following ZFN-mediated correction of the Z mutation) iPSC-hepatic cells.

(B) Kinetic of aggregate cell lysate and supernatant radiolabeled AAT secretion data represented in (A).

(C) Quantification of aggregate intracellular radiolabeled AAT protein during the chase period. n = 4 (PiZZ) or n = 3 (PiZZ corrected/WT) independent experiments. Data are represented as mean ± SD. *p < 0.05, **p < 0.01, ***p < 0.001 by two-tailed t test.

Having demonstrated decreased AAT protein flux and intracellular AAT accumulation in disease-specific iPSC-hepatic cells, we next performed microarray analyses of the global transcriptomes (mRNA and microRNA [miRNA]) and DNA methylomes (methylated CpG dinucleotides) of these nine cell lines at each differentiation stage (Figure 3). Principal component analysis (PCA) of mRNA, miRNA, and DNA microarrays from these 27 samples revealed tight clustering of samples by developmental stage rather than by donor (Figure 3A). To statistically interrogate the kinetics of global gene expression of each cell line during directed differentiation to liver, we employed two-way ANOVA of all 27 samples to identify: (1) genes that are differentially expressed among days 0, 5, and 24 of differentiation (time effect); (2) genes that are differentially expressed among ESC, normal iPSC, and PiZZ iPSC lines (cell type effect); and (3) gene expression differences among cell types that are modulated by differentiation (interaction effect of cell type and time). We have described previously this approach of comparing the entire gene expression kinetic with differentiation as a robust method for detecting subtle differences between iPSCs and ESCs when using small sample sizes (Christodoulou et al., 2011). Directed differentiation was associated with a large number of gene expression changes (11,232 of ~30,000 probe sets were significantly associated with the time effect at false discovery rate (FDR) < 0.01; Figure 3B). Importantly, all master endodermal transcriptional regulators assessed (*FOXA2*, *GATA4* and *6*, *SOX17*, *HEX*, and *HNF4A*) were upregulated as expected at T5, and known markers of hepatocytes (AAT, *TTR*, *FBG*, *TF*, *ALB*, CYP450 enzymes, and hepatitis virus receptors) were upregulated at T24 (Figure 3B; Figure S2). As expected, AAT was not detectably expressed at T0 or T5; however, AAT was the third most significantly upregulated gene of 30,000 tested transcriptomic probe sets in the genome at the hepatic stage (ranking by FDR-adjusted p value; Figure 3B).

To develop a putative PiZZ disease-specific transcriptomic signature based on this iPSC model of differentiation, we next analyzed the interaction effect. We identified differentially expressed genes based on the interaction of time and cell type (419 genes at FDR < 0.25; Figure 3C; 85 genes at FDR < 0.1). To determine which cell type and differentiation stage were responsible for differences in gene expression during directed differentiation, we first employed unsupervised clustering of the top 1,000 genes differentially expressed with time, and we found the 27 samples clustered predominantly by developmental stage. However, clustering by cell type emerged at the hepatic stage of differentiation based on the height of the clustered dendogram at T24 (Figure 3B). Second, we performed a post hoc analysis of the cell type effect using moderated t tests to identify which cell type was responsible for the cell type effect. These tests revealed that the cell type effect was due entirely to the PiZZ iPSC clones, with no cell type differences between ESCs versus normal iPSCs. Third, we

performed post hoc moderated t tests of the interaction effect to identify 135 differentially expressed transcripts at T24 that distinguished normal versus PiZZ iPSC-hepatic cells, hereafter referred to as a disease-specific transcriptomic signature (Figure 3D; Table S1). The five genes most differentially expressed in this list were then validated by qPCR (*DNAH5*, *CASP4*, *CFH*, *HAVCR2*, and *ERAP2*; Figures 3D and 3E). Together these statistical analyses indicated that the subtle divergence in gene expression kinetics among cell types undergoing hepatic directed differentiation is due mainly to the divergence of PiZZ disease-specific clones upon reaching the hepatic stage (Figures 3C and 3D).

We next looked at differences in miRNA (by miRNA arrays) and methylation status (by genome-wide Illumina 450K methylation array) of ESCs and iPSCs during directed differentiation. The large variance in miRNA expression profiles precluded finding any statistically significant miRNA expression differences between diseased and normal cells at any stage; however, clear differences were evident in all cell types based on developmental stage (Table S2; Figure 3A). In keeping with the mRNA expression differences that emerged between PiZZ iPSCs versus normal iPSCs and ESCs during directed differentiation, the number of DNA methylation differences between diseased and normal pluripotent stem cells also increased from 23 at T0 to 195 at T24 (Table S2). To determine whether differences in DNA methylation could potentially account for altered gene expression in PiZZ iPSC-hepatic cells, we next analyzed CpG sites in the promoter regions of differentially expressed genes (Figure 3A). At the hepatic stage (T24), one or more CpG sites were differentially methylated in 13 of the 135 interaction effect genes (Table S3) comprising the PiZZ disease-specific transcriptomic signature. CpG methylation and expression levels of these genes were significantly anti-correlated. While not in themselves proof of causality, together these findings suggest an association between differences in gene expression in hepatic-stage PiZZ and normal iPSCs and alterations in the methylation state of the promoter regions of a subset of these genes.

Hepatic Pathways Responding to Intracellular Accumulation of Mutant Z AAT

Next we sought to employ our disease-specific transcriptomic signature and the iPSC model system to better understand the pathogenesis of AATD-related liver disease. Based on established mouse models, human liver biopsy specimens, and human heterologous cell line studies, mutated Z AAT protein has been demonstrated to induce liver disease through a toxic gain-of-function resulting predominantly from the accumulation of misfolded, insoluble aggregates of mutant protein in the rough ER (Lomas et al., 1992). Indeed, our interaction effect gene signature revealed differential expression of several individual gene markers associated with oxidant stress or known to localize to the ER. For example, 27 genes on the interaction list, including *ERAP2*, were grouped under the gene ontology heading of ER (Figure 3; Table S1). Notably, the second most significantly differentially expressed transcript within the interaction effect disease-specific signature was the ER stress-specific caspase family member, caspase-4, validated by qPCR to be upregulated in PiZZ iPSC-hepatic cells compared to controls (Figure 3E). Activation of mouse caspase-12 and human caspase-4, its putative homolog (Hitomi et al., 2004), have been demonstrated in Z AAT-expressing mouse and human cell lines and in PiZ mouse livers (Hidvegi et al., 2005).

Markers of the unfolded protein response (UPR) were not present in the PiZZ disease-specific signature in concordance with previous studies that have detected no evidence of hepatic UPR resulting from misfolded Z AAT in mouse models or cell lines or human liver biopsy specimens (Hidvegi et al., 2005). Since T24 represents a time in culture >1 week after the onset of initial AAT protein expression (Figure S3), we sought to determine whether a UPR was detectable in the disease-specific cells closer to the time of initial expression of the mutant Z AAT protein. Indeed, by qPCR at T18 of differentiation, we found significant upregulation of *ATF4* and spliced *XBP-1* mRNA only in the PiZZ cells (Figure 3F). To evaluate whether these findings extended to differences in protein expression, we next performed western blots on whole-cell lysates collected from T16–T18 cells, and we found increased levels of spliced XBP-1 and the ER resident chaperone molecule Grp-78 in PiZZ cells compared to controls. Because the UPR is likewise known to activate NF-κB via degradation of its inhibitor, IκBα, mediated by IRE-1 oligomerization and binding to TRAF2 (Kaneko et al., 2003), we also assayed IκBα and found it to be decreased in PiZZ cells at this stage of differentiation (Figure 3G; Figure S4). Together, these findings are consistent with activation of the UPR in hepatocytes by misfolded Z AAT protein, potentially in a manner that is context specific.

The autophagy pathway is a key cellular mechanism thought to be responsible for processing intracellular Z AAT protein aggregates, based on documented increases in autophagic flux in mammalian cell lines and mouse models featuring overexpression of human Z AAT (Teckman and Perlmutter, 2000; Teckman et al., 2002). Indirect evidence of altered autophagy in humans derives from observation of increased numbers of autophagic vesicles in PiZZ liver biopsy specimens (Teckman and Perlmutter, 2000). We therefore sought to monitor and manipulate autophagic flux in iPSC-derived hepatic lineages. We first transduced PiZZ versus normal iPSC-hepatic cells with a

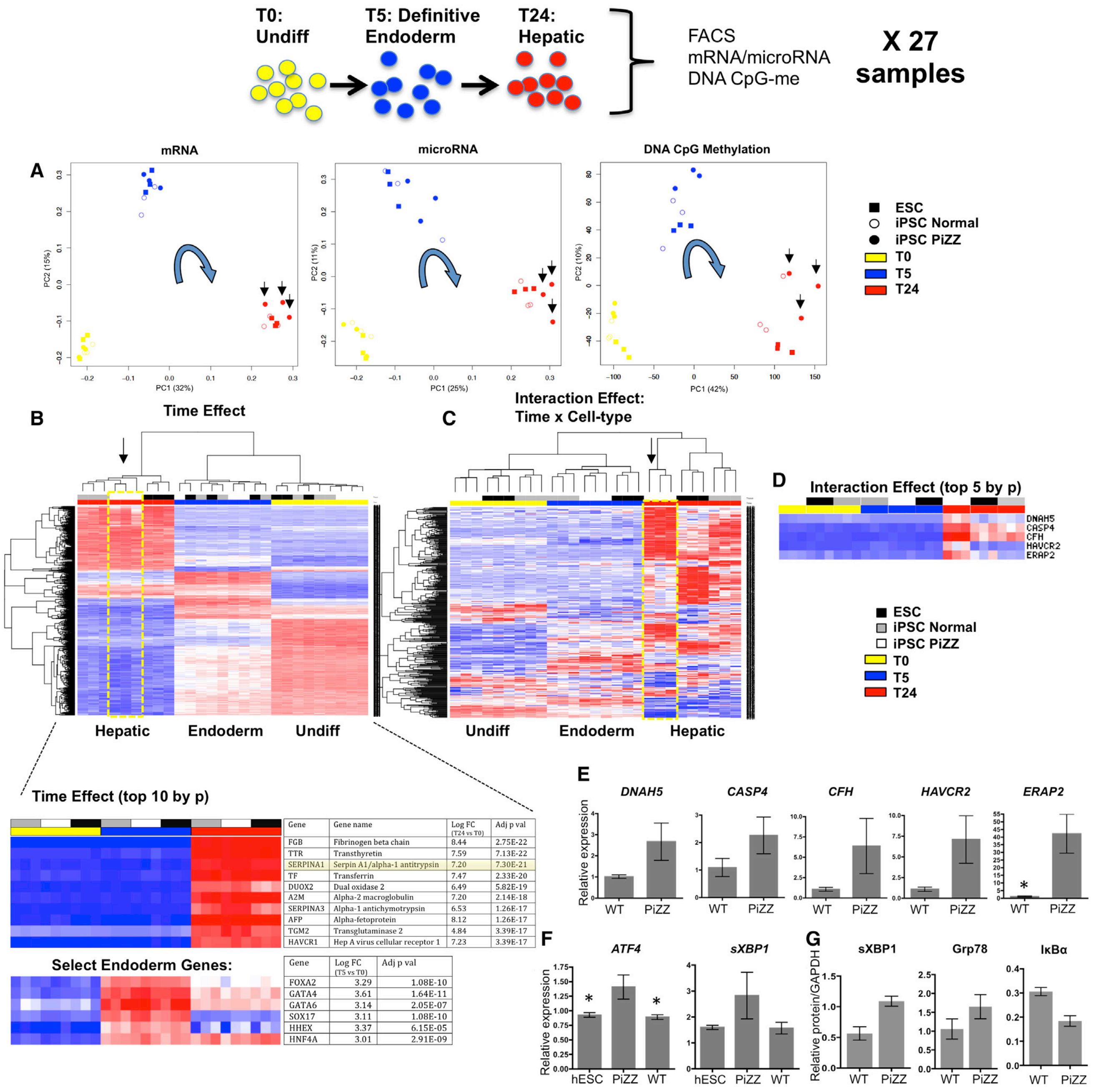

Figure 3. Genome-wide Transcriptomic and Epigenomic Analysis of PSCs at Key Developmental Stages

ESCs (n = 3) and patient-derived normal/WT (n = 3) or PiZZ (n = 3) iPSCs were analyzed at T0, T5, and T24 for a total of 27 samples.

(A) PCA demonstrates clustering predominantly by developmental stage (arrows indicate hepatic-stage PiZZ cells).

(B) Unsupervised hierarchical clustering of 27 samples analyzed by two-way ANOVA for time and cell type effects demonstrates differential gene expression with directed differentiation. The top ten genes upregulated with differentiation (middle) as well as expression levels of select endoderm genes at T5 (bottom) are listed.

(C) Analysis of gene expression differences among cell types that are modulated by differentiation (interaction effect of time and cell type) reveals 419 differentially expressed genes (FDR < 0.25; 85 genes at FDR < 0.1).

(D) Expression levels of the top five genes differentially expressed between PiZZ and normal iPSC-hepatic cells as identified by a post hoc moderated t test of the interaction effect gene set.

(E) qPCR validation of relative mRNA expression levels of genes from (D). n = 3 independent experiments. *p < 0.05 by two-tailed t test.

(legend continued on next page)

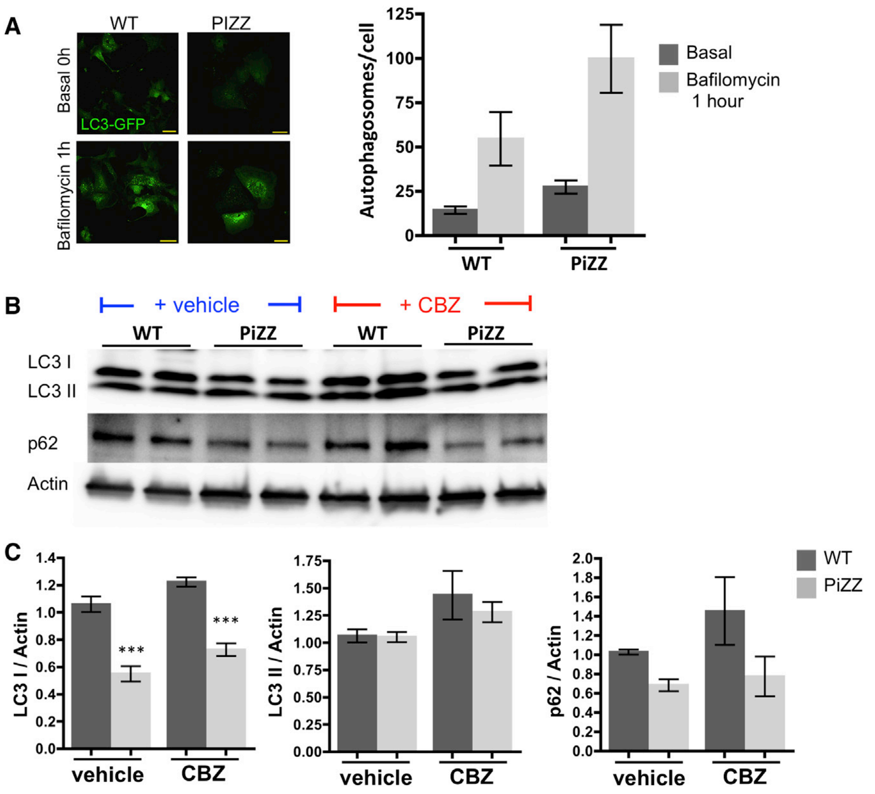

Figure 4. Enhanced Autophagic Flux in PiZZ iPSC-Hepatic Cells

(A) Differentiating iPSCs were transduced with a lentivirus encoding an LC3-GFP fusion protein. Following differentiation to the hepatic stage, LC3-GFP+ autophagosomes are quantified in the presence or absence of bafilomycin, an inhibitor of lysosomal fusion. Data are representative of one to three biological replicates per group per condition. Scale bars, 50 μM.

(B) Protein levels of the autophagosome components LC3-I and -II, together with p62 are quantified by western blot in WT and PiZZ iPSC-hepatic cells in the presence and absence of CBZ treatment.

(C) Protein levels from (B) are displayed graphically and normalized to actin (n = 3 biological replicates per group). Data are represented as mean ± SEM. ***$p < 0.001$ by two-tailed t test.

lentiviral LC3-GFP reporter (Twig et al., 2008) to count autophagosomes per cell. After treating T18 cells for 1 hr with the autophagosome clearance blocker bafilomycin, we observed more autophagosomes per cell in the PiZZ group (n = 50–60 cells per group; Figure 4A). Because these findings are consistent both with increased autophagosome formation and clearance in PiZZ iPSC-hepatic cells, we next determined LC3-I, LC3-II, and p62 protein levels in iPSC-hepatic cells by western blot analysis (Figures 4B and 4C). We found decreased p62 and decreased LC3-I with no significant changes in LC3-II in PiZZ iPSC-hepatic cells compared to controls, indicating increased clearance of autophagosomes in PiZZ cells. Together, these data indicate that increased autophagic flux is present in the PiZZ iPSC-hepatic cells, consistent with prior studies in other models (Hidvegi et al., 2010; Teckman and Perlmutter, 2000).

To determine whether drug treatment could further augment autophagic flux in iPSC-hepatic cells, we employed CBZ treatment based on publications by Perlmutter et al. who found this drug augments flux and reduces the burden of accumulated intracellular Z AAT protein in transgenic mice and heterologous cell lines. We observed increased LC3-II and LC3-I in both normal and PiZZ iPSC-hepatic cells, indicating increased autophagosome formation with CBZ treatment. The differences between normal and PiZZ cells were maintained after treatment, indicating increased clearance and increased formation of autophagosomes in PiZZ cells. However, the increase in p62 after CBZ treatment, likely resulting from increased autophagosome formation and accumulation, was greater in normal cells (~40%) than in PiZZ cells (~10%) (Figures 4B and 4C). Together, these findings provide evidence in human patient samples that supports and extends findings from prior non-human model systems demonstrating increased baseline autophagic flux in PiZZ-producing cells that is further augmented by CBZ.

iPSC-Hepatic Cells Serve as a Model of Disease-Specific Drug Therapy

Having demonstrated that CBZ is able to augment autophagic flux in human iPSC-hepatic cells, we next sought

(F) qPCR quantification of *ATF4* and spliced *XBP-1* mRNA expression at early hepatic stage (T18) in ESCs and PiZZ or normal iPSCs. n = 3 independent experiments. *$p < 0.05$ by one-way ANOVA.

(G) Densitometric quantification of sXBP-1, Grp78, and IκBα protein expression levels by western blot of whole-cell lysates prepared from PiZZ and normal iPSCs (n = 3 biological replicates per group) at T16–T18 and normalized to GAPDH. Data are represented as mean ± SEM.

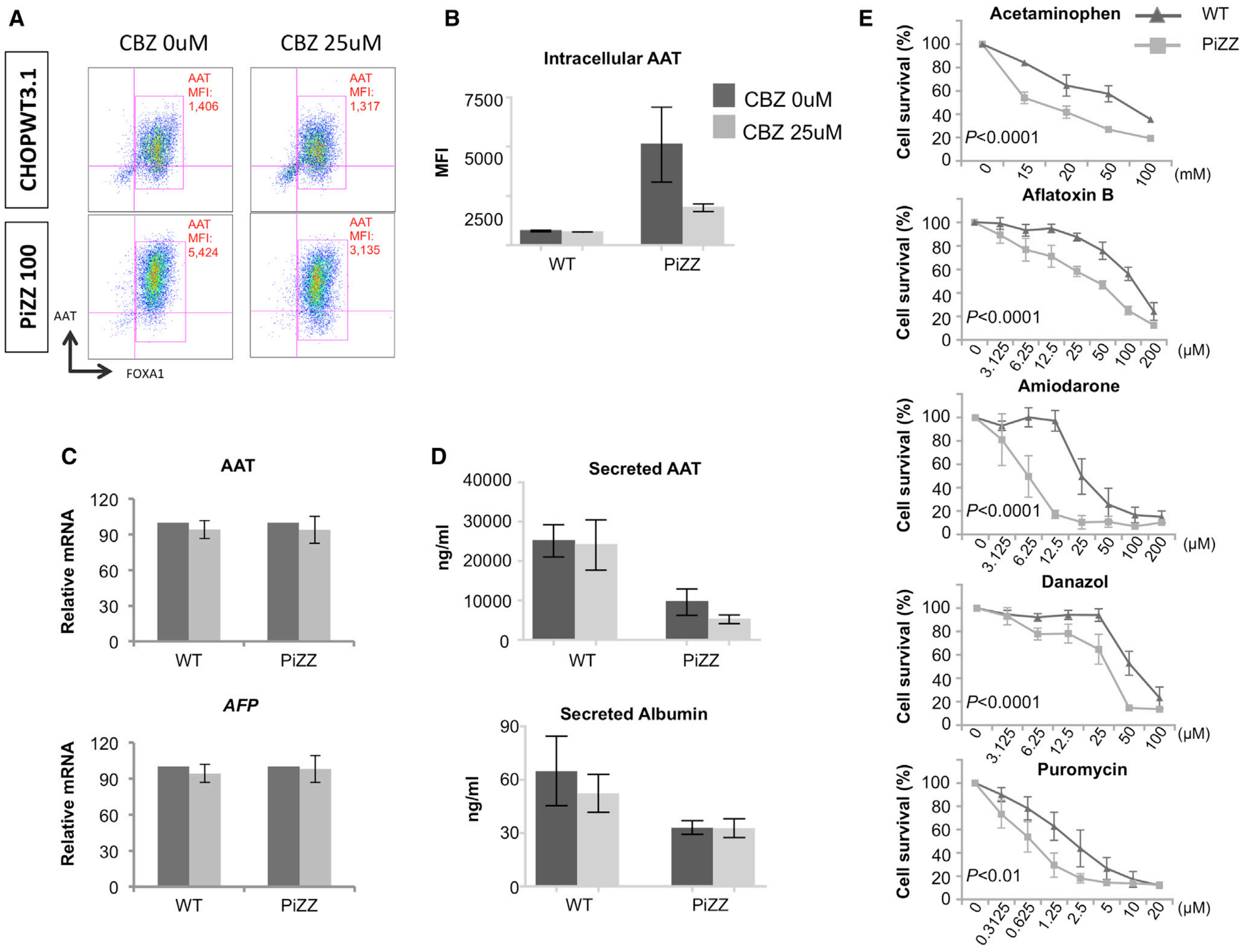

Figure 5. PiZZ and WT iPSC-Hepatic Cells Diverge in Their Response to Drug Treatment

(A) Representative flow cytometric analysis of AAT MFI in WT and PiZZ iPSC-hepatic cells (T24) following a 48-hr treatment with control vehicle or CBZ.

(B) Aggregate data from three WT and three PiZZ lines, including data represented in (A), are quantified graphically. n = 3 independent experiments.

(C) AAT (*SERPINA1*) (top) and *AFP* (bottom) mRNA expression levels with and without CBZ treatment, expressed as a percentage of untreated levels.

(D) Concentration of AAT (top) and albumin (bottom) in iPSC-hepatic cell supernatants measured by ELISA. n = 3 independent experiments.

(E) Viability of PiZZ and WT iPSC-hepatic cells is quantified by MTT assay after incubation with acetaminophen (4 hr) or aflatoxin B, amiodarone, danazol, or puromycin (4 days) at the indicated concentrations. Survival percentage at each concentration is determined as a percentage of vehicle control treatment. n = 3 independent experiments. Data are represented as mean ± SEM. P values represent contribution of cell type to variance as determined by two-way ANOVA.

to determine whether augmented flux would decrease intracellular AAT accumulation in PiZZ cells. We found all three PiZZ donor lines demonstrated CBZ dose-dependent amelioration in accumulation of intracellular AAT protein, whereas no significant difference was observed in the low levels of intracellular AAT in normal cells before versus after treatment (Figures 5A and 5B; Figure S5). CBZ treatment did not result in changes in expression of AAT or differentiation marker genes, indicating that the observed reduction in intracellular AAT was not due to the inhibition of AAT transcription or to suppressed hepatic differentiation of iPSCs (Figure 5C). Finally, CBZ did not affect levels of secreted AAT (or an alternative hepatic secreted protein, albumin) in iPSC-hepatic cell supernatants (Figure 5D).

PiZZ iPSC-Hepatic Cells Display Increased Susceptibility to Drug-Induced Toxicity

A hallmark of diseased hepatocytes in humans is increased susceptibility to environmental insults or drug toxicity compared to normal hepatocytes. While iPSC-hepatic cells recently have been evaluated as a potential platform for detecting drug toxicity (Sjogren et al., 2014; Szkolnicka et al., 2014), the relative sensitivity of diseased and normal differentiated iPSCs to hepatotoxic agents has not to our knowledge been examined. Hence, we sought to test whether PiZZ iPSC-hepatic cells are more susceptible to the toxic effects of a common pharmaceutical analgesic, acetaminophen. Indeed, we found PiZZ iPSC-hepatic cells were more susceptible to acetaminophen-induced toxicity than normal controls, as evidenced by lower viability at each dose of acetaminophen tested (Figure 5E). To evaluate the specificity of this finding, we extended our evaluation to include a panel of four additional drugs (amiodarone, danazol, puromycin, and aflatoxin-B) known to cause hepatotoxicity through several mechanisms. In each case, PiZZ cells were more sensitive than normal control iPSC-hepatic cells to drug-induced toxicity, consistent with a broad susceptibility to hepatotoxic agents (Figure 5E).

DISCUSSION

In summary, our results demonstrate the application of iPSCs from multiple donors to model disease, predict drug efficacy or toxicity, and unveil mechanisms not easily studied in vivo in humans or mouse models. We present a bioinformatic approach to defining disease-specific signatures for monogenic diseases based on the delineation of a global transcriptomic signature that emerges in diseased cells only upon reaching the developmental stage at which the mutant protein is expressed. Using this approach, we found PiZZ iPSC lines did not differ from control iPSCs or ESCs when in the undifferentiated or endodermal states but diverged at the hepatic stage, exhibiting evidence of intracellular accumulation of mutant AAT protein, a transcriptomic disease-specific signature that likely represents the downstream effect of accumulated protein aggregates, and activation of pathways known to respond to the burden of misfolded intracellular protein, such as augmented autophagic flux.

In these studies, we included multiple donors in each cohort, rather than multiple isogenic clones, in an approach intended to mimic the design of human clinical trials. This allowed us to determine whether, within a genetically diverse cohort, we could detect disease-specific differences emerging above the known phenotypic variation among normal individuals. Indeed, we were able to detect disease-state-specific differences in gene expression among cohorts, in addition to some differences in levels of intracellular protein accumulation among clones, that could represent genetic differences in protein processing or the cellular response to misfolded proteins that has been postulated to exist among individuals (Pan et al., 2009; Wu et al., 1994).

Human iPSC-hepatic cells derived using our differentiation protocol were similar to primary human fetal hepatocytes in terms of expression levels of a subset of hepatic genes. These results are consistent with other published protocols (Rashid et al., 2010; Si-Tayeb et al., 2010) demonstrating differentiation of hepatic cells that were incomplete in their maturity, as evidenced by persistent, high levels of *AFP* expression that were similar to fetal levels in our experiments. This hurdle in directing differentiation of pluripotent stem cells to fully mature differentiated cells has been seen across germ layers and cell types (Baxter et al., 2015; Smith et al., 2013), reflects the general state of the field, and is the focus of a growing number of investigators (Ogawa et al., 2013; Shan et al., 2013). As our data and the published literature suggest (Leung et al., 2013; Rashid et al., 2010; Suzuki et al., 2014), however, the ability to fully mature a cell in vitro might not be necessary to model and study key disease features when disease-causative genes are expressed at high levels.

We used our iPSC human disease model to assess both well-accepted and controversial pathways for handling protein misfolding that have been interrogated using other approaches. Our report utilizes the classical pulse-chase labeling technique to quantify the kinetics of AAT processing and secretion using human patient-derived hepatocyte-like cells, demonstrating the ability of iPSC-hepatic cells to model a key feature of Z AAT protein-driven cellular dysfunction. In cells accumulating misfolded, insoluble Z AAT protein polymers, the autophagy pathway is activated in an attempt to deal with this toxic protein accumulation. Our studies document increased formation as well as increased clearance of autophagosomes in PiZZ iPSC-hepatic cells, consistent with augmented autophagic flux. These findings are in accord with those previously observed in mouse embryonic fibroblasts (MEFs), cell lines, and transgenic mice overexpressing human Z AAT. Increased autophagosome numbers have been observed in liver biopsy specimens from PiZZ individuals (Teckman and Perlmutter, 2000), but it has not been possible previously to measure flux in their tissues. Our report extends to human hepatic cells the observation made in PiZ transgenic mice (Hidvegi et al., 2010) that further CBZ-induced augmentation of this flux ameliorates intracellular accumulation of mutant protein.

A second cellular stress pathway implicated in the setting of accumulated intracellular Z AAT protein is the UPR, postulated to link Z AAT polymer-induced cellular injury

and downstream development of liver disease, a poorly understood progression (Lawless et al., 2004; Perlmutter et al., 2007). Previous studies of PiZZ liver disease in other model systems have not detected a UPR, despite its known role in the cellular response to high volumes of protein misfolding (Hidvegi et al., 2005). Our detection of a UPR, early during the hepatic differentiation of PiZZ iPSCs, thus contrasts with these studies, though it is in keeping with recent patient-based findings in circulating monocytes from PiZZ individuals (Carroll et al., 2010).

The potential of iPSC-based prediction of drug toxicity is a topic that has generated wide interest as protocols for directed differentiation have progressed. Our finding that PiZZ iPSC-hepatic cells exhibit increased sensitivity to an assortment of hepatotoxic drugs, including the analgesic acetaminophen, are in accord with the common clinical recommendation that patients with liver disease limit acetaminophen intake and suggest the possibility that they may be at risk from other medications as well. While our findings likewise reinforce the potential application of iPSC-derived cell types for drug toxicity testing, further studies to evaluate mechanisms by which specific agents induce toxicity in iPSC-hepatic cells versus primary human hepatocytes are likely to be important in understanding their predictive capacity (Sjogren et al., 2014).

In summary, our findings provide both global, genome-wide and focused, pathway-based views of disease-specific iPSCs as they differentiate in vitro into a target lineage responsible for AATD pathogenesis in patients. Our results provide comprehensive genetic and epigenetic databases for those interested in modeling human endoderm and liver development or liver disease onset. Future work can now focus on testing whether iPSC lines derived from each individual patient also have the capacity to model distinct patient-to-patient disease severity and clinical phenotypes known to occur among individuals with the same monogenic AATD mutation. Our results demonstrating drug responses in AATD iPSC-hepatic cells also raise the important question of whether individual patient iPSC lines also might predict in vivo individual therapeutic responses or toxicities to drugs, a hypothesis that will likely be tested in partnership with clinical drug trials in the years ahead.

EXPERIMENTAL PROCEDURES

Production, Characterization, and Maintenance of PSCs

The recruitment of human subjects and all iPSC studies were approved by the Boston University Institutional Review Board (BUMC IRB H-27636). Human dermal fibroblast-derived iPSCs were generated and characterized to confirm pluripotency as previously described (Mills et al., 2013; Somers et al., 2010; Terrenoire et al., 2013). Where indicated, clones underwent additional evaluation by pluripotency array scores. iPSC and ESC lines were maintained in a 5% CO_2 air environment in human iPSC media (Somers et al., 2010). iPSC clones underwent a minimum of 20 passages prior to additional experimentation. Additional details are described in the Supplemental Experimental Procedures.

Directed Endodermal and Hepatic Differentiation of PSCs with Flow Cytometry Sorting/Analysis

Human PSCs were differentiated via the serial introduction of growth factors using previously described endodermal and hepatic differentiation protocols (Cheng et al., 2012). Additional details of the protocol and antibody staining procedures for flow cytometry and cell sorting are described in the Supplemental Experimental Procedures.

ELISA

Secreted AAT and albumin were quantified in the supernatants of differentiated PSCs as previously described (Wilson et al., 2008, 2010). Human albumin was quantified using the Abcam Albumin Human ELISA kit per the manufacturer's instructions.

RNA Isolation and qPCR Analysis

Total RNA and miRNA were isolated from cells using an miRNeasy kit (QIAGEN) according to the manufacturer's instructions. Human fetal liver control RNA was purchased from Clontech Laboratories. Real-time qPCR was performed in triplicate for all samples using either the SYBR Green or TaqMan systems as previously described (Mills et al., 2013; Somers et al., 2010). Additional details are described in the Supplemental Experimental Procedures.

Gene Expression and DNA Methylation Microarray Analysis

Large and small RNAs were labeled with biotin before hybridization to Affymetrix GeneChip Human Gene 1.0 ST or miRNA 2.0 arrays and analyzed using previously published methods (Mills et al., 2013). miRNA 2.0 arrays were normalized to produce probeset-level expression values using the Affymetrix miRNA QC Tool (version 1.1.1.0), using default background detection, RMA global background correction, quantile normalization, and median polish summarization. Quantitative measurement of DNA methylation in study samples was achieved using Illumina's Infinium HD Methylation Assay with HumanMethylation 450 BeadChip arrays. Additional details are described in the Supplemental Experimental Procedures.

Statistical Analysis

PCA was performed using the *prcomp* R function with expression values normalized across all samples to a mean of zero and an SD of one. Differential gene expression was assessed using *limma* (version 3.14.4) by modeling expression as a linear function of cell type, time, and their interaction with *lmFit*, followed by adjustment with *eBayes*. Two-way ANOVA and moderated t tests were then performed using the *limma* functions *topTableF* and *topTable*, respectively. Correction for multiple hypothesis testing was accomplished using Benjamini-Hochberg FDR. All analyses were performed using the R environment for statistical computing.

AAT Pulse-Chase Radiolabeling

iPSC line B-16 and its isogenic, zinc-finger nuclease (ZFN)-corrected daughter iPSC line B-16-C-2 were differentiated to the hepatic stage before labeling with ^{35}S-Met/Cys. The kinetic of AAT post-translational intracellular processing and secretion was then assayed via pulse-chase labeling using previously described methods (Ordóñez et al., 2013). Additional details are described in the Supplemental Experimental Procedures.

Quantification of Autophagic Flux

To quantify autophagosomes in differentiating iPSC-hepatic cells, T9 PiZZ or WT cells were transduced overnight with an LC3-GFP encoding lentivirus at an MOI of 12.5 in differentiation media containing polybrene (5 μg/ml). Cells were then differentiated until T16 before treatment with either CBZ (30 μM) or DMSO vehicle for 48 hr. At T18, live cells were imaged at 37°C, 5% CO2, using a Leica LSM710 confocal microscope (488-nm excitation), and GFP+ punctate autophagosomes were quantified by visual inspection in 50–60 cells per group before and after 4 hr of incubation with bafilomycin (LC Laboratories). LC3 and p62 were further quantified by western blot.

Western Blot

PiZZ or WT iPSCs were differentiated to T16 or T18 before treatment with either CBZ, DMSO vehicle, or regular media as described for each experiment. Cell protein lysates were collected and separated in a 12% polyacrylamide gel before transfer onto a polyvinylidene fluoride (PVDF) membrane. Membranes were probed with antibodies against LC3 (Sigma-Aldrich), p62 (Abnova), B-actin (Sigma-Aldrich), KDEL (Grp78 and Grp94; Enzo Life Sciences), sXBP-1 (BioLegend), IκBα (Cell Signaling Technology), or GAPDH (Millipore). Signal was detected using goat anti-mouse or anti-rabbit HRP substrate (Bio-Rad) on a LAS-4000 luminescent image analyzer (Fuji) and Image J software was utilized to measure densitometry.

PSC Drug Treatment and Analysis

CBZ (Sigma-Aldrich) was added at the concentrations indicated in the text to differentiation media. Cells, RNA, and supernatants were collected 48 hr later for analysis by flow cytometry, qPCR, ELISA, or western blot. To quantify the sensitivity of differentiating iPSC-hepatic cells to hepatotoxic drugs, acetaminophen, aflatoxin B, amiodarone, danazol, or puromycin was added to T20 differentiation media at the indicated concentrations. Cell viability was quantified after 4 hr (acetaminophen) or 4 days (aflatoxin B, amiodarone, danazol, and puromycin) via MTT assay. For 4-day drug exposures, media were changed once at the 48-hr time point.

ACCESSION NUMBERS

The accession number for the array data reported in this paper is GSE66078.

SUPPLEMENTAL INFORMATION

Supplemental Information includes Supplemental Experimental Procedures, five figures, and three tables and can be found with this article online at http://dx.doi.org/10.1016/j.stemcr.2015.02.021.

AUTHOR CONTRIBUTIONS

D.N.K. and P.G. jointly conceived the study. D.N.K., P.G., A.A.W., L.Y., D.A.L., L.V., G.J.M., G.M., O.S.S., and M.I.R. contributed to experimental design. L.Y., A.A.W., M.L., C.-P.S., J.A.M., J.J., G.C.L., D.C.L., and A.O. performed experiments. L.Y., A.A.W., M.L., C.-P.S., S.S.S., A.H.L., J.N., A.C.G., F.-J.M., P.M., A.S., M.I.R., D.N.K., and P.G. analyzed and interpreted experimental data. D.N.K. and A.A.W. wrote and edited the paper.

ACKNOWLEDGMENTS

We are indebted to members of the Kotton, Wilson, and Ikonomou labs and CReM for helpful discussions. For essential technical support, we thank Amel Omari, Amulya Iyer, Peggy Russell, and Emily Porter. We are grateful to Dr. Yuriy Alekseyev of the Boston University Microarray Resource, Sherry Zang and Dr. Gang Liu for microarray processing, and Dr. Tomas Carroll for generous technical assistance. A.A.W. is supported by K08 HL103771, FAMRI 062572_YCSA, an Alpha-1 Foundation Research Grant, and a Boston University Department of Medicine Career Investment Award; P.G., L.Y., and P.M. are supported by NIH 1RC2HL101535-01; M.L. is an Evans Center Fellow and member of the Evans Center for Interdisciplinary Biomedical Research ARC on mitochondria; and D.N.K. is supported by NIH 1RC2HL101535-01, 1R01HL095993, 1R01HL108678, an Alpha-1 Foundation Award, and an ARC award from the Evans Center for Interdisciplinary Research at Boston University.

Received: July 8, 2014
Revised: February 25, 2015
Accepted: February 25, 2015
Published: April 2, 2015

REFERENCES

Baxter, M., Withey, S., Harrison, S., Segeritz, C.-P., Zhang, F., Atkinson-Dell, R., Rowe, C., Gerrard, D.T., Sison-Young, R., Jenkins, R., et al. (2015). Phenotypic and functional analyses show stem cell-derived hepatocyte-like cells better mimic fetal rather than adult hepatocytes. J. Hepatol. *62*, 581–589.

Brantly, M., Nukiwa, T., and Crystal, R.G. (1988). Molecular basis of alpha-1-antitrypsin deficiency. Am. J. Med. *84* (6A), 13–31.

Carroll, T.P., Greene, C.M., O'Connor, C.A., Nolan, A.M., O'Neill, S.J., and McElvaney, N.G. (2010). Evidence for unfolded protein response activation in monocytes from individuals with alpha-1 antitrypsin deficiency. J. Immunol. *184*, 4538–4546.

Cheng, X., Ying, L., Lu, L., Galvão, A.M., Mills, J.A., Lin, H.C., Kotton, D.N., Shen, S.S., Nostro, M.C., Choi, J.K., et al. (2012). Self-renewing endodermal progenitor lines generated from human pluripotent stem cells. Cell Stem Cell *10*, 371–384.

Choi, S.M., Kim, Y., Shim, J.S., Park, J.T., Wang, R.-H., Leach, S.D., Liu, J.O., Deng, C., Ye, Z., and Jang, Y.-Y. (2013). Efficient drug screening and gene correction for treating liver disease using patient-specific stem cells. Hepatology *57*, 2458–2468.

Christodoulou, C., Longmire, T.A., Shen, S.S., Bourdon, A., Sommer, C.A., Gadue, P., Spira, A., Gouon-Evans, V., Murphy, G.J., Mostoslavsky, G., and Kotton, D.N. (2011). Mouse ES and iPS cells can form similar definitive endoderm despite differences in imprinted genes. J. Clin. Invest. *121*, 2313–2325.

Crystal, R.G. (1990). Alpha 1-antitrypsin deficiency, emphysema, and liver disease. Genetic basis and strategies for therapy. J. Clin. Invest. *85*, 1343–1352.

de Serres, F.J. (2002). Worldwide racial and ethnic distribution of alpha1-antitrypsin deficiency: summary of an analysis of published genetic epidemiologic surveys. Chest *122*, 1818–1829.

Ebert, A.D., Yu, J., Rose, F.F., Jr., Mattis, V.B., Lorson, C.L., Thomson, J.A., and Svendsen, C.N. (2009). Induced pluripotent stem cells from a spinal muscular atrophy patient. Nature *457*, 277–280.

Eriksson, S., Carlson, J., and Velez, R. (1986). Risk of cirrhosis and primary liver cancer in alpha 1-antitrypsin deficiency. N. Engl. J. Med. *314*, 736–739.

Graham, K.S., Le, A., and Sifers, R.N. (1990). Accumulation of the insoluble PiZ variant of human alpha 1-antitrypsin within the hepatic endoplasmic reticulum does not elevate the steady-state level of grp78/BiP. J. Biol. Chem. *265*, 20463–20468.

Hidvegi, T., Schmidt, B.Z., Hale, P., and Perlmutter, D.H. (2005). Accumulation of mutant alpha1-antitrypsin Z in the endoplasmic reticulum activates caspases-4 and -12, NFkappaB, and BAP31 but not the unfolded protein response. J. Biol. Chem. *280*, 39002–39015.

Hidvegi, T., Ewing, M., Hale, P., Dippold, C., Beckett, C., Kemp, C., Maurice, N., Mukherjee, A., Goldbach, C., Watkins, S., et al. (2010). An autophagy-enhancing drug promotes degradation of mutant alpha1-antitrypsin Z and reduces hepatic fibrosis. Science *329*, 229–232.

Hitomi, J., Katayama, T., Eguchi, Y., Kudo, T., Taniguchi, M., Koyama, Y., Manabe, T., Yamagishi, S., Bando, Y., Imaizumi, K., et al. (2004). Involvement of caspase-4 in endoplasmic reticulum stress-induced apoptosis and Abeta-induced cell death. J. Cell Biol. *165*, 347–356.

Kajiwara, M., Aoi, T., Okita, K., Takahashi, R., Inoue, H., Takayama, N., Endo, H., Eto, K., Toguchida, J., Uemoto, S., and Yamanaka, S. (2012). Donor-dependent variations in hepatic differentiation from human-induced pluripotent stem cells. Proc. Natl. Acad. Sci. USA *109*, 12538–12543.

Kaneko, M., Niinuma, Y., and Nomura, Y. (2003). Activation signal of nuclear factor-kappa B in response to endoplasmic reticulum stress is transduced via IRE1 and tumor necrosis factor receptor-associated factor 2. Biol. Pharm. Bull. *26*, 931–935.

Lawless, M.W., Greene, C.M., Mulgrew, A.T., Taggart, C.C., O'Neill, S.J., and McElvaney, N.G. (2004). Activation of endoplasmic reticulum-specific stress responses associated with the conformational disease Z alpha 1-antitrypsin deficiency. J. Immunol. *172*, 5722–5726.

Leung, A., Nah, S.K., Reid, W., Ebata, A., Koch, C.M., Monti, S., Genereux, J.C., Wiseman, R.L., Wolozin, B., Connors, L.H., et al. (2013). Induced pluripotent stem cell modeling of multisystemic, hereditary transthyretin amyloidosis. Stem Cell Reports *1*, 451–463.

Lomas, D.A., Evans, D.L., Finch, J.T., and Carrell, R.W. (1992). The mechanism of Z alpha 1-antitrypsin accumulation in the liver. Nature *357*, 605–607.

Mills, J.A., Wang, K., Paluru, P., Ying, L., Lu, L., Galvão, A.M., Xu, D., Yao, Y., Sullivan, S.K., Sullivan, L.M., et al. (2013). Clonal genetic and hematopoietic heterogeneity among human-induced pluripotent stem cell lines. Blood *122*, 2047–2051.

Müller, F.-J., Schuldt, B.M., Williams, R., Mason, D., Altun, G., Papapetrou, E.P., Danner, S., Goldmann, J.E., Herbst, A., Schmidt, N.O., et al. (2011). A bioinformatic assay for pluripotency in human cells. Nat. Methods *8*, 315–317.

Ogawa, S., Surapisitchat, J., Virtanen, C., Ogawa, M., Niapour, M., Sugamori, K.S., Wang, S., Tamblyn, L., Guillemette, C., Hoffmann, E., et al. (2013). Three-dimensional culture and cAMP signaling promote the maturation of human pluripotent stem cell-derived hepatocytes. Development *140*, 3285–3296.

Ordóñez, A., Snapp, E.L., Tan, L., Miranda, E., Marciniak, S.J., and Lomas, D.A. (2013). Endoplasmic reticulum polymers impair luminal protein mobility and sensitize to cellular stress in alpha1-antitrypsin deficiency. Hepatology *57*, 2049–2060.

Pahl, H.L., and Baeuerle, P.A. (1995). A novel signal transduction pathway from the endoplasmic reticulum to the nucleus is mediated by transcription factor NF-kappa B. EMBO J. *14*, 2580–2588.

Pan, S., Huang, L., McPherson, J., Muzny, D., Rouhani, F., Brantly, M., Gibbs, R., and Sifers, R.N. (2009). Single nucleotide polymorphism-mediated translational suppression of endoplasmic reticulum mannosidase I modifies the onset of end-stage liver disease in alpha1-antitrypsin deficiency. Hepatology *50*, 275–281.

Park, I.-H., Arora, N., Huo, H., Maherali, N., Ahfeldt, T., Shimamura, A., Lensch, M.W., Cowan, C., Hochedlinger, K., and Daley, G.Q. (2008). Disease-specific induced pluripotent stem cells. Cell *134*, 877–886.

Perlmutter, D.H., and Pierce, J.A. (1989). The alpha 1-antitrypsin gene and emphysema. Am. J. Physiol. *257*, L147–L162.

Perlmutter, D.H., Brodsky, J.L., Balistreri, W.F., and Trapnell, B.C. (2007). Molecular pathogenesis of alpha-1-antitrypsin deficiency-associated liver disease: a meeting review. Hepatology *45*, 1313–1323.

Rashid, S.T., Corbineau, S., Hannan, N., Marciniak, S.J., Miranda, E., Alexander, G., Huang-Doran, I., Griffin, J., Ahrlund-Richter, L., Skepper, J., et al. (2010). Modeling inherited metabolic disorders of the liver using human induced pluripotent stem cells. J. Clin. Invest. *120*, 3127–3136.

Shan, J., Schwartz, R.E., Ross, N.T., Logan, D.J., Thomas, D., Duncan, S.A., North, T.E., Goessling, W., Carpenter, A.E., and Bhatia, S.N. (2013). Identification of small molecules for human hepatocyte expansion and iPS differentiation. Nat. Chem. Biol. *9*, 514–520.

Si-Tayeb, K., Noto, F.K., Nagaoka, M., Li, J., Battle, M.A., Duris, C., North, P.E., Dalton, S., and Duncan, S.A. (2010). Highly efficient generation of human hepatocyte-like cells from induced pluripotent stem cells. Hepatology *51*, 297–305.

Sjogren, A.-K.M., Liljevald, M., Glinghammar, B., Sagemark, J., Li, X.-Q., Jonebring, A., Cotgreave, I., Brolén, G., and Andersson, T.B. (2014). Critical differences in toxicity mechanisms in induced

pluripotent stem cell-derived hepatocytes, hepatic cell lines and primary hepatocytes. Arch. Toxicol. *88*, 1427–1437.

Smith, B.W., Rozelle, S.S., Leung, A., Ubellacker, J., Parks, A., Nah, S.K., French, D., Gadue, P., Monti, S., Chui, D.H.K., et al. (2013). The aryl hydrocarbon receptor directs hematopoietic progenitor cell expansion and differentiation. Blood *122*, 376–385.

Somers, A., Jean, J.-C., Sommer, C.A., Omari, A., Ford, C.C., Mills, J.A., Ying, L., Sommer, A.G., Jean, J.M., Smith, B.W., et al. (2010). Generation of transgene-free lung disease-specific human induced pluripotent stem cells using a single excisable lentiviral stem cell cassette. Stem Cells *28*, 1728–1740.

Suzuki, T., Mayhew, C., Sallese, A., Chalk, C., Carey, B.C., Malik, P., Wood, R.E., and Trapnell, B.C. (2014). Use of induced pluripotent stem cells to recapitulate pulmonary alveolar proteinosis pathogenesis. Am. J. Respir. Crit. Care Med. *189*, 183–193.

Szkolnicka, D., Farnworth, S.L., Lucendo-Villarin, B., Storck, C., Zhou, W., Iredale, J.P., Flint, O., and Hay, D.C. (2014). Accurate prediction of drug-induced liver injury using stem cell-derived populations. Stem Cells Transl. Med. *3*, 141–148.

Takahashi, K., and Yamanaka, S. (2006). Induction of pluripotent stem cells from mouse embryonic and adult fibroblast cultures by defined factors. Cell *126*, 663–676.

Teckman, J.H., and Perlmutter, D.H. (2000). Retention of mutant alpha(1)-antitrypsin Z in endoplasmic reticulum is associated with an autophagic response. Am. J. Physiol. Gastrointest. Liver Physiol. *279*, G961–G974.

Teckman, J.H., An, J.-K., Loethen, S., and Perlmutter, D.H. (2002). Fasting in alpha1-antitrypsin deficient liver: constitutive activation of autophagy. Am. J. Physiol. Gastrointest. Liver Physiol. *283*, G1156–G1165.

Terrenoire, C., Wang, K., Tung, K.W., Chung, W.K., Pass, R.H., Lu, J.T., Jean, J.C., Omari, A., Sampson, K.J., Kotton, D.N., et al. (2013). Induced pluripotent stem cells used to reveal drug actions in a long QT syndrome family with complex genetics. J. Gen. Physiol. *141*, 61–72.

Twig, G., Elorza, A., Molina, A.J.A., Mohamed, H., Wikstrom, J.D., Walzer, G., Stiles, L., Haigh, S.E., Katz, S., Las, G., et al. (2008). Fission and selective fusion govern mitochondrial segregation and elimination by autophagy. EMBO J. *27*, 433–446.

Wilson, A.A., Kwok, L.W., Hovav, A.-H., Ohle, S.J., Little, F.F., Fine, A., and Kotton, D.N. (2008). Sustained expression of alpha1-antitrypsin after transplantation of manipulated hematopoietic stem cells. Am. J. Respir. Cell Mol. Biol. *39*, 133–141.

Wilson, A.A., Murphy, G.J., Hamakawa, H., Kwok, L.W., Srinivasan, S., Hovav, A.-H., Mulligan, R.C., Amar, S., Suki, B., and Kotton, D.N. (2010). Amelioration of emphysema in mice through lentiviral transduction of long-lived pulmonary alveolar macrophages. J. Clin. Invest. *120*, 379–389.

Wu, Y., Whitman, I., Molmenti, E., Moore, K., Hippenmeyer, P., and Perlmutter, D.H. (1994). A lag in intracellular degradation of mutant alpha 1-antitrypsin correlates with the liver disease phenotype in homozygous PiZZ alpha 1-antitrypsin deficiency. Proc. Natl. Acad. Sci. USA *91*, 9014–9018.

Yusa, K., Rashid, S.T., Strick-Marchand, H., Varela, I., Liu, P.-Q., Paschon, D.E., Miranda, E., Ordóñez, A., Hannan, N.R.F., Rouhani, F.J., et al. (2011). Targeted gene correction of α1-antitrypsin deficiency in induced pluripotent stem cells. Nature *478*, 391–394.

Stem Cell Reports, Volume *4*

Supplemental Information

Emergence of a Stage-Dependent Human Liver Disease Signature with Directed Differentiation of Alpha-1 Antitrypsin-Deficient iPS Cells

Andrew A. Wilson, Lei Ying, Marc Liesa, Charis-Patricia Segeritz, Jason A. Mills, Steven S. Shen, Jyhchang Jean, Geordie C. Lonza, Derek C. Liberti, Alex H. Lang, Jean Nazaire, Adam C. Gower, Franz-Josef Müeller, Pankaj Mehta, Adriana Ordóñez, David A. Lomas, Ludovic Vallier, George J. Murphy, Gustavo Mostoslavsky, Avrum Spira, Orian S. Shirihai, Maria I. Ramirez, Paul Gadue, and Darrell N. Kotton

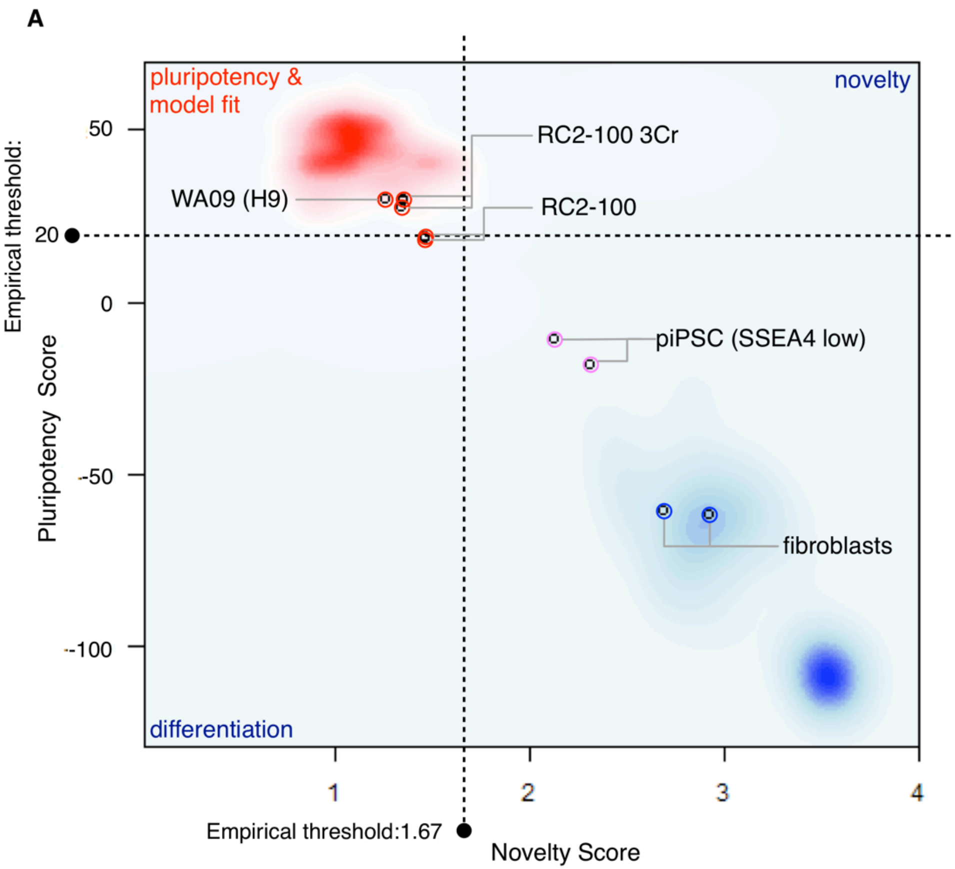

Figure S1 Pluritest scores based on global transcriptomic profiling of PiZZ iPSCs before and after Cre-mediated excision of the STEMCCA reprogramming vector. Pluritest characterization of the transcriptome of PiZZ iPSCs compared to benchmark pluripotent and partially reprogrammed clones demonstrates that PiZZ clones reprogrammed with the floxed STEMCCA polycistronic lentiviral reprogramming cassette have a high pluripotency score and cluster with ESCs as opposed to partially reprogrammed iPSCs. PluriTest (Muller et al. Nat Methods 2011 Mar 6) is a bioinformatic assay for accurate assessment of pluripotency. PluriTest analyzes the expression of a large number of pluripotency associated transcripts with a "Pluripotency Score" and tests for the conformity of a given sample with the global transcriptional profile typical for genetically and epigenetically normal human embryonic and induced pluripotent stem cells with a metric termed "Novelty Score". The PluriTest assay provides a global statistical model for the genome-wide transcriptional landscape of human PSC. PluriTest results were plotted on the empirically defined density distribution for previously referenced pluripotent cells (red cloud) and somatic cells (blue cloud (Müller F-J, Schuldt BM, Williams R, Mason D, Altun G, Papapetrou EP, et al. A bioinformatic assay for pluripotency in human cells. Nat Methods. 2011 Mar 6)

Figure S2

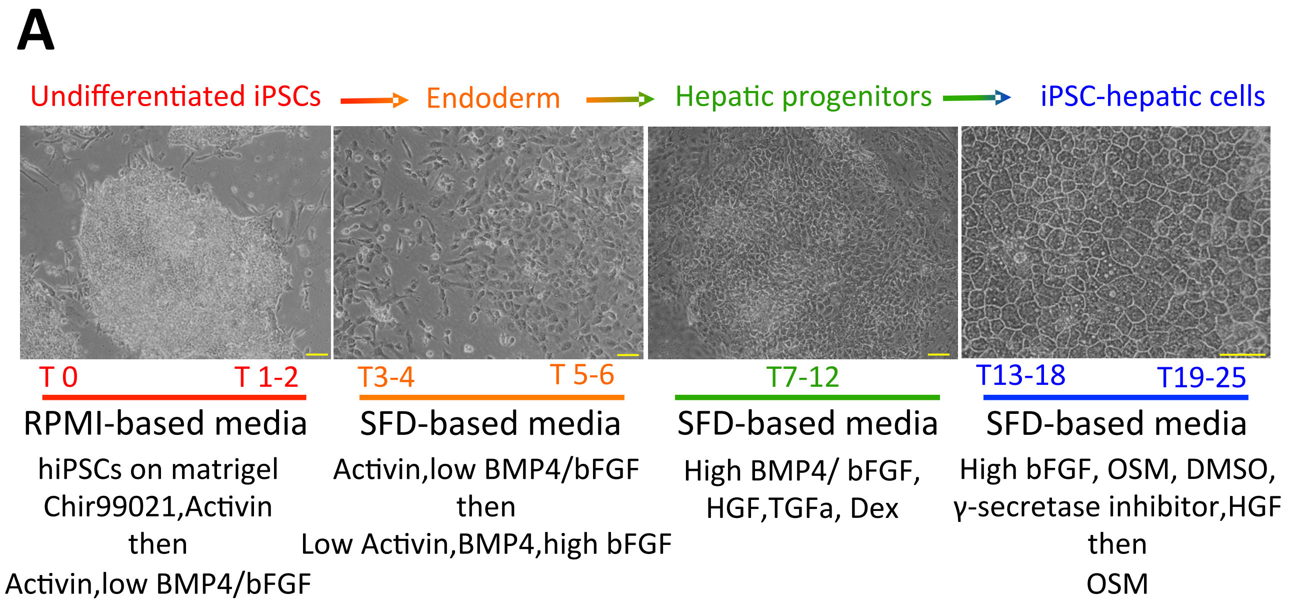

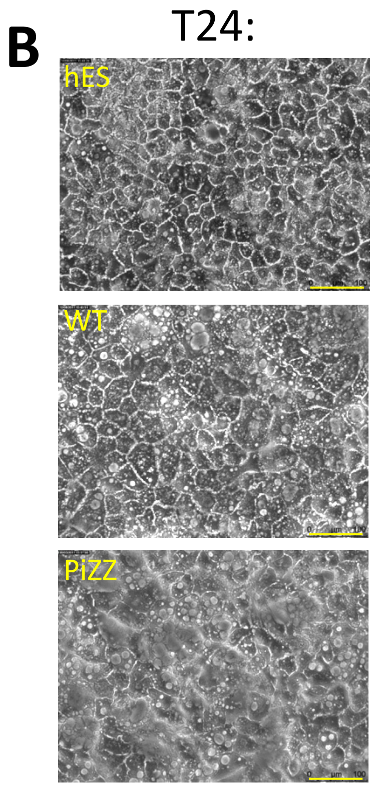

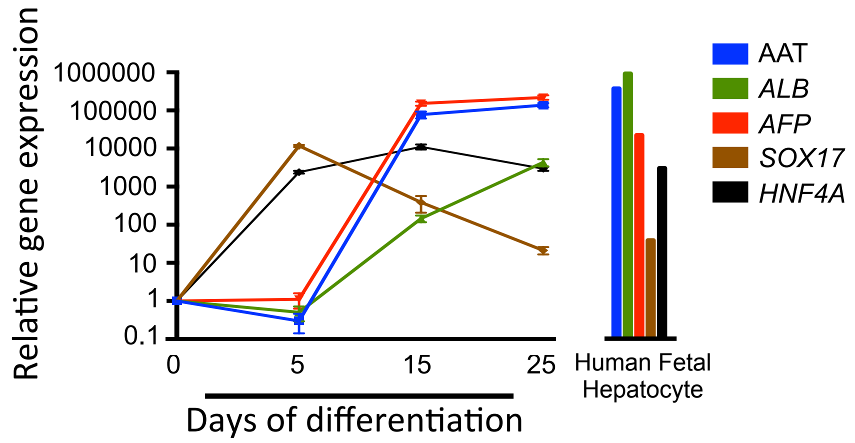

Figure S2 iPSC directed differentiation protocol. (**A**) Undifferentiated iPSCs are passaged off of MEFs onto gelatin-coated dishes 2 days before starting differentiation. At T0, endodermal differentiation is initiated by 1 day of media supplementation with Chir99021 and Activin A. Media is changed daily through T6 and every other day for the remainder of the protocol. The base media is changed to SFD beginning at T3. Scale bars: 100 uM. (**B**) At T24, cells exhibit a characteristic polygonal hepatocyte-like morphology. Scale bars: 100 uM.(**C**) qPCR demonstrates gene expression levels at key developmental time points during differentiation compared to T0 iPSCs. *Sox17*and *HNF4* expression levels are increased at the endodermal stage while expression of hepatic genes, such as AAT, albumin, and *AFP* increases later during differentiation. Gene expression levels at hepatic stage are similar to those seen in human fetal hepatocytes. n = 3 biological replicates. Data are represented as mean +/- SEM.

Figure S3

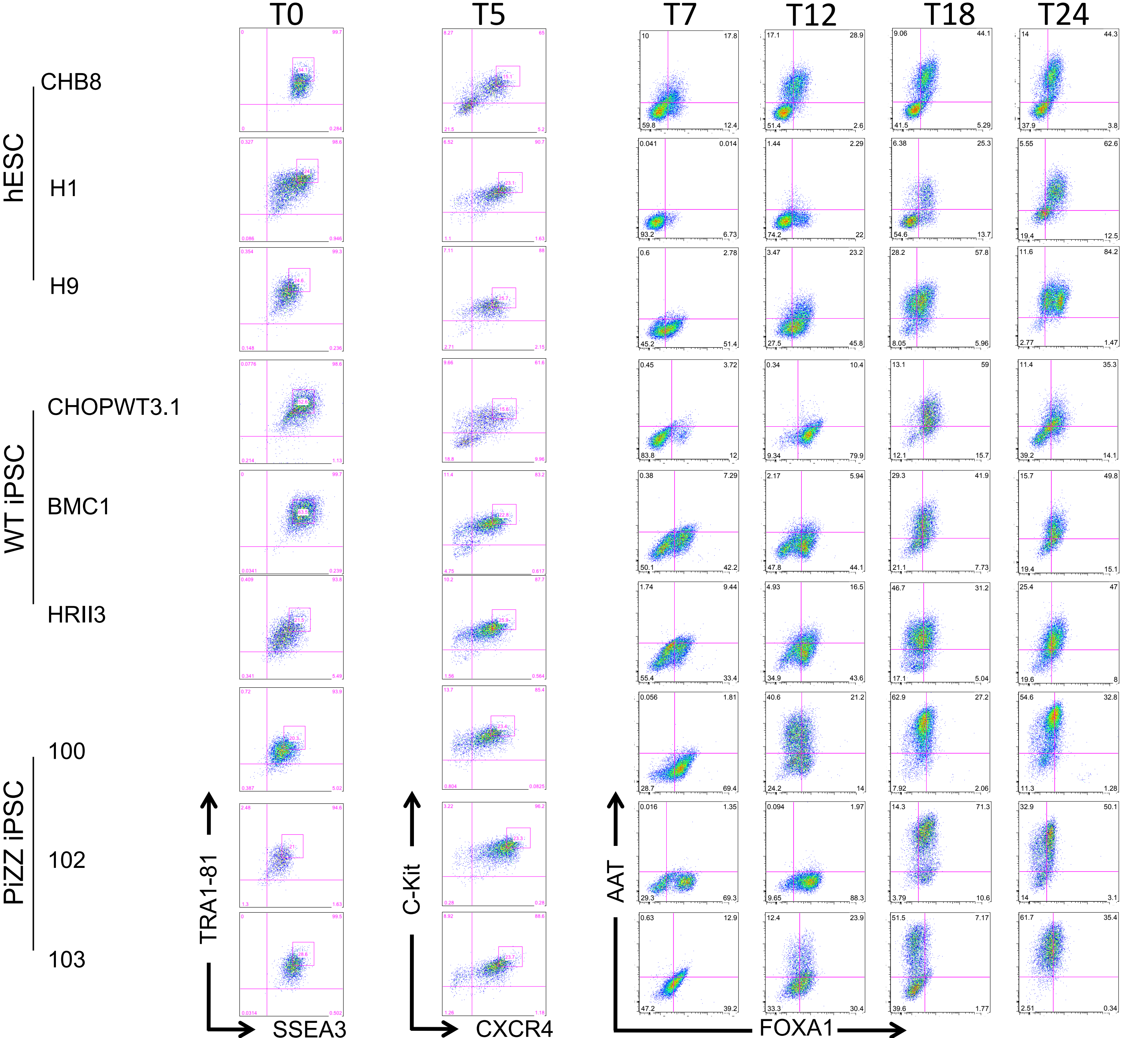

Figure S3 Flow cytometric characterization of ESC, WT iPSC, and PiZZ iPSC lines at key developmental timepoints. All 9 pluripotent stem cell lines were analyzed at the undifferentiated stage (T0), definitive endoderm stage (T5), or during hepatic specification and maturation (T7-T24) with stage-specific markers at each time point. Cells were sorted using the indicated gates for TRA-1-81+/SSEA3+ (T0) or C-kit+/CXCR4+ (T5) cells. The AAT/FOXA1 protein expression kinetic exhibits some line to line variation but all lines achieve high levels of coexpression by T24.

Figure S4

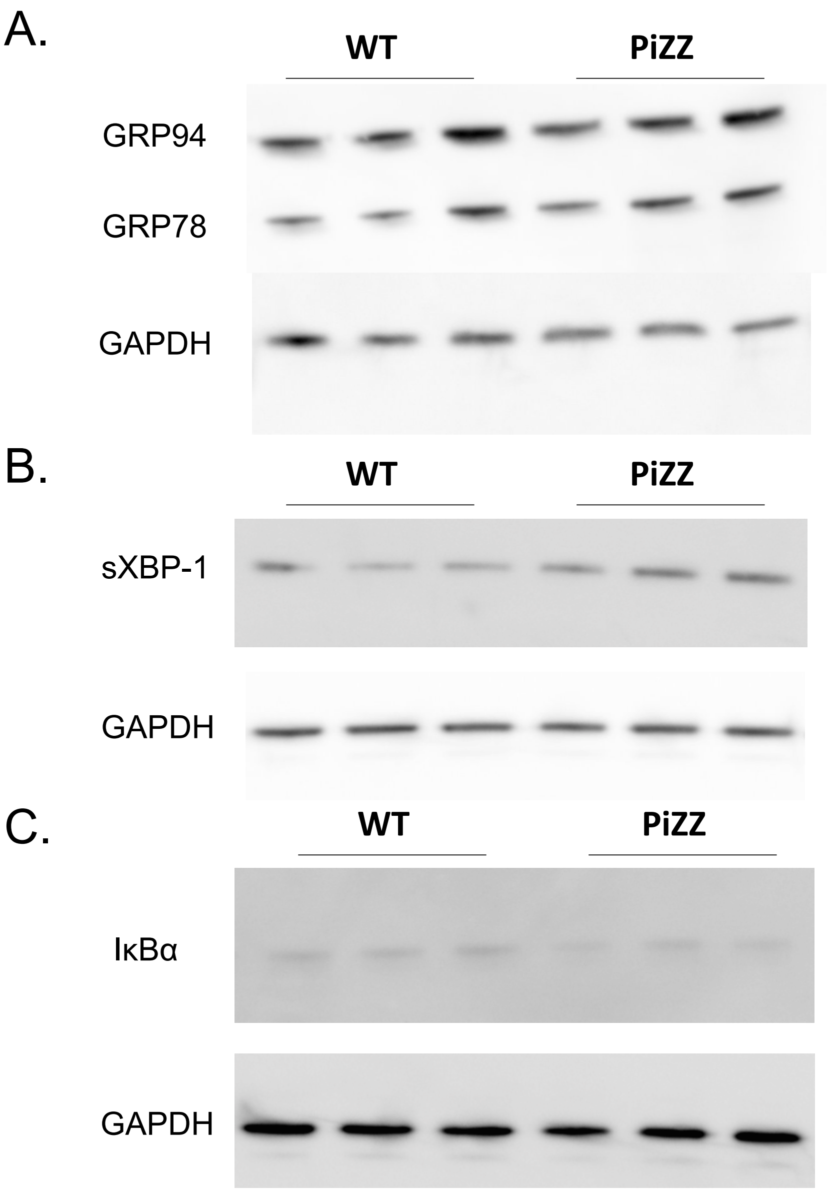

Figure S4 Differential expression of UPR-associated proteins in PiZZ iPSC-hepatic cells. Cell lysates were prepared from triplicate wells of differentiating cells at T16 or T18. Western blots were then performed using primary antibodies against GRP78, GRP94 **(A)**, the mature spliced form of XBP-1 (**B**), and the NF-κB inhibitory protein IκBα. In each case, membranes were strippled and re-probed for GAPDH to control for protein loading.

Figure S5

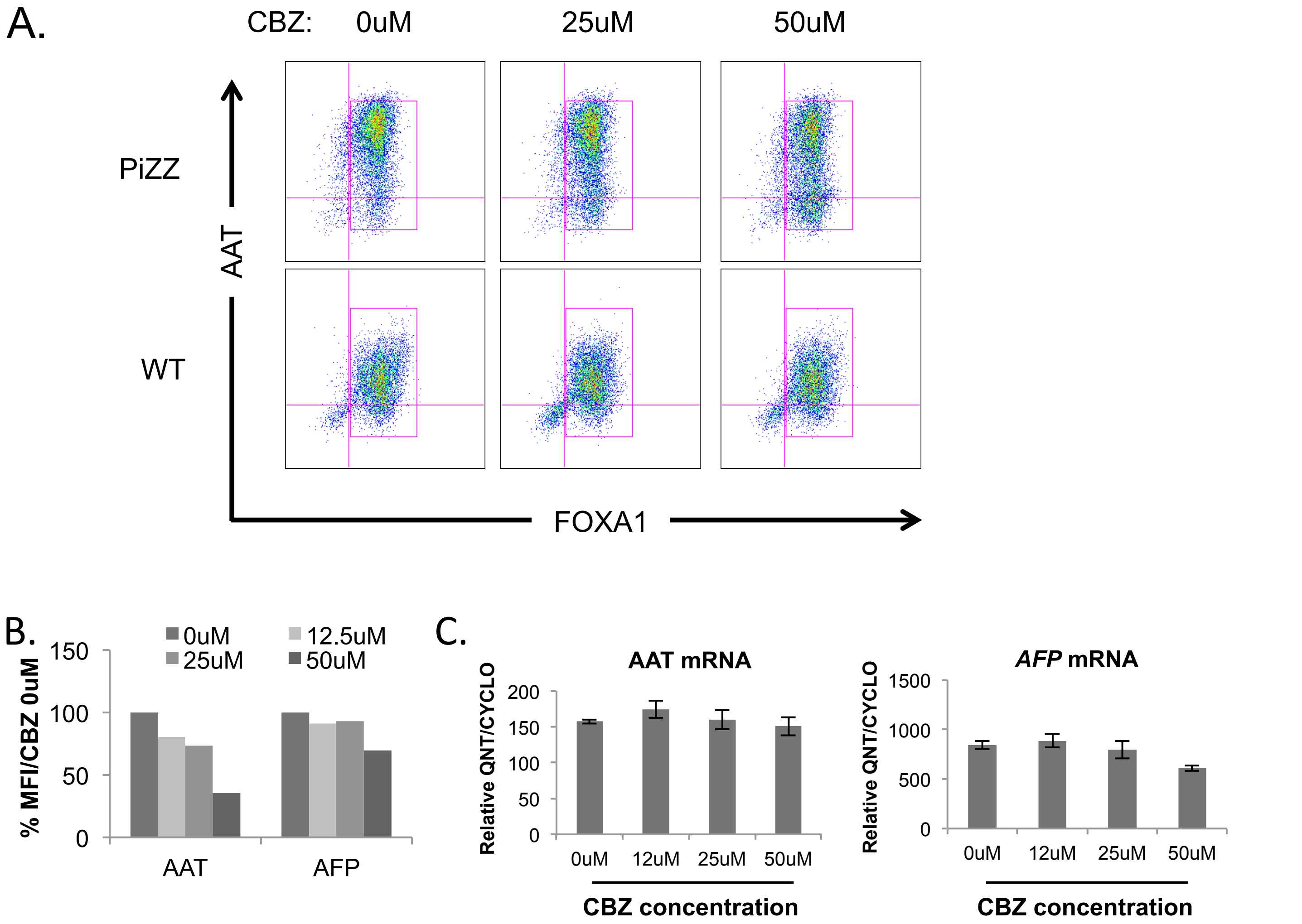

Figure S5 CBZ treatment decreases intracellular AAT in PiZZ iPSC-hepatic cells. (**A**) Flow cytometry demonstrates that the mean fluorescence intensity (MFI) of intracellular AAT antibody staining decreases with CBZ treatment in PiZZ, but not WT, iPSC-hepatic cells. (**B**) MFI of intracellular AAT antibody staining decreases with increasing CBZ concentration in PiZZ cells. This effect is not seen with intracellular AFP. (**C**) CBZ treatment does not affect levels of AAT or *AFP* transcription. n = 3 independent experiments. Data are represented as mean +/- SEM.

Table S1

TABLE: DISEASE SIGNATURE: 2-WAY ANOVA INTERACTION EFFECT; FDR<0.25				
**Gene.Symb**	**adj.P.Val (FDR)**	**logFC Alpha vs iPSC T24**	**P.Value**	**mRNA.Accession**
DNAH5	0.001387322	1.889999333	4.75E-08	NM_001369
CASP4	0.014884331	1.907756	1.46E-06	NM_033306
CFH	0.014884331	2.682600333	1.88E-06	NM_000186
HAVCR2	0.014884331	2.277453333	2.04E-06	NM_032782
ERAP2	0.029128034	2.761159	4.99E-06	NM_022350
KCNJ6	0.04186302	1.373596	8.60E-06	NM_002240
---	0.109360946	1.046387333	2.62E-05	ENST00000474296
WFDC10B	0.116203146	1.470892667	3.58E-05	NM_172006
GRM4	0.116203146	0.798611667	3.61E-05	NM_000841
---	0.116203146	-0.723990667	3.98E-05	NM_033534.1
TFEC	0.14674925	-1.188121667	5.53E-05	NM_012252
---	0.156395992	0.467866333	6.82E-05	ENST00000517114
CA10	0.156395992	0.965961333	6.96E-05	NM_001082533
CGA	0.172961529	-1.958114	8.29E-05	NM_000735
CCL20	0.181722404	2.338906667	9.92E-05	NM_004591
---	0.181722404	0.719536333	0.0001026	ENST00000482613
WIF1	0.181722404	-1.585783	0.0001233	NM_007191
DLG3	0.181722404	-0.57688	0.0001283	NM_021120
---	0.181722404	0.486010667	0.0001304	ENST00000515976
IL4R	0.181722404	0.987948667	0.0001421	NM_000418
RNF133	0.181722404	1.055310333	0.0001536	NM_139175
LOC100133	0.181722404	1.326300667	0.0001538	AY358688
LSM14B	0.181722404	-0.522890667	0.0001562	NM_144703
RNF148	0.181722404	1.341163333	0.0001578	NM_198085
SERPINA3	0.181722404	1.056103667	0.000164	NM_001085
---	0.181722404	0.479801	0.0001648	BC031827
TUBB4	0.181722404	-1.582957333	0.0001749	NM_006087
---	0.181722404	1.277718	0.0001762	ENST00000472474
SQRDL	0.181722404	1.140718333	0.0001829	NM_021199
---	0.181722404	0.622869	0.0002112	ENST00000463780
---	0.181722404	0.622869	0.0002112	ENST00000463780
---	0.181722404	1.922892333	0.0002119	ENST00000517171
DPPA3	0.181722404	-1.757035	0.000214	NM_199286
ROCK1P1	0.181722404	-1.561588333	0.0002168	NR_033770
---	0.181722404	0.604009667	0.0002178	ENST00000517281
---	0.187512805	1.229577333	0.0002405	---
---	0.187512805	0.834795	0.0002476	ENST00000391266
AMY2A	0.187512805	-0.95882	0.0002559	NM_000699
FOXO4	0.187512805	-1.266531333	0.0002681	NM_005938
---	0.187512805	1.068346667	0.000274	---
AMY1A	0.187512805	-0.854025	0.0002829	NM_004038
AMY1A	0.187512805	-0.854025	0.0002829	NM_004038
AMY1A	0.187512805	-0.854025	0.0002829	NM_004038
C1R	0.187512805	0.820498333	0.0002853	NM_001733
ZNF70	0.187512805	-0.585746667	0.0002933	NM_021916
SCN3A	0.187512805	1.276423333	0.0002983	NM_006922
SLAMF8	0.187512805	-0.866773333	0.000314	NM_020125
CFHR1	0.187512805	2.519885667	0.0003146	NM_002113
HSD3BP4	0.187512805	1.100559	0.0003147	NR_033781
ERVFRDE1	0.194279346	-0.507456333	0.0003375	NM_207582
APOL1	0.194279346	2.046024333	0.0003429	NM_145343
OVCH2	0.194279346	2.153060333	0.0003483	NM_198185
GEM	0.194279346	-1.298775667	0.0003621	NM_005261
CCDC151	0.194279346	0.563474667	0.0003631	NM_145045
VNN3	0.194279346	0.810899667	0.0003766	NR_028290
FURIN	0.194279346	1.012876	0.0003783	NM_002569
---	0.194279346	0.544883667	0.0003793	ENST00000470887
STYK1	0.199898667	1.076626333	0.0003971	NM_018423
SPAG5	0.209295324	-1.853380333	0.0004349	NM_006461
LGALS17A	0.209295324	0.614917667	0.0004358	NR_034156
C7orf34	0.209295324	0.572407	0.0004373	NM_178829
---	0.212105936	0.479675333	0.0004525	ENST00000516879
C20orf151	0.212105936	0.511883667	0.0004577	NM_080833
SLC22A12	0.2159028	0.497858667	0.0004732	NM_144585
SOD2	0.219531946	0.871593667	0.0004905	NM_001024465
COMMD9	0.219531946	0.520874333	0.0004989	NM_014186
---	0.219531946	-0.536388333	0.0005112	ENST00000410424
---	0.219531946	0.608298333	0.0005133	ENST00000469282
PRDM16	0.219531946	0.516072667	0.0005188	NM_022114
---	0.221976556	0.588018667	0.0005322	ENST00000490342
---	0.223906356	-0.955998667	0.0005601	ENST00000516933
---	0.223906356	-2.844913333	0.0005653	GENSCAN00000020996
SLC4A8	0.223906356	-2.024568333	0.000566	NM_001039960
---	0.223906356	0.616375333	0.0005711	ENST00000462689
DNAH14	0.223906356	0.708480333	0.0005866	NM_001373
RBMXL3	0.223906356	0.536356333	0.0005871	NM_001145346
LSM14B	0.223906356	-0.608586333	0.0005905	NM_144703
C1QTNF1	0.225500686	1.047790667	0.0006112	NM_030968
SIRPA	0.225500686	0.853482667	0.0006147	NM_001040022
---	0.225500686	0.502454	0.0006216	ENST00000364309
---	0.225500686	-0.706863	0.0006256	ENST00000466549
GDPD2	0.227361845	-1.2859	0.0006473	NM_001171192
VSIG8	0.227361845	0.559231667	0.0006487	NM_001013661
---	0.227361845	0.585405667	0.0006541	ENST00000469816
SCARA3	0.235795427	-1.048902	0.0006946	NM_016240
VNN2	0.235795427	0.542387667	0.0007088	NM_004665
---	0.235795427	0.776246	0.0007091	AB062477
---	0.235795427	-0.806129333	0.0007178	ENST00000485415
ANGPTL4	0.235795427	1.398495333	0.0007187	NM_139314
---	0.237970592	0.681261333	0.0007335	ENST00000384144
---	0.238118688	0.469489	0.0007421	ENST00000517020
RIMBP3	0.239089881	0.417748	0.0007533	NM_015672
BIN1	0.249495739	-1.057385667	0.0007963	NM_139343
---	0.249495739	1.000222667	0.00081	ENST00000411306
MBOAT4	0.249495739	0.486972	0.0008414	NM_001100916
---	0.249495739	0.759812	0.0008696	ENST00000459212
CENPI	0.249495739	-1.301527333	0.0008794	NM_006733
PDZK1IP1	0.249495739	1.262729667	0.0008899	NM_005764
FLJ43763	0.249495739	0.592882333	0.0008916	ENST00000381078
AZGP1	0.249495739	0.897379	0.0008922	NM_001185
TNFSF10	0.249495739	1.447382	0.0008922	NM_003810
BHLHE41	0.249495739	-0.876479667	0.0009045	NM_030762
FAM49A	0.249495739	-1.123131333	0.0009089	NM_030797
ZYG11A	0.249495739	-1.441144333	0.0009157	NM_001004339
LRG1	0.249495739	0.693725667	0.0009164	NM_052972
COX7A1	0.249495739	-0.812038667	0.0009419	NM_001864
C2orf83	0.249495739	0.599935	0.0009563	NM_020161
BATF	0.249495739	0.612902	0.0009624	NM_006399
PSG5	0.249495739	-2.975424333	0.0009645	NM_002781
---	0.249495739	0.549399667	0.0009702	ENST00000484467
EHF	0.249495739	1.758787667	0.0009958	NM_012153
KY	0.249495739	0.474578333	0.0010185	NM_178554
---	0.249495739	0.530830667	0.0010269	ENST00000505282
KIF22	0.249495739	-1.472044	0.0010341	NM_007317
GALNT5	0.249495739	1.475392333	0.0010432	NM_014568
TGM2	0.249495739	0.690833	0.001051	NM_004613
KIF22	0.249495739	-1.478056333	0.0010542	NM_007317
---	0.249495739	0.546366667	0.0010566	---
OR5A2	0.249495739	0.772161667	0.0010634	NM_001001954
---	0.249495739	-1.686442667	0.0010741	NM_199286.2
CPD	0.249495739	0.589138333	0.0010802	NM_001304
ABHD10	0.249495739	-0.79874	0.0010842	NM_018394
GCM1	0.249495739	-0.595818667	0.0010876	NM_003643
RIMBP3	0.249495739	0.441950667	0.0010905	NM_015672
---	0.249495739	0.506613	0.0010976	ENST00000516864
PLEK2	0.249495739	1.571681	0.0011055	NM_016445
NUSAP1	0.249495739	-1.449468333	0.0011097	NM_016359
---	0.249495739	1.235091667	0.0011099	ENST00000365415
---	0.249495739	0.429498	0.0011166	ENST00000486780
---	0.249495739	0.977655667	0.0011282	AK097085
FLJ36840	0.249495739	0.942958333	0.0011298	AK094159
CDCA7	0.249495739	-1.021459667	0.0011363	NM_031942
---	0.249495739	0.640481667	0.0011393	ENST00000364488
---	0.249495739	0.901475667	0.0011515	ENST00000493687
TNFAIP6	0.249495739	-1.582938	0.0011536	NM_007115

Table S1: PiZZ disease-specific transcriptomic signature. Post-hoc moderated t testing of the “interaction effect” identified 135 differentially expressed transcripts at T24 in PiZZ diseased versus normal iPSC-hepatic cells.

	miRNA			methylation		
**PiZZ:ESC/WT**	**T0**	**T5**	**T24**	**T0**	**T5**	**T24**
**Up**	0	0	0	20	37	150
**Down**	0	0	0	3	39	45
**Unchanged**	638	638	638	322311	322258	322139

Table S2: Summary of changes in miRNA expression and CpG methylation between diseased and normal pluripotent stem cells at key developmental stages. PiZZ iPSCs are compared to WT iPSCs and ESCs. FDR adjusted q values are <0.001 (miRNA), and <0.05 (methylation).

Table S3

		Gene Expression			Methylation			Correlation	
GeneSymbol	GeneName	Fold Change	P Value	FDR-adjusted P	ID Methylation Site	Fold Change	P Value	Pearson Correlation	P Value
BATF	basic leucine zipper transcription factor, ATF-like	1.53	9.62E-04	0.249	cg09937039	-1.81	6.91E-04	-0.91	1.3E-02
BATF	basic leucine zipper transcription factor, ATF-like	1.53	9.62E-04	0.249	cg14266927	-1.93	2.81E-03	-0.86	2.9E-02
BATF	basic leucine zipper transcription factor, ATF-like	1.53	9.62E-04	0.249	cg14424070	-3.38	5.55E-03	-0.89	1.7E-02
BATF	basic leucine zipper transcription factor, ATF-like	1.53	9.62E-04	0.249	cg15645309	-1.82	1.48E-02	-0.86	2.9E-02
BATF	basic leucine zipper transcription factor, ATF-like	1.53	9.62E-04	0.249	cg21531300	-2.85	3.60E-03	-0.91	1.1E-02
BATF	basic leucine zipper transcription factor, ATF-like	1.53	9.62E-04	0.249	cg22995449	-2.02	1.57E-03	-0.83	3.9E-02
BATF	basic leucine zipper transcription factor, ATF-like	1.53	9.62E-04	0.249	cg23723793	-1.92	4.03E-03	-0.91	1.2E-02
C1QTNF1	C1q and tumor necrosis factor related protein 1	2.07	6.11E-04	0.226	cg14020904	-1.33	1.36E-02	-0.92	9.5E-03
C1QTNF1	C1q and tumor necrosis factor related protein 1	2.07	6.11E-04	0.226	cg17758081	-1.29	7.56E-03	-0.85	3.0E-02
C1R	complement component 1, r subcomponent	1.77	2.85E-04	0.188	cg08799922	-1.43	2.02E-02	-0.85	3.1E-02
CFHR1	complement factor H-related 1	5.74	3.15E-04	0.188	cg12687463	-1.56	2.73E-03	-0.83	4.3E-02
GRM4	glutamate receptor, metabotropic 4	1.74	3.61E-05	0.116	cg08969344	-1.20	2.48E-02	-0.89	1.7E-02
HAVCR2	hepatitis A virus cellular receptor 2	4.85	2.04E-06	0.015	cg19110684	-1.41	5.11E-03	-0.87	2.4E-02
KCNJ6	potassium inwardly-rectifying channel, subfamily J, member 6	2.59	8.60E-06	0.042	cg03531951	-1.30	1.40E-02	-0.87	2.6E-02
LRG1	leucine-rich alpha-2-glycoprotein 1	1.62	9.16E-04	0.249	cg03882382	-1.84	1.52E-03	-0.81	4.8E-02
LRG1	leucine-rich alpha-2-glycoprotein 1	1.62	9.16E-04	0.249	cg17272620	-1.39	7.14E-03	-0.96	2.1E-03
LRG1	leucine-rich alpha-2-glycoprotein 1	1.62	9.16E-04	0.249	cg22375763	-1.48	1.67E-02	-0.89	1.6E-02
MBOAT4	membrane bound O-acyltransferase domain containing 4	1.40	8.41E-04	0.249	cg00940560	-1.51	3.53E-02	-0.85	3.2E-02
MBOAT4	membrane bound O-acyltransferase domain containing 4	1.40	8.41E-04	0.249	cg21058822	-2.60	2.71E-02	-0.94	5.2E-03
PRDM16	PR domain containing 16	1.43	5.19E-04	0.22	cg04873098	-1.40	3.80E-02	-0.96	2.6E-03
PRDM16	PR domain containing 16	1.43	5.19E-04	0.22	cg15519786	-2.01	3.18E-02	-0.83	4.0E-02
PRDM16	PR domain containing 16	1.43	5.19E-04	0.22	cg17220278	-1.19	4.14E-02	-0.89	1.6E-02
PRDM16	PR domain containing 16	1.43	5.19E-04	0.22	cg21789941	-1.70	2.62E-03	-0.85	3.0E-02
SLC22A12	solute carrier family 22 (organic anion/urate transporter), member 12	1.41	4.73E-04	0.216	cg07220939	-1.59	3.87E-03	-0.95	3.4E-03
TNFSF10	tumor necrosis factor (ligand) superfamily, member 10	2.73	8.92E-04	0.249	cg08144586	-1.28	8.60E-03	-0.87	2.6E-02
VSIG8	V-set and immunoglobulin domain containing 8	1.47	6.49E-04	0.227	cg21574855	-1.46	5.12E-03	-0.85	3.3E-02

Table S3 Anticorrelation of gene expression and CpG methylation in genes comprising the PiZZ disease signature. 13 of the 135 genes comprising the signature exhibit differential methylation at the indicated sites that is decreased in PiZZ iPSC-hepatic cells in comparison with WT and anticorrelated with gene expression (increased in PiZZ iPSC-hepatic cells compared to WT).

Supplemental Experimental Procedures:

Production of iPSCs from human fibroblasts. Normal human dermal fibroblast-derived iPSCs were generated by reprogramming with a single-integrated excisable copy of the floxed hSTEMCCA lentiviral reprogramming vector(Somers et al., 2010) followed by excision with transient Cre recombinase-exposure. These three lines generated from three separate individuals have been previously characterized, published, and named (BMC1, CHOPWT3.1, and HRII-3(Mills et al., 2013; Somers et al., 2010; Terrenoire et al., 2012). PiZZ fibroblasts, isolated by 6-mm full thickness arm skin punch biopsy from volunteer subjects with AAT deficiency as previously described(Somers et al., 2010), were grown in DMEM with 10% FBS. The recruitment of human subjects and all iPSC studies were approved by the Boston University Institutional Review Board (BUMC IRB H-27636). For reprogramming, fibroblasts were transduced with the humanized hSTEMCCA lentiviral reprogramming vector(Mills et al., 2013; Somers et al., 2010). iPSC colonies were mechanically isolated 30 days after transduction and expanded on MEF feeders in human iPSC media. Integrated hSTEMCCA copy number was assessed by Southern blot of gDNA extracts as previously published(Somers et al., 2010), and only iPSC clones with single copy hSTEMCCA integrations were selected for vector excision and further study.

iPSC expansion and characterization. iPSC clones were passaged and expanded in hiPSC media for a minimum of 20 passages prior to additional experimentation. Clones were characterized for expression of genes associated with pluripotency and teratoma assays performed as previously published to confirm functional pluripotency(Somers et al., 2010). Where indicated in the text and supplement, global transcriptomes were scored by Pluritest assay to obtain pluripotency array scores(Müller et al., 2011).

Cre-mediated hSTEMCCA excision. The single copy hSTEMCCA lentiviral cassette was removed from each iPSC clone via transient transfection of pHAGE2-Cre-IRES-PuroR plasmid DNA using Hela Monster transfection reagent (Mirus, Madison, WI, www.mirusbio.com) according to the manufacturer's instructions(Somers et al., 2010). Approximately 11-14 days later, colonies were picked and gDNA from each subclone screened for vector excision by PCR using the following primers and conditions: cMYC F5'-GGA ACT CTT GTG CGT AAG TCG ATA G-3'; WPRE R5'-GGA GGC GGC CCA AAG GGA GAT CCG-3'; 95° C for 3 minutes; followed by 33 cycles of 94°C for 30 seconds, 60°C for 30 seconds, and 72°C for 1 minute; followed by a single cycle of 72°C for 5 minutes. Vector excision was then confirmed by Southern blot using BamHI digested gDNA probed for the WPRE element as previously published(Somers et al., 2010).

Cell culture and maintenance of PSCs. iPSC and ESC lines were maintained in "hiPSC Media" composed of DMEM/F12 (Sigma-Aldrich) with 20% KnockOut Serum Replacement (Invitrogen), 1mM nonanimal L-glutamine (Sigma-Aldrich), 0.1mM B-mercaptoethanol, and 10 ng/ml FGF2 (R&D Systems) on 0.1% gelatin (Sigma-Aldrich) coated plates preseeded with mitomycin C-inactivated or irradiated mouse embryonic fibroblast (MEF) feeder cells. Cells were maintained in a 5% CO_2 air environment.

Directed Endodermal and Hepatic Differentiation of PSCs. Human PSCs were differentiated using previously described protocols(Cheng et al., 2012). For endodermal differentiation, cells were passed onto matrigel-coated dishes at 80% confluency. On the following day, designated "T0", differentiation was induced by culture in media containing growth factors listed below. From T0-T6, differentiation media included 2mM l-glutamine, and 4.5×10^{-4} M monothioglycerol (MTG). Cells were grown in T0 media, consisting of RPMI-based serum-free medium with Chir

99021 (2ug/ml) and Activin A (100 ng/ml), for one day. On days “T1-2”, medium was changed to RPMI with BMP4 (0.5 ng/ml), FGF2 (10ng/ml), Activin A (100 ng/ml), and VEGF (10 ng/ml). On days “T3-4”, cells were cultured in SFD media(Gouon-Evans et al., 2006) with BMP4 (0.5 ng/ml), FGF2 (10ng/ml), Activin A (100 ng/ml), and VEGF (10 ng/ml). For hepatic differentiation, PSCs were differentiated as monolayer cultures as outlined above to generate definitive endoderm and then further differentiated for an additional 3 weeks in SFD-based media with ascorbic acid (50mcg/ml), monothioglycerol ($4.5x10^{-4}$ M), and the following supplements: T7-12: BMP4 (50 ng/ml), FGF2 (10 ng/ml), VEGF (10 ng/ml), EGF (10ng/ml), TGFa (20 ng/ml), HGF (100 ng/ml), and 0.1 uM Dexamethasone; T13-18: FGF2 (10 ng/ml), VEGF (10 ng/ml), EGF (10 ng/ml), HGF (100 ng/ml), Oncostatin M (20 ng/ml), Vitamin K (6 ug/ml), 1.5 uM gamma secretase inhibitor, 0.1 uM Dexamethasone, and 1% DMSO; T19-24: HGF (100 ng/ml), Oncostatin M (20 ng/ml), Vitamin K (6 ug/ml), and 0.1 uM Dexamethasone. Differentiating human PSCs were maintained in a 5% CO_2, 5% O_2, 90% N_2 environment.

Flow cytometry and cell sorting. PSCs were stained with antibodies for the following cell surface antigens: CD117-APC (Invitrogen, #11705), CXCR4-PE (Invitrogen, #MHCXCR404), TRA-1-81-Alexa Fluor 647 (Biolegend, #330706), and SSEA3-AlexaFlour488 (Biolegend, #330306). For staining of intracellular antigens, cells were fixed in 1.6% paraformaldehyde for 20 minutes at 37°C and then permeabilized in saponin buffer (Biolegend). Fixed cells were stained with antibodies against human FOXA1 (Santa Cruz, #sc-101058), AAT (Santa Cruz, #sc-59438), or AFP (R&D, # MAB1368) followed by goat anti-mouse IgG2a-DyLight488 (Jackson ImmunoResearch, # 115-485-206), IgG1-DyLight649 (Jackson ImmunoResearch, #705-496-147) or IgG-AlexaFluor647(Jackson ImmunoResearch, # 115-605-205) antibodies. For all flow cytometry experiments, gating was based on isotype-stained controls. Staining was quantified using a FACSCantos II flow cytometer (BD Biosciences) and analyzed with FlowJo software (Tree Star Inc). To obtain cell pure populations for analysis, T0 and T5 cells were first sorted by flow sorting SSEA3/TRA-1-81 double positive (T0) or CD117/CXCR4 double positive (T5) cells on a FACS Aria II (Becton Dickenson) sorter. Cells were resuspended in Qiazol Lysis Reagent (Qiagen) and snap frozen before storage at -80°C.

Gene expression microarray analysis. Biotin labeling of large RNA was performed using the Ambion WT Expression Kit (Life Technologies, Grand Island, NY) according to the manufacturer's protocol, followed by the GeneChip WT Terminal Labeling and Controls Kit (Affymetrix, Santa Clara, CA). Biotin labeling of small RNA was performed using the Affymetrix FlashTag Biotin HSR RNA Labeling Kit according to the manufacturer's protocol. Labeled, fragmented DNA was then hybridized to Affymetrix GeneChip Human Gene 1.0 ST or miRNA 2.0 arrays, and after staining, microarrays were immediately scanned using an Affymetrix GeneArray Scanner 3000 7G Plus. Human Gene 1.0 ST arrays were normalized to produce gene-level expression values using the implementation of the Robust Multiarray Average (RMA) in the Affymetrix Expression Console software package. miRNA 2.0 arrays were normalized to produce probeset-level expression values using the Affymetrix miRNA QC Tool (version 1.1.1.0), using default background detection, RMA global background correction, quantile normalization, and median polish summarization. Analysis was limited to the 1,105 human microRNAs interrogated by the array. Human Gene 1.0 ST and miRNA 2.0 files have been desposited in the Gene Expression Omnibus (GEO accession number GSE66078).

DNA methylation microarray analysis. Quantitative measurement of DNA methylation in study samples was achieved using Illumina’s Infinium HD Methylation Assay with HumanMethylation 450 BeadChip arrays. Briefly, 500ng gDNA extracted from hESCs, iPSCs derived from normal individuals, and iPSCs from alpha-1 antitrypsin deficient patients underwent bisulfite conversion of unmethylated cytosine bases to uracil. Bisulfite converted DNA was then amplified and

purified prior to overnight hybridization to BeadChip arrays. Next day staining of hybridized arrays produced methylation-dependent differential fluorescence that was detected via an Illumina iScan array scanner. Arrays were visualized and processed using the GenomeStudio software package, which produced IDAT files that were read into a MethyLumiSet using the *methylumIDAT* function in the *methylumi* R package (version 2.4.0). This object was then coerced to a MethyLumiM object, quantile-normalized by sequentially applying the *lumiMethyC* and *lumiMethyN* functions from the *lumi* R package (version 2.10.0), and annotated using the *IlluminaHumanMethylation450k.db* R package (version 1.4.7). Differential methylation between wildtype and PiZZ iPSCs at stage T24 was assessed using the *limma* R package (version 3.14.4) by modeling M values as a linear function of genotype with *lmFit*, followed by empirical Bayesian adjustment with *eBayes* and a moderated *t* test performed using *topTable*. Methylation and expression probesets were matched on gene symbols in order to compute Pearson correlation coefficients between M values and log2 (expression) values from iPSCs at stage T24. All methylation analyses were performed using the R environment for statistical computing (version 2.15.1). Illumina 450K files have been desposited in the Gene Expression Omnibus (GEO accession number GSE66078).

*RNA isolation and quantitative PCR analysis.*Total RNA and miRNA were isolated from cells using an miRNeasy kit (Qiagen) with the optional column RNAse-free DNase treatment, according to the manufacturer's instructions. 200 nanograms to one microgram of RNA was reverse transcribed into cDNA using random hexamers with Superscript III Reverse Transcriptase (Invitrogen). Real-time, quantitative PCR (qPCR) was performed in triplicate for all samples using either SYBR Green QPCR master mix with the Light Cycler 480II qPCR System (Roche, Indianapolis, IN, www.roche.com) or TaqMan primers and master mix with a StepOne Real Time PCR system (Applied Biosystems, Carlsbad, CA, www.lifetechnologies.com). For SYBR Green qPCR, a 10-fold gDNA dilution series ranging from 0.1 to10 ng per reaction was used to evaluate the efficiency of the PCR and calculate the copy number of each gene relative to the housekeeping gene Cyclophilin. Calculated expression levels for each indicated gene were then reported as number of molecules of RNA for that gene per number of molecules of cyclophilin, as previously published(Mills et al., 2013; Somers et al., 2010). Primer sequences are: *CYCLOPHILIN*: F' GAA GAG TGC GAT CAA GAA CCC ATG AC, R' GTC TCT CCT CCT TCT CCT CCT ATC TTT ACT T; AAT(*SERPINA1*): F'AGG GCC TGA AGC TAG TGG ATA AGT, R' TCT GTT TCT TGG CCT CTT CGG TGT; *AFP*: F' CTA CCT GCC TTT CTG GAA GAA CTT TG, R'TCT GTT TCT TGG CCT CTT CGG TGT.

AAT pulse-chase radiolabeling. The kinetic of AAT post-translational intracellular processing and secretion was assayed via pulse-chase radiolabeling. Before radiolabelling, the patient-derived iPSC line B-16 and its isogenic, zinc finger nuclease-corrected daughter iPSC line B-16-C-2 were differentiated to hepatic stage and assayed via pulse-chase labelling using previously described methods(Ordóñez et al., 2013). Briefly, cells were starved in methionine (Met)- and cysteine (Cys)-free pulse medium for 90 min and then incubated with 1.3MBq of ^{35}S-Met/Cys for 30 min at 37°C to allow incorporation of radioactive amino acids. Cells were then washed and incubated in L-Met and L-Cys-supplemented chase medium for the time intervals indicated in the text before collection of cell supernatants and cell harvesting. Total protein was isolated from cell lysates using Nonidet lysis buffer (150mM NaCl, 50mM Tris-HCl pH 7.5, 1% Nonidet P-40) containing 25mM protease inhibitor mixture, followed by centrifugation. AAT was immunoprecipitated from lysates or from supernatants with a polyclonal anti-human α_1-antitrypsin antibody generated by the group of Prof. D.A. Lomas(Miranda et al., 2010) and resolved by 10% v/v SDS-PAGE. Radiolabelled AAT was visualized and quantified on a Cyclone Phosphor Imager (Packard Instrument Co.). Total densitometric value of the combined

lysate and supernatant at each time point were set at 100% and the value of each component displayed as a percent of the total.

Western Blot. PiZZ or WT iPSCs were differentiated to T16 or T18 before treatment with either CBZ, DMSO vehicle, or regular media as described for each experiment. Cell protein lysates were collected and separated in a 12% polyacrylamide gel before transfer onto a PVDF membrane. Membranes were probed with antibodies against LC3 (Sigma-Aldrich, # L7543), p62 (Abnova, #H00008878-M0), B-actin (Sigma-Aldrich, #A5316), KDEL (Grp78, Grp94; Enzo Life Sciences, # SPA-827-F), sXBP-1 (Biolegend, #619502), IκBα (Cell Signaling Technology, #9242), or GAPDH (Millipore, # MAB374). Signal was detected using goat anti-mouse or anti-rabbit HRP substrate (Biorad) on a LAS-4000 luminescent image analyzer (Fuji) and Image J software was utilized to measure densitometry.

Supplemental References:

Gouon-Evans, V., Boussemart, L., Gadue, P., Nierhoff, D., Koehler, C.I., Kubo, A., Shafritz, D.A., and Keller, G. (2006). BMP-4 is required for hepatic specification of mouse embryonic stem cell-derived definitive endoderm. Nat Biotechnol *24*, 1402–1411.

Miranda, E., Pérez, J., Ekeowa, U.I., Hadzic, N., Kalsheker, N., Gooptu, B., Portmann, B., Belorgey, D., Hill, M., Chambers, S., et al. (2010). A novel monoclonal antibody to characterize pathogenic polymers in liver disease associated with α 1-antitrypsin deficiency. Hepatology *52*, 1078–1088.
